# Feedback loop between hepatocyte nuclear factor 1α and endoplasmic reticulum stress mitigates liver injury by downregulating hepatocyte apoptosis

**DOI:** 10.1038/s41598-022-15846-8

**Published:** 2022-07-08

**Authors:** Si-Ying Liu, Jian-Xu Rao, Jie Deng, Gui-Juan Zhang, Xiao-Ling Jiang, Jing Cheng, Huan Chen, Zhi-Gang Jiang, De-Lin Xu, Yi-Huai He

**Affiliations:** 1 Department of Infectious Diseases, The Affiliated Hospital of Zunyi Medical University, No. 201 Dalian Street, Zunyi, 563000 Guizhou China; 2 School of Public Health, Zunyi Medical University, Zunyi, 563099 Guizhou China; 3 Cell Biology Department, Zunyi Medical University, Zunyi, 563099 Guizhou China

**Keywords:** Gastroenterology, Cell biology, Cell death, Gastrointestinal diseases

## Abstract

Hepatocyte nuclear factor alpha (HNF1α), endoplasmic reticulum (ER) stress, and hepatocyte apoptosis contribute to severe acute exacerbation (SAE) of liver injury. Here, we explore HNF1α–ER stress-hepatocyte apoptosis interaction in liver injury. LO2, HepG2 and SK-Hep1 cells were treated with thapsigargin (TG) or tunicamycin (TM) to induce ER stress. Carbon tetrachloride (CCl_4_) was used to induce acute liver injury in mice. Low-dose lipopolysaccharide (LPS) exacerbated liver injury in CCl_4_-induced mice. Significant apoptosis, HNF1α upregulation, and nuclear factor kappa B (NF-κB) activation were observed in human-derived hepatocytes during ER stress. Knockdown of *Rela*, NF-κB p65, inhibited the HNF1α upregulation. Following CCl_4_ treatment ER stress, apoptosis, HNF1α expression and RelA phosphorylation were significantly increased in mice. HNF1α knockdown reduced activating transcription factor 4 (ATF4) expression, and aggravated ER stress as well as hepatocyte apoptosis in vivo and in vitro. The double fluorescent reporter gene assay confirmed that HNF1α regulated the transcription of *ATF4* promoter. LPS aggravated CCl_4_-induced liver injury and reduced HNF1α, and ATF4 expression. Therefore, in combination, HNF1α and ER stress could be mutually regulated forming a feedback loop, which helps in protecting the injured liver by down-regulating hepatocyte apoptosis. Low-dose LPS aggravates hepatocyte apoptosis and promotes the SAE of liver injury by interfering with the feedback regulation of HNF1α and ER stress in acute liver injury.

## Introduction

Severe acute exacerbation (SAE) of liver diseases is an early stage of the acute or chronic form of liver failure that often results from cirrhosis following viral infection, excessive alcohol consumption, or exposure to toxins^1, 2^. Despite advances in treatment strategies, patients with liver failure have high morbidity and mortality rates due to late diagnosis, severe complications in the advanced disease stage, and the limited availability of donor organs^3^. Therefore, understanding the mechanism of SAE in liver injury and its early symptoms could result in a timely diagnosis that could improve the patient prognosis. The SAE of liver diseases is usually attributed to various risk factors that include underlying medical conditions, bacterial or fungal infection, and alcohol consumption^4, 5^. In addition, the aggravation of hepatocyte damage is correlated to the severity of liver injury^6^. Hepatocyte death controls the development and outcome of most liver diseases^7^. Therefore, improving hepatocyte resistance to apoptotic signals could be crucial for maintaining liver function and delaying the SAE of liver injury. Hepatocyte nuclear factor 1α (HNF1α) and endoplasmic reticulum (ER) stress contribute to a protective response in hepatocytes^8, 9^, which might influence the SAE of liver injury.

ER stress is induced by various injury factors, and it is correlated to liver disease pathology^10^. ER stress involves the unfolded protein response (UPR) that is triggered by the aggregation of unfolded or misfolded proteins in the ER lumen^11^. Similarly, ER stress promotes an inflammatory response, such as the activation of nuclear factor kappa B (NF-κB)^12, 13^. Activated transcription factor 6 (ATF6), inositol requiring enzyme 1 (IRE1), and protein kinase R-like kinase (PERK) are crucial for the regulation of UPR^14^. On ATF6 activation, X-box binding protein-1 (XBP1) is upregulated^15^. Subsequently, XBP1 mRNA is alternatively spliced by the active IRE1 that results in the translation of the spliced XBP1 form (XBPls), which then promotes ER stress-related gene expression^16^. PERK activation phosphorylates the eukaryotic translational initiation factor 2 alpha (eIF2α), which attenuates the overall protein translation and decreases the ER burden^17^. In addition, eIF2α selectively initiates the expression of activated transcription factor 4 (ATF4), which induces glucose-regulated protein 78 (GRP78) expression. Under physiological conditions, GRP78 inhibits the activation of IRE1, PERK, and ATF6 signaling pathways^18, 19^. Previously, it has been reported that ER stress increases multidrug-resistance protein 2 (MRP2) expression by activating NF-κB signaling^20^. The upregulation of MRP2 controls acute liver injury through a negative feedback mechanism that reduces ER stress. In addition, the inhibition of eIF2α dephosphorylation reduced hepatocyte apoptosis by alleviating ER stress in acute liver injury^21^. In combination, the regulation of ER stress could alleviate the pathological progression of liver injury. Thapsigargin (TG) and tunicamycin (TM) are known ER stress inducers that interrupt the intracellular calcium balance and inhibit protein glycosylation in the ER cavity, respectively^22^.

HNF1, which is a transcription factor enriched in the liver, is crucial for glucose metabolism, detoxification, and plasma protein synthesis^23^. Following acute inflammation, HNF1α participates in the regulation and repair of acute liver inflammation by promoting the expression of C-reactive protein^24^. To date, the impact of HNF1α on ER stress in liver injury remains unclear. Therefore, the impact of HNF1α on ER stress and apoptosis in human-derived hepatocytes and mice will be investigated.

## Results

### Significant apoptosis, HNF1α expression, and NF-κB activation in human hepatocytes during ER stress

**Figure 1:**
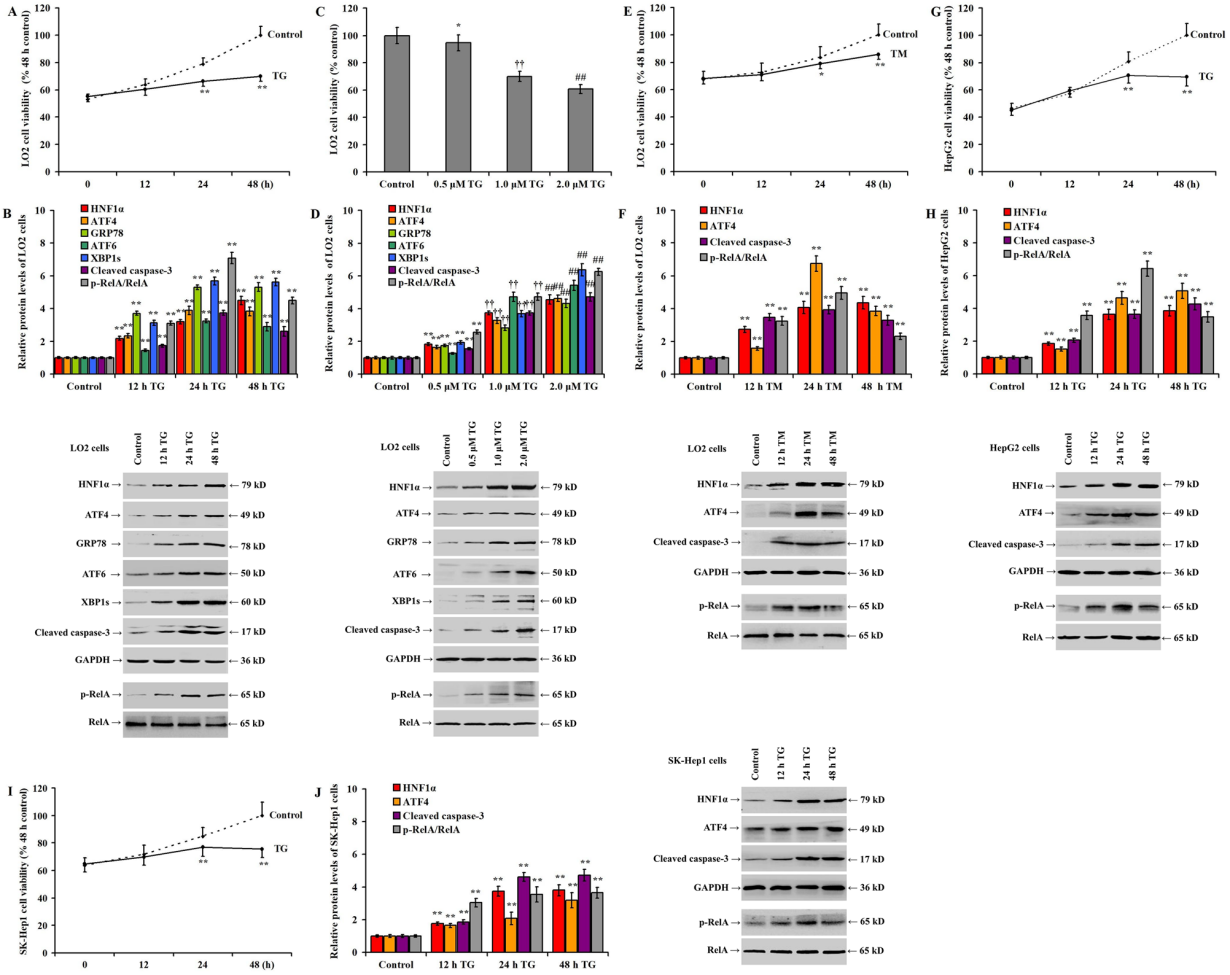
Significant apoptosis, HNF1α expression, and NF-κB activation in human-derived hepatocytes during ER stress. Human-derived hepatocytes were treated with TG in a time and dose-dependent manner and treated with dimethyl sulfoxide (DMSO) as a control: (**A**) LO2 cell viability as detected by MTS assay following treatment with TG (1.0 μmol/L) at different time points; (**B**) western blotting showing different protein expression and bar chart for relative protein expression following treatment with TG at different time points; (**C**) LO2 cell viability following ER stress induction by TG for 36 h in a dose-dependent manner; (**D**) western blotting showing the dose-dependent induced protein expression and bar chart for relative protein expression following TG treatment for 36 h; (**E**) cell viability in TM-induced LO2 cells; (**F**) western blotting and bar chart showing relative protein expression in TM-induced LO2 cells; (**G**) cell viability in TG-induced HepG2 cells; (**H**) western blotting and bar chart showing relative protein expression in TG-induced HepG2 cells; (**I**) cell viability in TG-induced SK-Hep1 cells; and (**J**) bar chart and western blotting showing relative protein expression in TG-induced SK-Hep1 cells. *p < 0.05, **p < 0.01 versus the control group. ^††^*p* < 0.01 versus the 0.5 μmoL/L TG group. ^##^*p* < 0.01 versus the 1.0 μmol/L TG group. μM: μmol/L.

In vitro, the addition of 1.0 μmol/L TG significantly decreased the viability of LO2 cells at 24 and 48 h (Fig. 1A, *p* < 0.05). In addition, it increased the expression of HNF1α, UPR signaling proteins (ATF4, GRP78, ATF6, and XBP1s), cleaved caspase-3, and RelA phosphorylation in LO2 cells at 12, 24, and 48 h (Fig. 1B, *p* < 0.05). Treatment of LO2 cells with different TG concentrations (0.5, 1.0, and 2.0 μmol/L) for 24 h revealed that the TG significantly reduced the viability of the LO2 cells in a dose-dependent manner (Fig. 1C). However, the expression of HNF1α, UPR signaling proteins (ATF4, GRP78, ATF6, and XBP1s), cleaved caspase-3, and RelA phosphorylation increased (Fig. 1D, *p* < 0.05). In addition, the treatment of LO2 cells with 1.0 μg/mL TM, another ER stress inducer, reduced LO2 viability (Fig. 1E, *p* < 0.05) and significantly upregulated HNF1α, ATF4, and cleaved caspase-3 expression and RelA phosphorylation (Fig. 1F, *p* < 0.05). Similarly, treatment of HepG2 cells with 1.0 μmol/L TG significantly decreased HepG2 viability (Fig. 1G, *p* < 0.05) and increased HNF1α, ATF4, and cleaved caspase-3 expression and RelA phosphorylation (Fig. 1H, *p* < 0.05). Similar results were observed in SK-Hep1 cells after treatment with 0.25 μmol/L TG (Fig. 1I and J, *p* < 0.05).

### Knockdown of HNF1A aggravates apoptosis and reduces ATF4 as well as GRP78 expression during ER stress in LO2 cells

**Figure 2:**
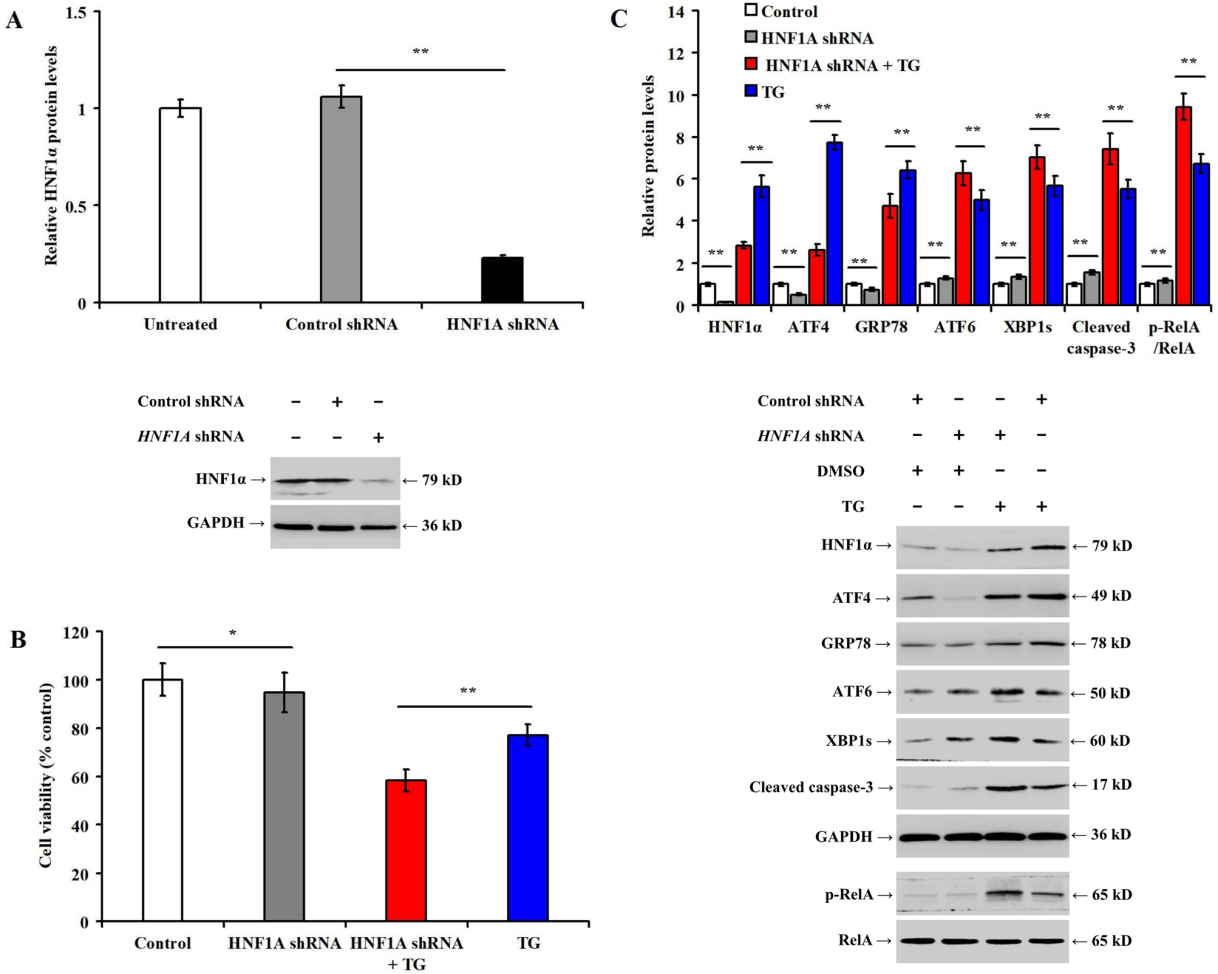
Knockdown of *HNF1A* aggravates apoptosis and reduces ATF4 and GRP78 expression during ER stress in LO2 cells. LO2 cells were pretreated with control shRNA or *HNF1A* shRNA for 48 h, then incubated with DMSO or TG (1.0 μmol/L) for 36 h: (**A**) bar chart and representative western blotting showing the alteration of HNF1α expression after *HNF1A* shRNA transfection; (**B**) bar chart representing MTS cell viability among the different experimental groups in LO2 cells; and (**C**) bar chart representing the relative protein expression and representative Western blotting for the different experimental groups. *p < 0.05, **p < 0.01, versus the control group (control shRNA, or control shRNA + DMSO) or TG group (control shRNA + TG).

To determine the role of HNF1α in hepatocyte apoptosis and ER stress in vitro, LO2 cells were transfected with *HNF1A* short hairpin RNA (shRNA) and related protein expression was analyzed 48 h later. Transfection of *HNF1A* shRNA significantly reduced the expression of HNF1α protein (Fig. 2A, *p* < 0.05). *HNF1A* shRNA reduced the viability of LO2 cells (Fig. 2B, *p* < 0.05), which was more obvious after 36-h TG treatment. In addition, the expression of HNF1α, ATF4, and GRP78 proteins decreased with or without TG (Fig. 2C, *p* < 0.05). However, it significantly increased the expression of ATF6, XBP1s, and cleaved caspase-3, and RelA phosphorylation.

A dual-luciferase reporter gene was used to detect the transcriptional regulation activity of HNF1α on *ATF4* promoter. Compared with the control group, HNF1α had an enhanced transcriptional regulation on either the wild-type *ATF4* promoter or mutant *ATF4* promoter (*p* < 0.01). Of interest, the transcriptional activity of HNF1α on wild-type *ATF4* promoter was stronger than that on mutant *ATF4* promoter (*p* < 0.01, Table 1).

**Table 1:** Relative luciferase activity (Ratio of Fluc/Rluc).

Transcription factor	Target promoter (human)	Hole 1	Hole 2	Hole 3	Hole 4	Hole 5	Hole 6	M ± SD
HNF1α	*ATF4*-promoter-wt	30.68	30.81	31.16	31.59	31.59	32.15	31.33 ± 0.56^a^^,^^c^
Control	*ATF4*-promoter-wt	18.63	18.77	19.02	19.09	19.16	19.40	19.01 ± 0.27
HNF1α	*ATF4*-promoter-mt	28.88	28.94	30.27	31.11	31.85	31.89	30.49 ± 1.36^b^
Control	ATF4-promoter-mt	19.71	19.97	20.05	20.08	20.80	20.88	20.25 ± 0.48

^a^*p* < 0.001, HNF1α versus the control group, *ATF4*-promoter-wt.

^b^*p* < 0.001, HNF1α versus the control group, *ATF4*-promoter-mt.

^c^*p* < 0.001, *ATF4*-promoter-wt versus ATF4-promoter-mt; mt: mutated type; wt: wild type.

### Knockdown of ATF4 aggravates apoptosis and downregulates the expression of GRP78 in TG-treated LO2 cells

**Figure 3:**
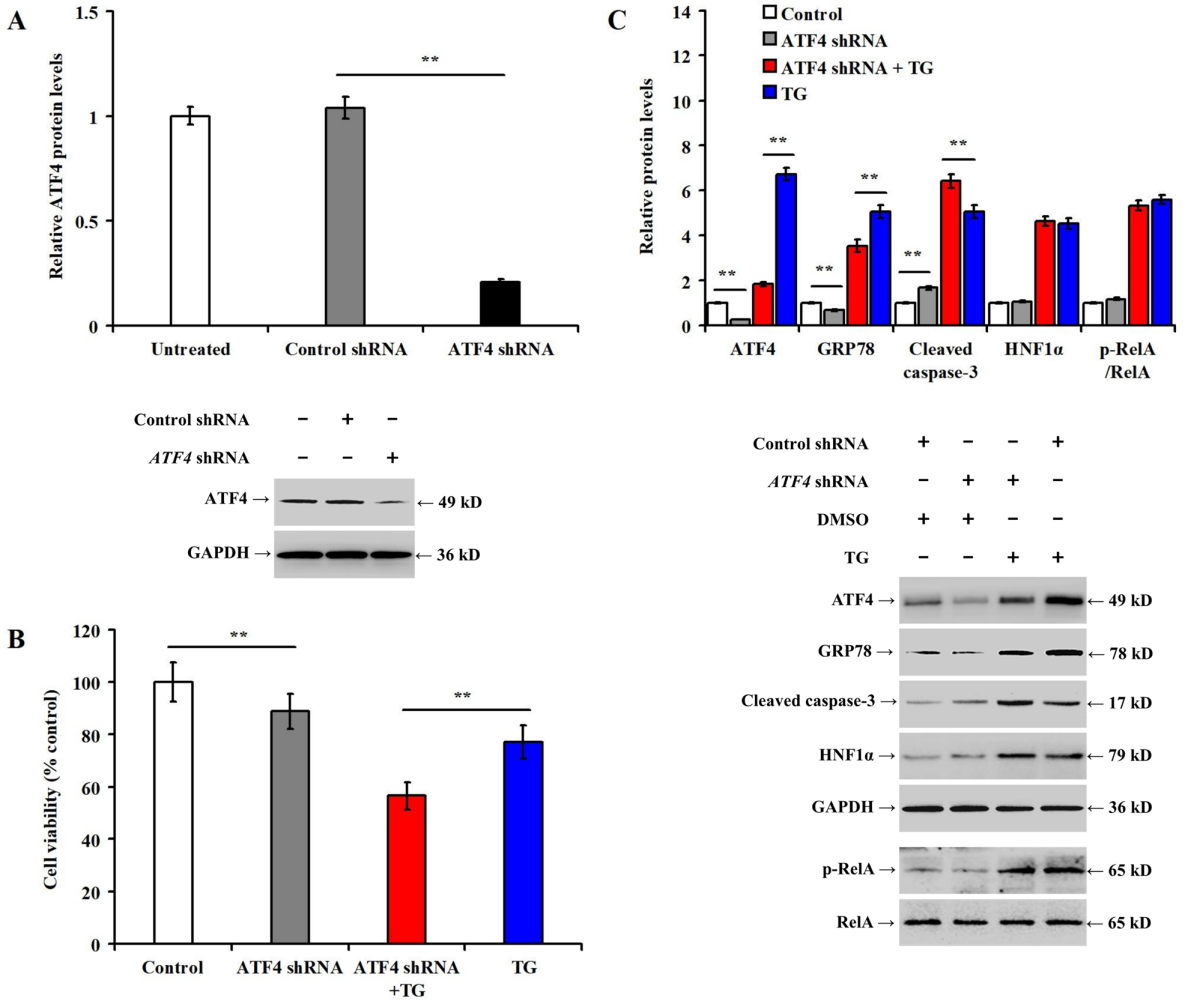
Knockdown of *ATF4* aggravates apoptosis and downregulates the expression of GRP78 in TG-treated LO2 cells. LO2 cells were pretreated with control shRNA or *ATF4* shRNA for 48 h and then incubated with DMSO or TG (1.0 μmol/L) for 36 h: (**A**) bar chart and representative Western blotting showing the alteration of ATF4 expression in different experimental; (**B**) bar chart representing LO2 cell viability between the different experimental groups; and (**C**) bar chart representing the relative protein expression and representative western blotting.**p < 0.01, versus the control (control shRNA, or control shRNA + DMSO) or TG groups (control shRNA + TG).

The downregulation of ATF4 protein was confirmed 48 h post-*ATF4* shRNA transfection in LO2 cells (Fig. 3A, *p* < 0.01). The knockdown of *ATF4* significantly reduced LO2 viability (Fig. 3B, *p* < 0.01), downregulated the expression of ATF4, GRP78 proteins, and increased the expression of cleaved caspase-3 in LO2 cells with or without TG (Fig. 3C, *p* < 0.01). However, knockdown of *ATF4* did not alter HNF1α expression and RelA phosphorylation.

### Knockdown of RELA inhibits HNF1α expression in TG-treated LO2 cells

**Figure 4:**
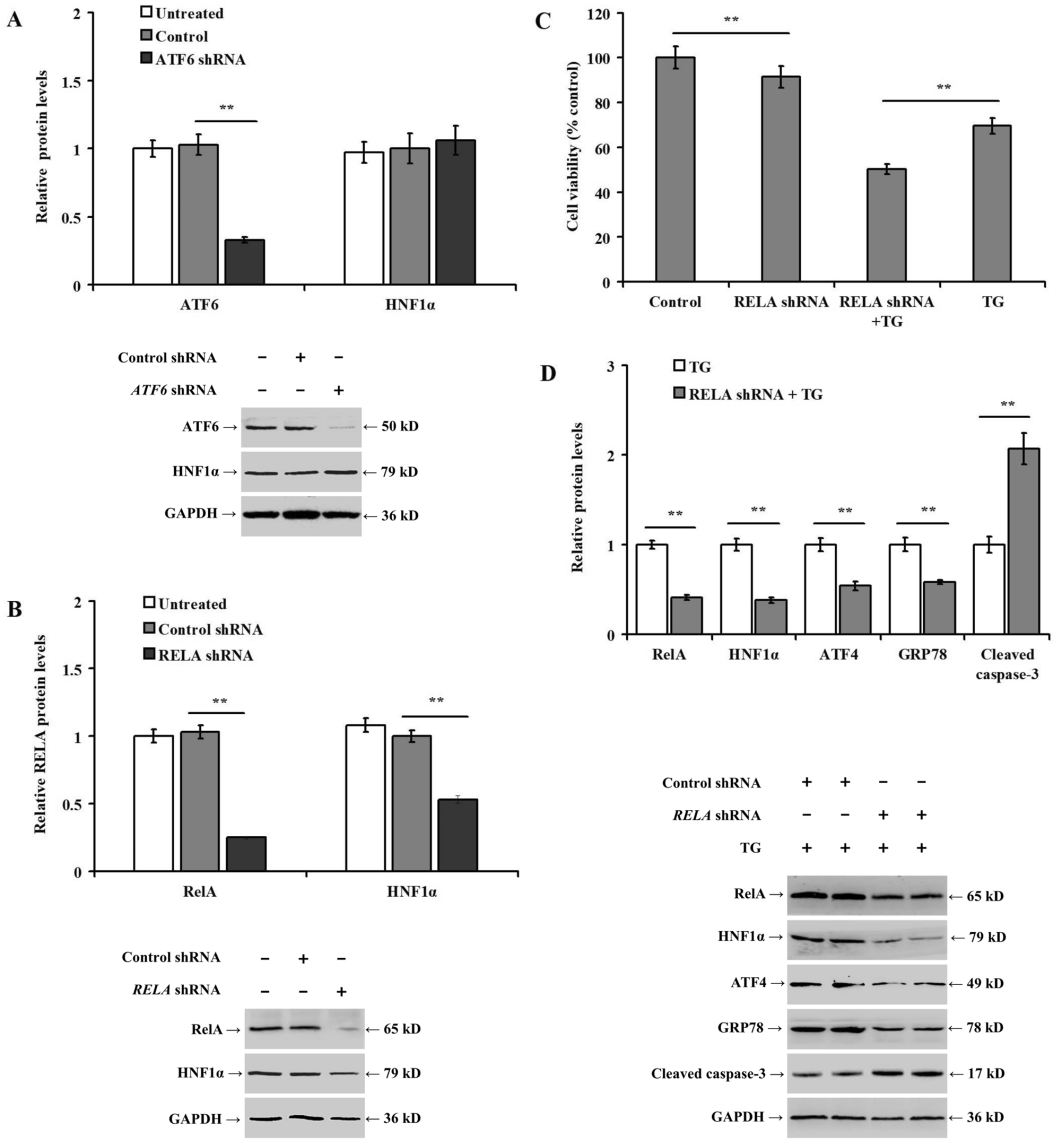
Knockdown of *RELA* inhibits TG-induced HNF1α expression in LO2 cells. LO2 cells were pretreated with control shRNA, *ATF6* shRNA, or *RELA* shRNA for 48 h, and then incubated with or without TG (1.0 μmol/L) for 36 h: (**A**) bar chart and representative western blotting showing the alteration in ATF6 and HNF1α expression after *ATF6* shRNA transfection in LO2 cells; (**B**) bar chart and representative Western blotting showing the alteration in RelA and HNF1α expression after *RELA* shRNA transfection in LO2 cells; (**C**) bar chart representing the impact of *RELA* knockdown on LO2 cell viability; and (**D**) bar chart representing the relative protein expression and representative western blotting between the different experimental groups. **p < 0.01, versus the control (control shRNA, or control shRNA + DMSO) or TG group (control shRNA + TG).

Knockdown of *ATF6* expression in LO2 cells by *ATF6* shRNA did not alter the expression of HNF1α protein (Fig. 4A, *p* > 0.05). However, knockdown of *RELA* reduced the expression of HNF1α protein (Fig. 4B, *p* < 0.01). Of note, knockdown of *RELA* reduced the viability of LO2 cells with or without TG treatment (Fig. 4C, *p* < 0.01), and decreased TG-induced the expression of HNF1α, ATF4, and GRP78 proteins, but increased the expression of cleaved caspase-3 (Fig. 4D, *p* < 0.01). In addition, bioinformatic analysis using JASPAR (http://jaspar.genereg.net/) predicted the presence of fifteen binding sites in *HNF1A* promoter for human *RELA*, based on the relative profile score of ≥ 80% (Table 2).

**Table 2:** Prediction of human-RelA binding to human-*HNF1A* promoter.

Relative score	Start	End	Strand	Predicted sequence
0.856078006	1989	1998	+	gagaatttcc
0.855413237	909	918	−	tggggtttca
0.853973996	201	210	−	ggggttttac
0.853973996	569	578	+	ggggttttgc
0.849202166	641	650	+	ttggcattcc
0.848738583	200	209	−	gggttttacc
0.847673958	1614	1623	−	gggcccttcc
0.826686199	1990	1999	−	gggaaattct
0.81884839	771	780	−	gggtcttgcc
0.808179668	1988	1997	+	ggagaatttc
0.806331988	123	132	−	tgggattaca
0.806331988	830	839	−	tgggattaca
0.806331988	967	976	−	tgggattaca
0.805502445	1991	2000	−	ggggaaattc
0.805138563	480	489	+	tgttttttcc

### HNF1α expression, ER stress, and apoptosis are associated with the severity of liver injury in CCl_4_-induced mice

**Figure 5:**
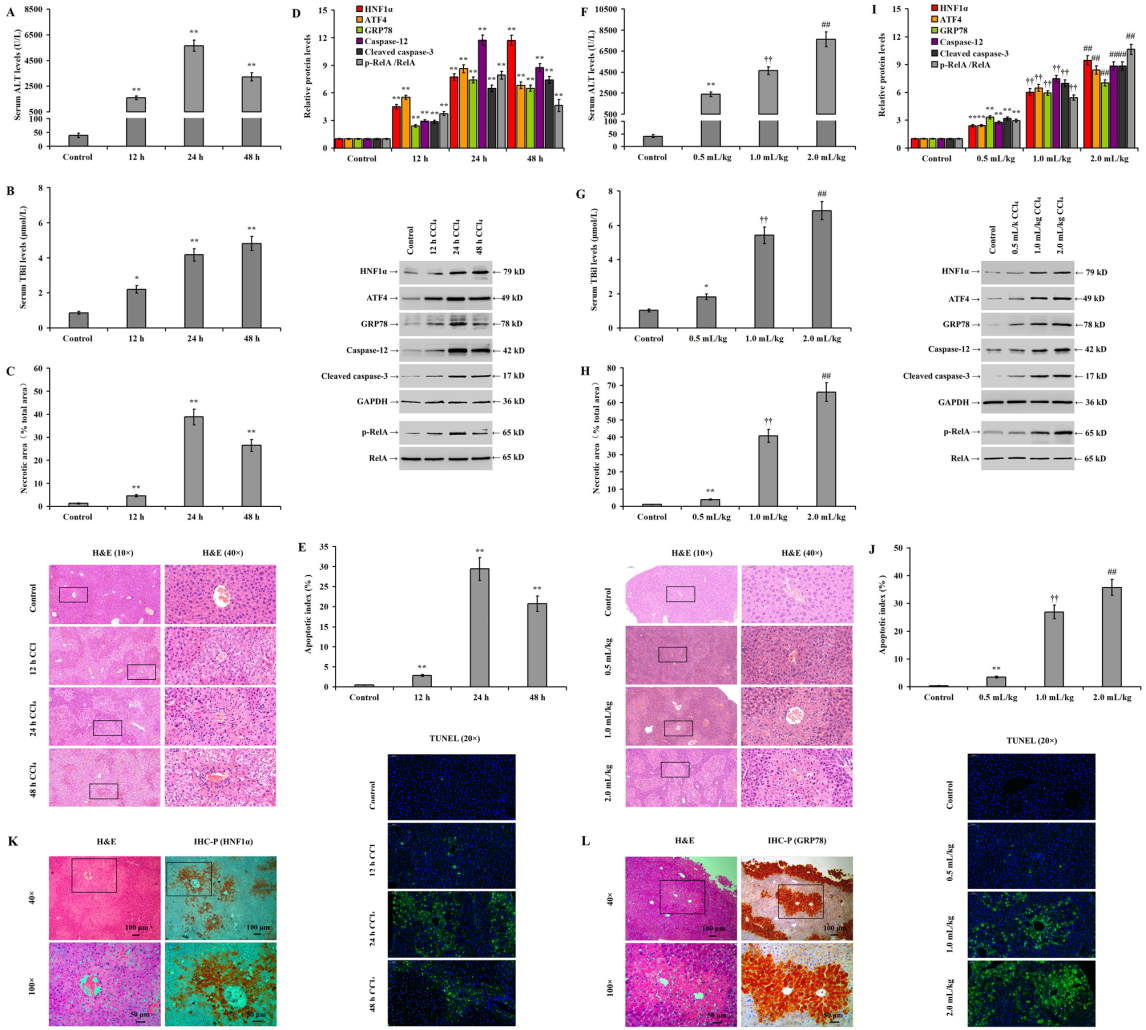
Expression of HNF1α, ER stress, and apoptosis signaling in CCl_4_-induced mice: (**A**) enzymatic rate method was used to detect the time-dependent changes in serum ALT levels in a CCl_4_-induced liver injury mouse model: (**B**) diazonium method to detect the time-dependent changes in serum TBil levels in mice; (**C**) bar charts representing the proportion of necrotic liver tissue area and H&E staining representing the pathological changes in the liver; (**D**) Western blotting reflecting the time-dependent changes in protein expression in CCl_4_-induced liver injury mouse model; (**E**) hepatocyte apoptotic index as measured by TUNEL staining; (**F**) dose-dependent changes in serum ALT levels detected by the enzymatic rate method; (**G**) dose-dependent changes in serum TBil levels detected by the diazonium method; (**H**) bar charts representing the proportion of necrotic liver tissue area and H&E staining representing pathological changes in the liver; (**I**) bar chart representing the relative protein expression levels of each protein; (**J**) bar charts representing the proportion of hepatocyte apoptotic index and TUNEL staining representing apoptotic changes in liver tissue; (**K**) immunohistochemical staining representing HNF1**α** expression; and (**L**) GEP78 expression. *p < 0.05, **p < 0.01 versus the control group. ^††^*p* < 0.01 versus the 0.5 mL/kg CCl_4_ group. ^##^*p* < 0.01 versus the 1.0 mL/kg CCl_4_ group. IHC-P: immunohistochemistry-paraffin.

Compared with the control group (olive oil), mice that were injected with 1.0 mL/kg carbon tetrachloride (CCl_4_) had significantly increased levels of serum alanine aminotransferase (ALT) and total bilirubin (TBil) at different time points (12, 24, and 48 h; Fig. 5A and B, *p* < 0.01). The area of necrotic tissue in the liver increased significantly at 12, 24, and 48 h (Fig. 5C, *p* < 0.01). Compared with the control group, HNF1α expression, protein levels in the UPR pathway (ATF4, GRP78, and caspase-12), cleaved caspase-3 expression, and the phosphorylation of RelA significantly increased at 12, 24, and 48 h after CCl_4_ injection (Fig. 5D, *p* < 0.01). The peak expression of HNF1α was observed at 48 h, and the peak expression of proteins involved in the ER stress pathway was observed 24 h after CCl_4_ injection. Similarly, the apoptotic index was significantly elevated at 12, 24, and 48 h after CCl_4_ injection, peaking at 24 h (Fig. 5E, *p* < 0.01).

Then, the impact of different CCl_4_ doses (0.5, 1.0, and 2.0 mL/kg CCl_4_) was analyzed at 24 h post-CCl_4_ injection. Compared with the control group, serum ALT and TBil levels as well as the necrotic tissue area significantly increased in a dose-dependent manner (Fig. 5F–H, respectively; *p* < 0.05). The protein expression of HNF1α, UPR signaling (ATF4, GRP78, ATF6, XBP1s, and caspase-12), cleaved caspase-3, and RelA phosphorylation increased in a dose-dependent manner in liver tissue (Fig. 5I, *p* < 0.01). Similarly, the apoptotic index was significantly elevated after CCl_4_ injection (Fig. 5J, *p* < 0.05). The expression of HNF1α and GRP78 was significantly upregulated in the injured liver tissue (Fig. 5K and L).

### Knockdown of Hnf1a aggravates CCl_4_-induced liver injury, hepatocyte apoptosis and ER stress in mice

**Figure 6:**
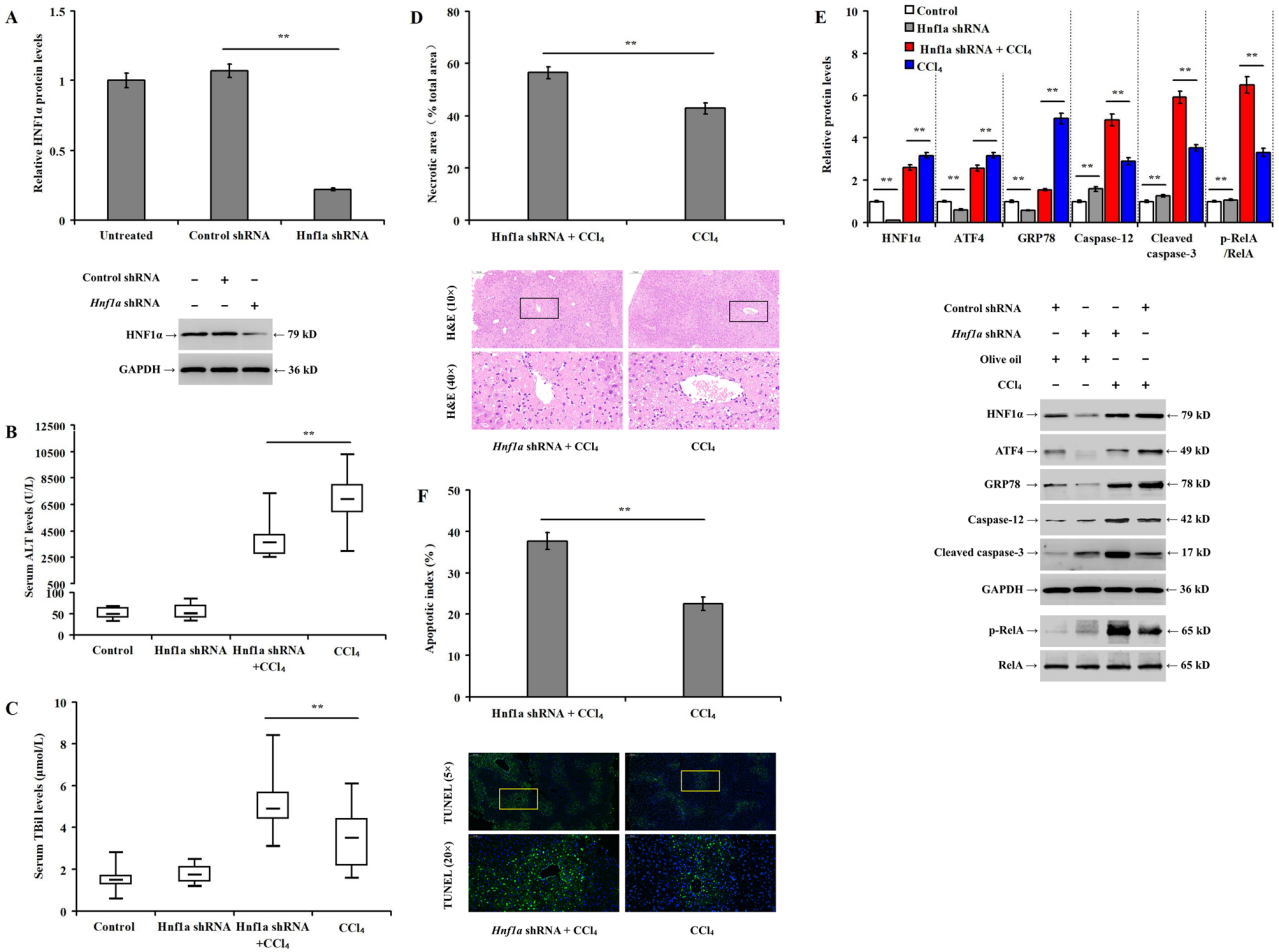
Knockdown of *Hnf1a* aggravates CCl_4_-induced liver injury, hepatocyte apoptosis, and ER stress in mice: (**A**) bar chart and western blotting showing the alteration in HNF1α expression in the untreated, or treated with control shRNA, or *Hnf1a* shRNA groups; (**B**) impact of *Hnf1a* knockdown on serum ALT levels in mice model of liver injury induced by CCl_4_; (**C**) impact of *Hnf1a* knockdown on serum TBil levels; (**D**) bar chart showing the proportion of necrotic liver tissue area and histopathological changes in mouse liver in the different experimental groups; (**E**) bar chart representing the relative protein expression and representative western blotting for the different experimental groups; and (**F**) hepatocyte apoptotic index measured by TUNEL staining. **p < 0.01 versus control (control shRNA, or control shRNA + olive oil) or CCl_4_ group (control shRNA + CCl_4_).

Mice were transfected with *Hnf1a* shRNA and rAAV8 vectors. Western blotting demonstrated the downregulation of HNF1α protein in mice liver 6 weeks after the transfection time (Fig. 6A, *p* < 0.01). Compared with the CCl_4_ group (control shRNA + CCl_4_), *Hnf1a* downregulation resulted in a significant reduction in the serum ALT level (Fig. 6B, *p* < 0.01), increased the serum TBil level (Fig. 6C, *p* < 0.01) and the necrotic liver tissue area (Fig. 6D, *p* < 0.01) in *Hnf1a* shRNA + CCl_4_ group. In addition, *Hnf1a* knockdown before CCl_4_ injection decreased the expression of HNF1α, ATF4, and GRP78, but significantly increased the expression of caspase-12, cleaved caspase-3, and p-RelA (Fig. 6E, *p* < 0.01), as well as the elevated apoptotic index (Fig. 6F, *p* < 0.01).

### LPS induces liver injury, hepatocyte apoptosis, ER stress and reduces HNF1α expression in mice

**Figure 7:**
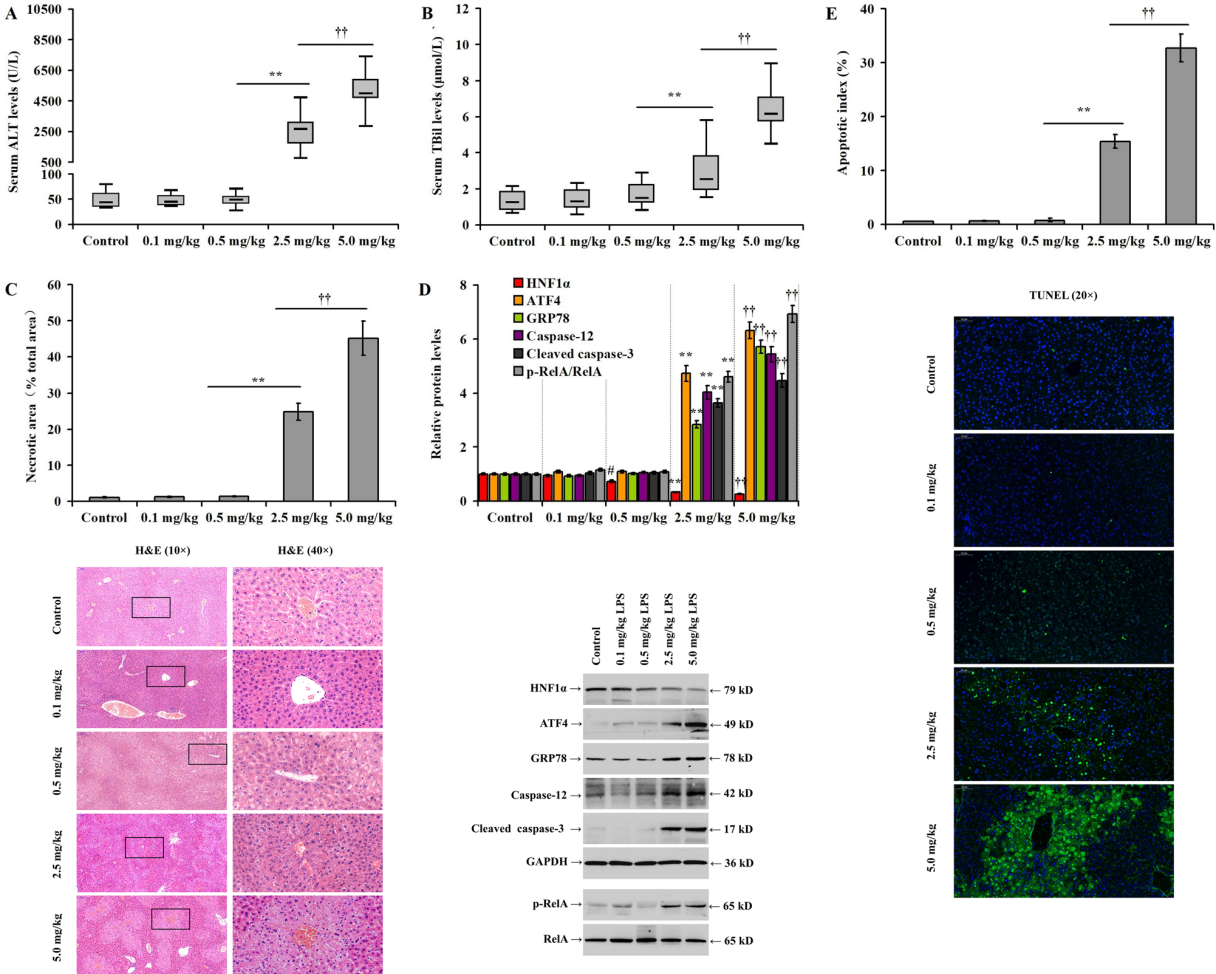
LPS induces liver injury, hepatocyte apoptosis and ER stress and reduces HNF1α expression in mice: (**A**) chart representing the impact of different LPS doses on the level of serum ALT in mice; (**B**) bar chart representing the impact of different LPS doses on the level of serum TBil in mice; (**C**) histopathological changes in mice liver in the different experimental groups; (**D**) bar chart and representative western blotting detecting the impact of different LPS doses on protein expression between the different experimental groups; and (**E**) hepatocyte apoptotic index measured by TUNEL staining. ^#^*p* < 0.05 versus the control group. **p < 0.01 versus the 0.5 mg/kg group. ^††^*p* < 0.01 versus the 2.5 mg/kg LPS group.

The dose-dependent impact of the intraperitoneal administration of lipopolysaccharide (LPS; 0.1, 0.5, 2.5, or 5.0 mg/kg for 24 h) on hepatic injury was examined. The low dosage of LPS (0.1 and 0.5 mg/kg) did not significantly alter the serum levels of ALT and TBil (Fig. 7A and B, *p* > 0.05), the necrotic liver tissue area (Fig. 7C, *p* > 0.05), the expression of ATF4, GRP78, caspase-12, cleaved caspase-3, and p-RelA (Fig. 7D, *p* > 0.05), and the apoptotic index (Fig. 7E, *p* > 0.05) in mice. However, 2.5 and 5.0 mg/kg LPS significantly increased the serum levels of ALT and TBil post-injection (Fig. 7A and B, *p* < 0.01). In addition, the necrotic liver tissue area significantly increased in the 2.5 or 5.0 mg/kg LPS (Fig. 7C, *p* < 0.01). Similarly, the same LPS doses increased the expression of ATF4, GRP78, caspase-12, cleaved caspase-3, and RelA phosphorylation (Fig. 7D, *p* < 0.01), and the apoptotic index (Fig. 7E, *p* < 0.01), but decreased the expression of HNF1α in the liver.

### Low dose LPS reduces CCl_4_-induced HNF1α expression and increases liver injury as well as hepatocyte apoptosis in mouse liver

**Figure 8:**
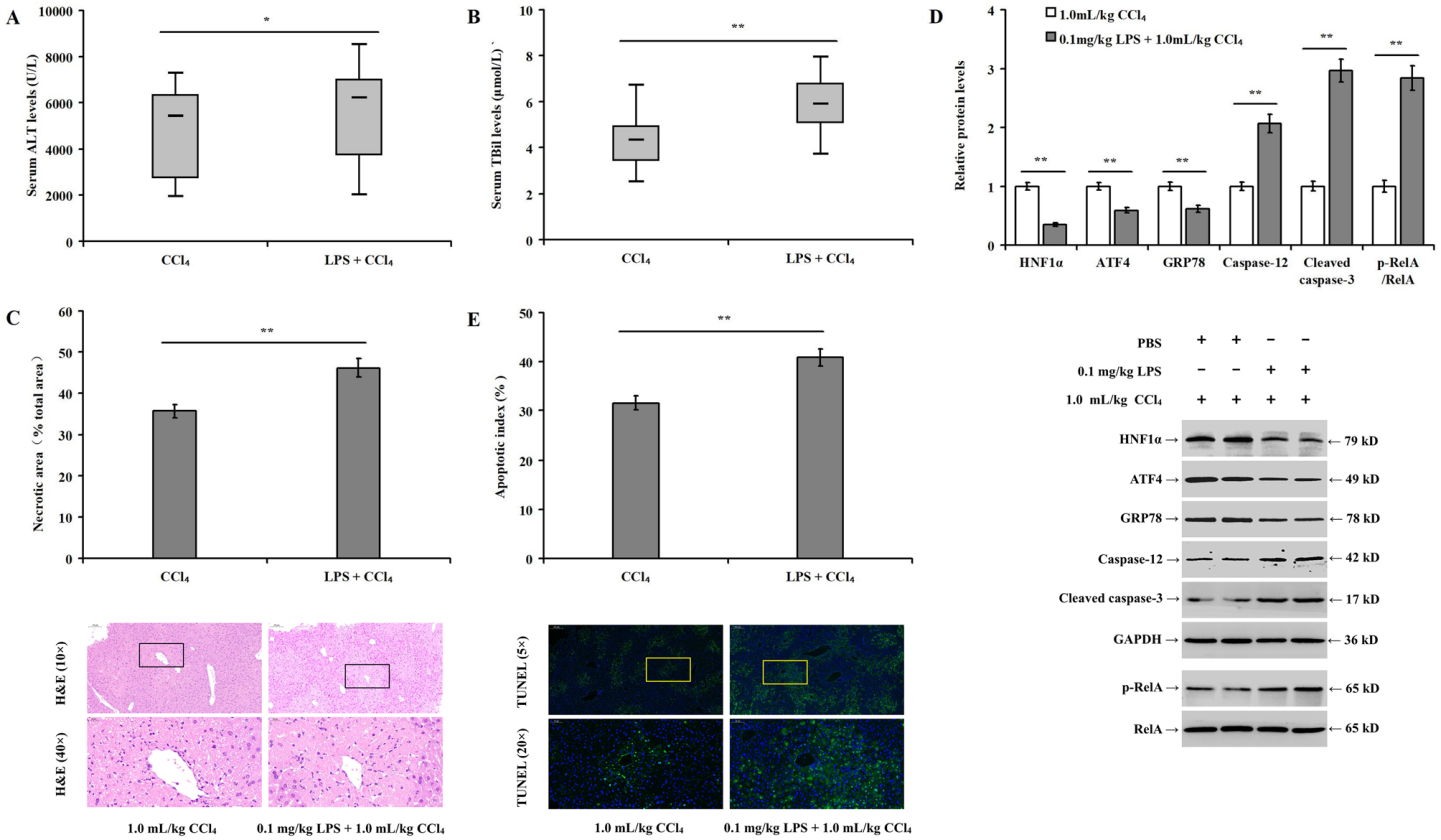
Low-dose LPS reduces CCl_4_-induced HNF1α expression and aggravates hepatocyte apoptosis in mouse liver: (**A**) effect of 0.1 mg/kg LPS on the level of serum ALT in a mouse model of liver injury induced by 1.0 mL/kg CCl_4_; (**B**) effect of 0.1 mg/kg LPS on the level of serum TBil; (**C**) histopathological changes in mice livers in the different experimental groups; (**D**) bar chart and western blotting detecting the impact of 0.1 mg/kg LPS on the expression of relative proteins; and (**E**) hepatocyte apoptotic index measured by TUNEL staining. *p < 0.05, **p < 0.01 versus the CCl_4_ group.

Compared with the CCl_4_ group, the addition of LPS induced a slight increase in the serum ALT level (Fig. 8A, *p* < 0.05), a significant increase in the serum TBil level (Fig. 8B, *p* < 0.01), and the necrotic liver tissue area (Fig. 8C, *p* < 0.01) in the LPS + CCl_4_ group. This combination significantly downregulated the expression of HNF1α, ATF4, and GRP78 increased the expression of caspase-12 and cleaved caspase-3, the phosphorylation of RelA (Fig. 8D, *p* < 0.01), and the apoptotic index in model mice (Fig. 8E, *p* < 0.01).

## Discussion

In this study, we examined the regulatory mechanism of HNF1α and ER stress and their impact on apoptosis in SAE of liver injury. Our results show that ER stress inducer TG or TM treatment induced apoptosis, and increased HNF1α expression in LO2, HepG2, and SK-Hep1 cells. Similarly, the upregulation of HNF1α expression, ER stress, and hepatocyte apoptosis were observed in the liver injury mouse model induced by CCl_4_. In addition, knockdown of RelA significantly inhibited the upregulation of HNF1α in vitro. Taken together, these results suggest that ER stress enhanced HNF1α expression in liver injury and might be involved in activating NF-κB signaling. Furthermore, the downregulation of HNF1α in vitro showed that apoptosis was aggravated. Of interest, the proteins involved in ER stress signaling were differentially expressed: the expression of ATF4 and GRP78 was significantly downregulated while ATF6 and XBP1 expression was upregulated. Similar results were observed in vivo*,* as well as enhanced ER stress-related apoptosis. In addition, the double fluorescent reporter gene assay confirmed that HNF1α regulated the transcription of *ATF4* promoter; knockdown of ATF4 decreased GRP78 expression and aggravated apoptosis in TG-treated LO2 cells. This interesting result implied potential crosstalk between HNF1α and ER stress through a feedback loop to alleviate hepatocyte apoptosis in liver injury.

Spontaneous peritonitis aggravated liver injury by LPS was partially simulated. The results demonstrated that LPS induced liver injury, hepatocyte apoptosis, and ER stress, but inhibited the expression of HNF1α in a dose-dependent manner. Treatment with low dosage LPS (0.1 mg/kg) selectively decreased CCl_4_-induced HNF1α, ATF4, and GRP78 expression and further aggravated ER stress-related hepatocyte apoptosis and liver injury. The ability of low-dosage LPS in inducing apoptosis and hence promoting the SAE of liver injury could be possibly attributed to the interference with the feedback loop between HNF1α and ER stress.

Hepatocyte degeneration and necrosis are fundamental in the pathogenesis of various liver diseases. This can cause the leakage or release of ALT, and irregular bilirubin metabolism in hepatocytes^25, 26^. In this study, liver injury was induced by CCl_4_ or LPS. Liver injury was evaluated by the levels of serum ALT and TBil, and the necrotic area of the liver tissue. In the liver, CCl_4_ is converted into carbon trichloride that causes oxidative stress, inflammation, and ER stress^27, 28^. LPS causes liver injury by activating inflammatory pathways or directly damaging hepatocytes^29^. In this study, CCl_4_ and LPS increased serum ALT and TBil levels, as well as the area of liver tissue necrosis, in a dose-dependent manner. This suggests the successful establishment of different severity degrees of liver injury models.

Hepatocyte apoptosis is closely related to liver injury^30^. Caspase plays a crucial role in apoptosis signal transduction^31^. Caspase-12 is related to ER-stress-mediated apoptosis^32^. Degradation of DNA into fragments of about 180–200 bp is a prominent morphological change marking apoptosis. In this study, hepatocyte apoptosis was comprehensively evaluated by analyzing the protein expression of caspase-12 and cleaved caspase-3. The apoptosis index was measured by TUNEL staining. Our results demonstrated that hepatocyte apoptosis significantly increased in CCl_4_-induced liver injury. Aggravation of liver injury by LPS increased hepatocyte apoptosis. This agreed with previous reports, where hepatocyte apoptosis was associated with the severity of liver injury^33^. Therefore, enhancing the resistance of hepatocytes towards apoptosis might be a feasible strategy to prevent liver injury.

ER stress promotes intracellular homeostasis through UPR response, but excessive ER stress activates apoptosis signaling pathways^34^. Simultaneously, ER stress inhibits the overall protein synthesis through PERK/eIF2α signaling and upregulates the expression of molecular chaperones, such as GRP78 through ER stress-related transcription factors, such as ATF6, ATF4, and XBP1^21^. In this study, the expression of ATF4, ATF6, XBP1s, GRP78, and caspase-12 were analyzed to monitor ER stress. Results demonstrated that ER stress was associated with hepatocyte apoptosis and liver injury that was induced by CCl_4_ or LPS. In addition, TG or TM induced ER stress and apoptosis in LO2, HepG2, and SK-Hep1 cells. Accumulating evidence suggests that targeted regulation of ER stress may change the progression of liver injury by altering hepatocyte apoptosis^35^.

HNF1α is a transcription regulator that is essential for normal liver function^36^. In rats, downregulating HNF1α promoted the development of liver fibrosis and the overexpression of HNF1α significantly reduced liver fibrosis in rats^37^. Following acute inflammation, HNF1α regulates the repair of acute liver inflammation by promoting C-reactive protein expression^38, 39^. In this study, it was demonstrated that HNF1α expression increased in TG-induced ER stress. Similarly, the upregulation of HNF1α expression in CCl_4_-induced liver injury in mice and HNF1α expression was positively correlated with the severity of liver injury. Immunohistochemistry indicated elevated HNF1α expression and ER stress. These results imply that ER stress can induce HNF1α expression, as well as hepatocyte apoptosis, in liver injury both in vivo and in vitro*.*

In ER stress, IRE1, PERK/eIF2α/ATF4, and ATF6 signaling are activated which promotes the downstream signaling cascades^19^. ATF4 plays an essential role in stress signaling, which includes ER stress, hypoxia, amino acid deletion, and oxidative stress^40^. In particular, ATF4 is involved in the transcriptional regulation of amino acid synthesis, protein folding and degradation, redox balance, autophagy, and apoptosis^39, 41, 42^. Mutations in ATF4 significantly altered glucose homeostasis and energy consumption^43^ .The activation of ATF6 upregulates the expression of ER stress-related proteins, which include XBP1 and GRP78, and enhances the ability of cells to eliminate misfolded proteins^44, 45^. NF-κB signaling refers to a family of nuclear transcription factors, which includes RelA (NF-κB p65), RelB, c-Rel, NF-κB1/p50, and NF-κB2/p52^9^. In this study, ATF4, ATF6 and RelA were knocked down to analyze the impact of ER stress on HNF1α. The knockdown of RelA reduced the expression of HNF1α in TG-treated LO2 cells; however, ATF4 and ATF6 knockdown did not downregulate HNF1α expression. These results suggest that the activation of NF-κB might be one of the ways by which ER stress mediates the upregulation of HNF1α.

However, to the best of our knowledge, the regulatory role of HNF1α in ER stress and apoptosis in hepatocytes is unknown. In this study, the knockdown of HNF1α increased TG-induced apoptosis in vivo*.* On the other hand, HNF1α knockdown aggravated the CCl_4_-induced liver damage as indicated by increased TBil levels, apoptosis, and necrotic liver area. Of note, serum ALT levels did not increase, which might be attributed to the regulatory role of HNF1α on numerous liver-specific genes^46^. ALT is a metabolic enzyme; therefore, knocking down HNF1α might decrease liver ALT levels and decrease serum ALT These results suggest that HNF1α upregulation mitigates liver injury by reducing hepatocyte apoptosis. Further, knockdown of HNF1α differentially affects the expression of ER stress-related proteins. Specifically, it significantly downregulated the expression of ATF4 and GRP78, but increased ATF6, XBP1s, and caspase-12 expression in vivo and in vitro*.* In addition, this confirmed that HNF1α regulated the transcription of *ATF4* promoter. These results suggest that HNF1α may reduce hepatocyte apoptosis through mitigating ER stress during acute phase liver injury.

The knockdown of ATF4 reduced GRP78 levels and increased apoptosis in TG-treated LO2 cells. This suggested that ATF4-mediated GRP78 expression in ER stress is beneficial to alleviate apoptosis, which agrees with previous research^47–50^. These results suggest that HNF1α may reduce ER stress-mediated hepatocyte apoptosis through upregulating the expression of ATF4 and GRP78.

Viral and bacterial infections are common risk factors that are associated with severe liver injury^51, 52^. Endotoxins, which are the main toxic effect component is LPS, are a cell wall component of Gram-negative bacteria. Under normal physiological conditions, a small amount of LPS produced by Gram-negative bacteria in the intestine can reach the liver via the portal circulation^53^. Most LPS is cleared by Kupffer cells without damaging hepatocytes. Compromised intestinal barrier function increases LPS leakage and this eventually leads to hepatocyte damage^54^. LPS causes liver inflammation, hepatocyte apoptosis, and aggravates liver cell injury^55, 56^. Blood endotoxin levels are positively correlated with the degree of hepatocyte apoptosis^57, 58^. In this study, the effect of spontaneous peritonitis aggravated liver injury by LPS injection was partially simulated. Our results demonstrated that LPS dose-dependently induced liver injury, hepatocyte apoptosis, and ER stress, but inhibited the expression of HNF1α during liver injury. In addition, low-dose LPS did not cause liver damage, and its preintervention could alleviate the liver injury^59^. However, low-dose LPS aggravates the liver injury, hepatocyte apoptosis and ER stress, inhibits the mutual regulation of HNF1α and ATF4, and reduces GRP78 expression in the CCl_4_-induced liver injury model. Therefore, LPS treatment can have different outcomes depending on the baseline status of the liver. Under physiological conditions, low-dose LPS is not enough to cause liver damage but it significantly increases the hepatocyte sensitivity towards apoptotic signaling mediated by ER stress after liver damage has occurred. Intestinal endotoxemia is common in severe liver injury^60^. However, the role of low-level LPS in promoting the SAE of the liver in future studies needs to be determined.

It has been demonstrated that HNF1α expression was not correlated with chronic liver failure^61^. The results of this study agree with previous research and support the existence of multiple regulatory modes of HNF1α in liver injury. The expression level of HNF1α might not correlate with the degree of injury, but the effective upregulation of HNF1α in liver injury could be beneficial in controlling liver injury.

**Figure 9:**
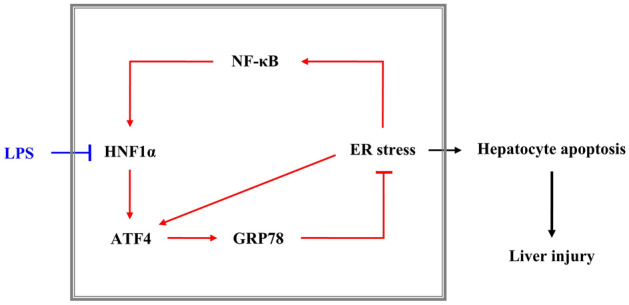
Schematic diagram representing the mechanism of suggested hepatocyte HNF1α–ER stress feedback loop on apoptosis in acute liver injury. Following liver injury, ER stress upregulates the expression of HNF1α by activating NF-κB signaling; the upregulated HNF1α reduces ER stress through positive feedback regulation with ATF4, which results in upregulation of GRP78 expression and reduction of hepatocyte apoptosis and liver injury. Low-dose LPS aggravates the CCl_4_ induced hepatocyte apoptosis and liver injury by inhibiting the mutual regulation of HNF1α and ER stress.

In conclusion, following liver injury, hepatocyte HNF1α and ER stress are mutually regulated to form a feedback loop, which can help reduce the severity of liver injury by regulating hepatocyte apoptosis. Whereas ER stress upregulates the expression of HNF1α by activating NF-κB signaling and the upregulated HNF1α reduces ER stress through selective regulation of ATF4 increasing GRP78 expression. In addition, high-dose LPS can directly cause liver injury, hepatocyte apoptosis as well as ER stress and inhibit the expression of HNF1α. Low-dose LPS alone cannot cause liver damage; however, it reduces the tolerance of hepatocytes to apoptosis in liver injury and leads to the deterioration of liver injury through interfering with the feedback regulation of HNF1α and ER stress (Fig. 9).

## Methods

### Cell culture and UPR induction

Human-derived hepatocytes LO2, HepG2, and SK-Hep1 cells were purchased from The Cell Bank of Type Culture Collection of Chinese Academy of Sciences (Shanghai, China) and were maintained in RPMI-1640 that was complemented with 10% fetal bovine serum and 1% antibiotics. To induce UPR, LO2, HepG2, or SK-Hep1 cells were treated for 12, 24, and 48 h with either dimethyl sulfoxide (DMSO; TG solvent), phosphate buffer saline (PBS; TM solvent), TG (Sigma), or TM (Sigma). Alternatively, LO2 cells were treated with different concentrations of TG (0.5, 1.0, and 2.0 μmol/L) and samples were analyzed after 24 h. To analyze the impact of HNF1α downregulation in vitro, LO2 cells were transfected with the target shRNA or control shRNA using a plasmid vector according to the manufacturer protocols and protein expression was analyzed 48 h later (Table 3). Similarly, we analyzed the impact of ATF4, ATF6 and RelA downregulation using their respective target shRNA (Table 3). Then, cells were treated with TG (1.0 μmol/L) for 36 h to induce hepatic ER stress. Previous studies demonstrated that SK-Hep1 cells were more sensitive to ER stress inducers; therefore, an induction dose of 0.25 μmol/L TG was used^62^.

**Table 3:** shRNA sequences used in LO2 cells.

Insert content	5' to 3'	
HNF1α	Target sequence	GCTAGTGGAGGAGTGCAATAG
	*HNF1A* shRNA	GCTAGTGGAGGAGTGCAATAGCGAA*CTATTGCACTCCTCCACTAGC*
	Control shRNA	AAACGTGACACGTTCGGAGAACGAATTCTCCGAACGTGTCACGTTT
ATF4	Target sequence	GGAGATCCAGTACCTGAAAGA
	*ATF4* shRNA	GGAGATCCAGTACCTGAAAGACGAA*TCTTTCAGGTACTGGATCTCC*
	Control shRNA	AAACGTGACACGTTCGGAGAACGAATTCTCCGAACGTGTCACGTTT
ATF6	Target sequence	GCAGGTCCTCCTGTTATTAGA
	*ATF6* shRNA	GCAGGTCCTCCTGTTATTAGACGAA*TCTAATAACAGGAGGACCTGC*
	Control shRNA	AAACGTGACACGTTCGGAGAACGAATTCTCCGAACGTGTCACGTTT
RelA	Target sequence	CACCATCAACTATGATGAGTT
	*RELA* shRNA	CACCATCAACTATGATGAGTTCGAA*AACTCATCATAGTTGATGGTG*
	Control shRNA	AAACGTGACACGTTCGGAGAACGAATTCTCCGAACGTGTCACGTTT

### Cell viability assay

The percentage of living cells was assessed using an MTS assay, [3-(4,5-dimethylthiazol-2-yl)-5-(3-carboxymethoxyphenyl)-2-(4-sulfophenyl)-2H-tetrazolium] assay (Promega, USA) according to the manufacturer protocol. Briefly, 100 μL of LO2, HepG2, or SK-Hep1 cells (10^4^–10^5^ cells/mL) were seeded in the inner wells of a 96 well plate. Each experimental condition was seeded in quadruplicate and wells around the edges were filled with sterile PBS to maintain humidity in the 96-well plate. The following day, cells underwent different interventions. At the end of the experiment, MTS assay solution was added to each well and incubated at 37 °C for 4 h. Finally, the colorimetric signal was observed at 490 nm on a microplate reader (Bio-Rad model 680; Bio-Rad, Hercules, CA, USA). The activity of the experimental control group was set to100%.

### Animals and induction of liver injury

A total of 168 male BALB/c mice (25.0 ± 3.0 g) were obtained from the Animal Center of Zunyi Medical University (Guizhou, China). Mice were maintained under temperature and humidity-controlled conditions (18–22 °C and 50–60%, respectively) with food and water available ad libitum. Following acclimatization, mice were randomly assigned to different experimental groups (untreated, control, model group, and combination group; n = 12). The experimental protocol was reviewed and approved by the Animal Experimental Ethics Committee, Zunyi Medical University (ZMC-LS [2020] No. 2-321) according to the animal care and research guidelines^63^. This study is reported in accordance with ARRIVE guidelines.

Acute liver injury was induced by a single intraperitoneal injection of CCl_4_ dissolved in olive oil (CCl_4_: olive oil ratio = 1:4). Mice in the CCl_4_ model group received 1.0 mL/kg CCl_4_ injection for 12, 24, or 48 h whereas mice in the control group received the same volume of olive oil. Otherwise, mice in the CCl_4_ model group received either 0.5, 1.0, or 2.0 mL/kg CCl_4_ injections and the control group mice received the same dose of olive oil. Mice were not allowed to consume food or water 6 h before the injection. Alternatively, acute liver injury was induced by LPS; whereas mice in the model group were given an intraperitoneal injection of LPS (0.1, 0.5, 2.5, and 5.0 mg/kg) dissolved in PBS for 24 h. Mice in the solvent control group received the same volume of PBS and mice in the normal control group were untreated. Mice in the combination group (LPS and CCl_4_) received LPS (0.1 mg/kg) and CCl_4_ (1.0 mL/kg) for 24 h. Mice were not allowed to consume food or water 6 h before injection. The degree of liver injury was evaluated by detecting biochemical liver function indices, and histopathological changes in liver tissues. Further, liver injury was monitored at 12, 24, and 48 h. Mice were sacrificed by CO_2_ euthanasia. Mice were placed in a 4000-mL euthanasia box and the box chamber air was replaced with 100% CO_2_ at a rate of 30% of chamber volume/min^64^. Following the loss of consciousness, tissue and blood were harvested when blood circulation was maintained.

### Knockdown of HNF1α in mice liver

Recombinant serotype 8 adeno-associated virus (rAAV8) that carried *Hnf1a* shRNA (target sequence: 5'-GCGATGAGCTGCCAACTAAGA-3'; Syngentech, Beijing, China; Table 4) was injected through the tail vein. Each mouse received an injection of 2 × 10^10^ viral genome copies that were dissolved in 0.1 mL PBS. Successful knockdown of *Hnf1a* was confirmed by Western blotting after 6 weeks of viral injection. Mice were randomly assigned to an untreated (normal control group; n = 12), control shRNA group (n = 12), and *Hnf1a* shRNA group (n = 12). Then, the role of HNF1α in liver injury was explored. Mice were randomly assigned to a control (control shRNA + olive oil; n = 12), *Hnf1a* shRNA (*Hnf1a* shRNA + olive oil; n = 12), CCl_4_ (control shRNA + CCl_4_; n = 12), *Hnf1a* shRNA + CCl_4_ group (n = 12). Mice in the control and *Hnf1a* shRNA groups received olive oil injections (same volume of CCl_4_ as previously); whereas mice in the control shRNA + CCl_4_ and *Hnf1a* shRNA + CCl_4_ groups received 1.0 mL/kg CCl_4_ injection for 36 h.

**Table 4:** Sequence of the control and *Hnf1a* shRNA.

Insert content	Sequence
*Hnf1a* shRNA	5'-GCGATGAGCTGCCAACTAAGA CGAA*TCTTAGTTGGCAGCTCATCGC*-3'
Control shRNA	5'-AAACGTGACACGTTCGGAGAA CGAATTCTCCGAACGTGTCACGTTT-3'

### Western blotting

LO2, HepG2, SK-Hep1 cells, or liver tissues were homogenized in immunoprecipitation assay lysis buffer (R0010, Solarbio, Beijing, China). Liver lysates (40 µg) were separated on a 10% sodium dodecyl sulfate–polyacrylamide gel and transferred to polyvinylidene fluoride membranes (Millipore, Billerica, MA, USA). Following blocking, membranes were probed with mouse monoclonal antibodies against ATF6 (sc-166659, 1:1000, Santa Cruz Biotechnology), eIF2α (sc-133132, 1:1000), GAPDH (glyceraldehyde-3-phosphate dehydrogenase; sc-365062, 1:1000), HNF1α (sc-393668, 1:1000), p-RelA (sc-166748, 1:1000), or RelA (sc-514451, 1:1000), rabbit monoclonal antibodies against ATF4 (11,815, 1:1000, Cell Signaling Technology), cleaved caspase-3 (9664, 1:1000, Cell Signaling Technology), GRP78 (ab108615, 1:10,000, abcam), XBP1s (40,435, 1:1000, Cell Signaling Technology), or p-eIF2α (3398, 1:1000, Cell Signaling Technology), or rabbit polyclonal antibody against caspase-12 (2202, 1:1000, Cell Signaling Technology), protein bands were detected with enhanced chemiluminescent and images were processed using Quantity One software (Bio-Rad, Hercules, CA, USA). Densitometric analysis was used to detect the level of each protein relative to the control.

### Histochemical and immunohistochemical analysis

Following fixation, liver tissues were dehydrated, paraffin-embedded and sliced at 4 μm thickness. Tissue samples were stained with hematoxylin–eosin (H&E) according to standard protocols and scanned on a sliced Panoramic scanner (Pannoramic DESK/MIDI/250/1000, 3DHISTECH, Hungary) and CaseViewer2.2 software (3DHISTECH, Hungary).

Otherwise, sections were immunostained using monoclonal antibodies against HNF1α (ab272708, 1:200, abcam) and GRP78 (ab108615, 1: 200, abcam). Then, sections were visualized under a light microscope (OLYMPUS CX31). Each H&E section was independently scored by two experienced pathologists using the Histology Activity Index–Knodell score as detailed previously^65^.

### TUNEL staining

Hepatocyte apoptosis was detected using a TUNEL kit (Roche, 11684817910). Deoxyribonucleotide terminal transferase (TdT) and deoxyribonucleotide derivative digoxigenin [(digoxigenin)-11-dUTP] were mixed at 2:28, added to the paraffin-embedded liver sections and incubated in a humidified chamber for 2 h according to standard protocols^66^. Positive staining was examined using a fluorescent microscope with 4′,6-diamidino-2-phenylindole (DAPI) counterstaining. The apoptotic index was calculated from six randomly selected fields according to the following formula: apoptotic index = number of positive cells/total number of cells × 100%.

### Liver function indexes

Serum ALT and TBil levels were measured using the rate method and diazonium, respectively according to the standard protocols (Beckman Coulter auto-analyzer, AU5800, USA)^19, 67^.

### Dual luciferase reporter assay

The search engine of the University of California, Santa Cruz Genomics Institute was used to find the promoter sequence of *ATF4* (https://genome.ucsc.edu/). The binding site of HNF1α to the *ATF4* promoter sequence was predicted through JASPAR (http://jaspar.genereg.net/). Then, the *ATF4* promoter sequence was synthesized. Next and cloning it on the firefly luciferase gene, constructing as an *ATF4*-promoter-wt plasmid. In addition, the HNF1α binding site on the *ATF4* promoter sequence was mutated to serve as the *ATF4*-promoter-mt plasmid. The nucleotide sequence that corresponded to *HNF1A* was cloned into the pcDNA3.1 vector. The pcDNA3.1 empty vector was used as a control. HEK 293FT cells (ATCC) were cultured and seeded into 24-well plates and grown for 10–24 h (80% confluence). Cells were cotransfected with the reporter gene plasmid, and the transcription factor expression plasmid, and the Renilla luciferase plasmid (an internal reference). Following cell lysis and protein extraction, luciferase activity was detected by firefly luciferase and Renilla luciferase detection reagents (Biyuntian Biotechnology, Shanghai, China) to determine the relative light unit (RLU). The RLU value (Fluc) obtained by the firefly luciferase assay was divided by the RLU (Rluc) value obtained by the Renilla luciferase assay as an internal reference. According to the obtained ratio, the activation degree of the target reporter gene between the different groups was compared^68^.

### Statistical analysis

One sample Kolmogorov-Smirnow test was used to test whether the continuous variables satisfied a normal distribution. Quantitative data that satisfied a normal distribution were shown as mean ± standard deviation ($\overline{\chi }$ ± SD). Differences between groups were evaluated using one-way ANOVA. If the difference was statistically significant, the Student–Newman–Keuls test was used for further comparison between the two groups. A *p*-value < 0.05 was statistically significant.

### Ethics approval

This study was reviewed and approved by the Animal Experimental Ethics Committee, Zunyi Medical University (ZMC-LS [2020] No. 2-321) according to the animal care and research guidelines. All procedures were performed following the relevant guidelines and regulations.

## Supplementary Information

Supplementary Information 1.

Supplementary Information 2.

## Data Availability

The datasets generated and analyzed during the current study are not publicly available due to none of the data types requiring uploading to a public repository but are available from the corresponding author on reasonable request.
