# Supplementary material for: Feedback loop between hepatocyte nuclear factor 1α and endoplasmic reticulum stress mitigates liver injury by downregulating hepatocyte apoptosis
Source: Sci Rep. 2022 Jul 8;12:11602. doi: 10.1038/s41598-022-15846-8 (PMC9270423; doi:10.1038/s41598-022-15846-8)

Figure 1B（LO2 cells）

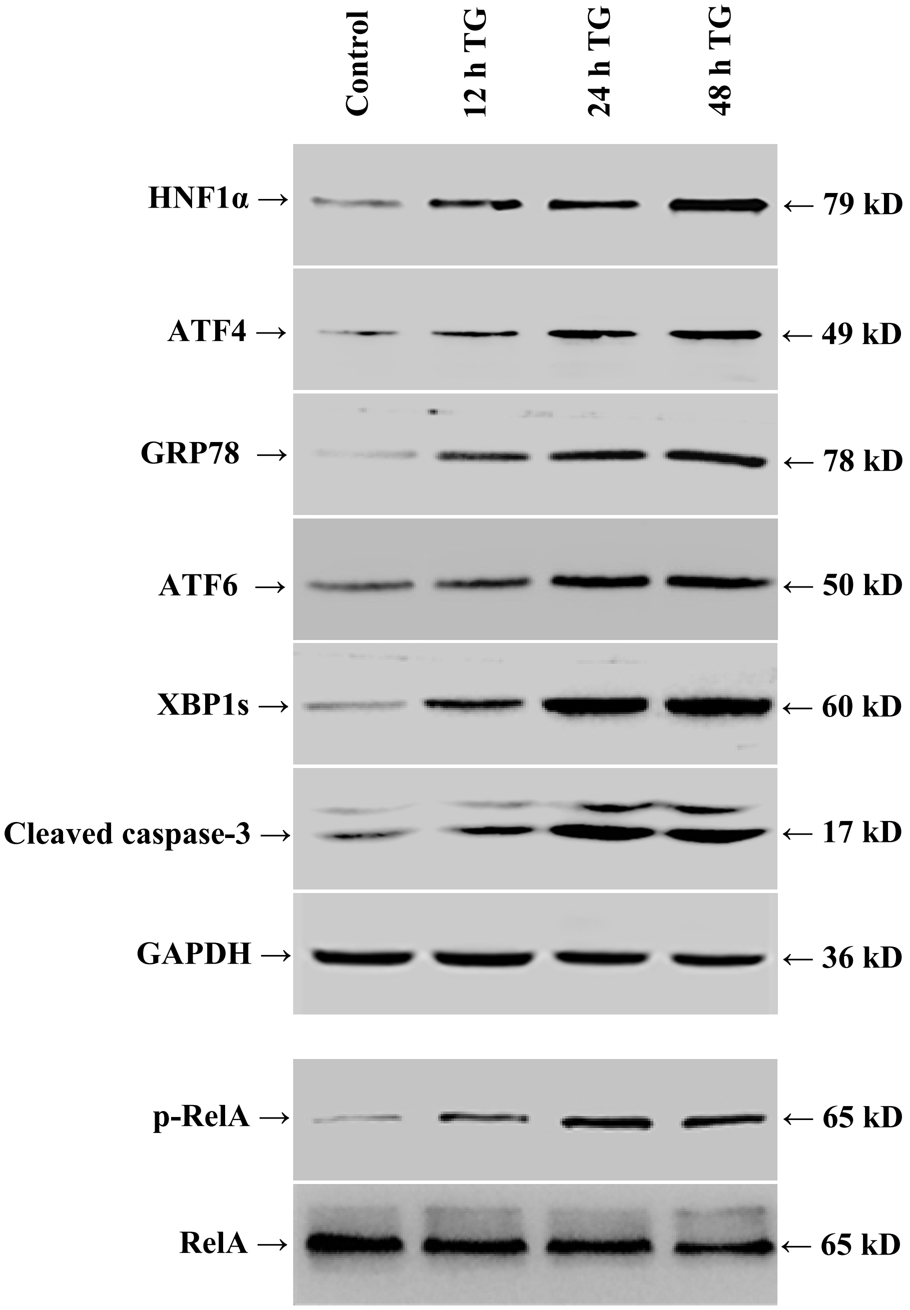

Figure 1D（LO2 cells）

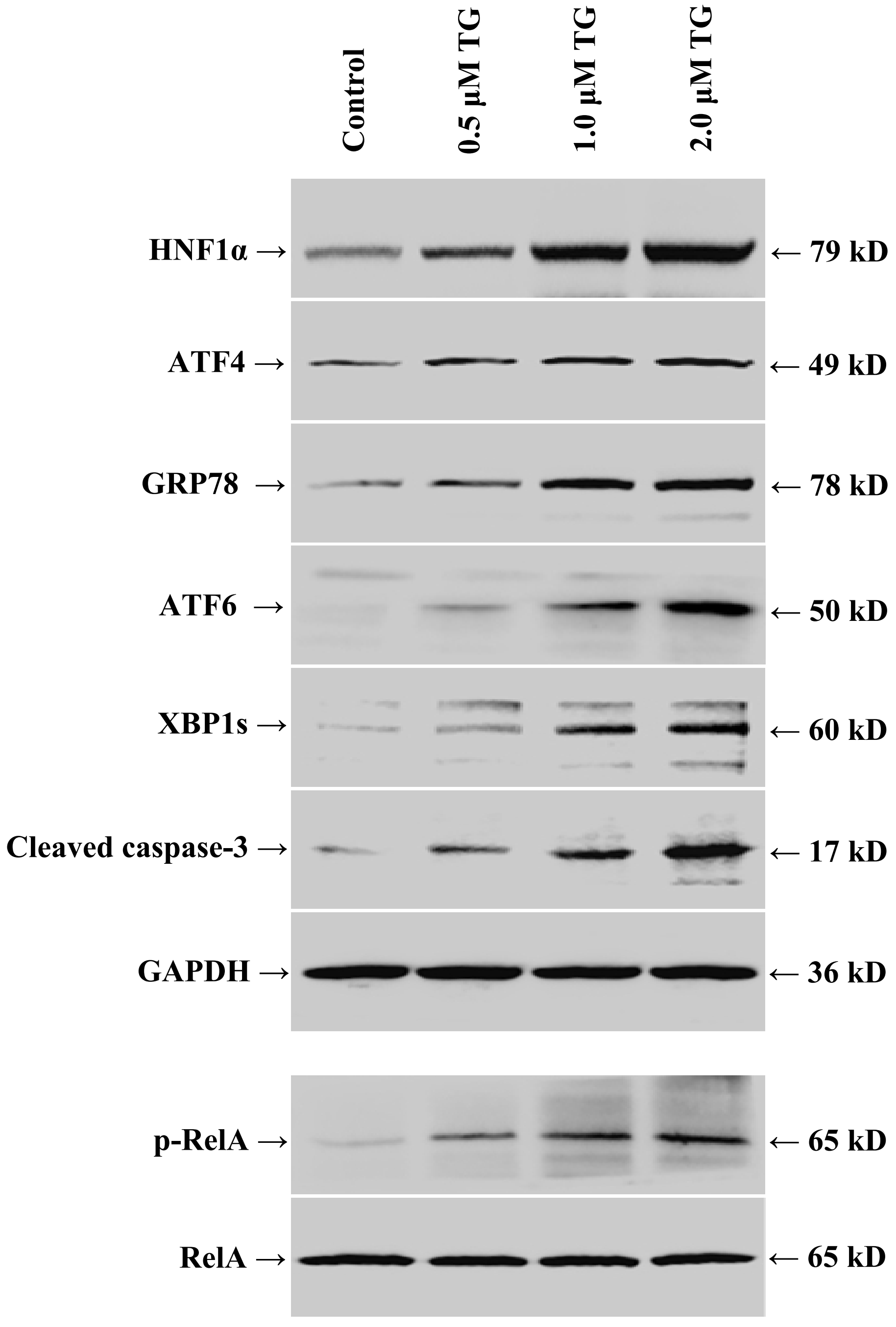

Figure 1F（LO2 cells）

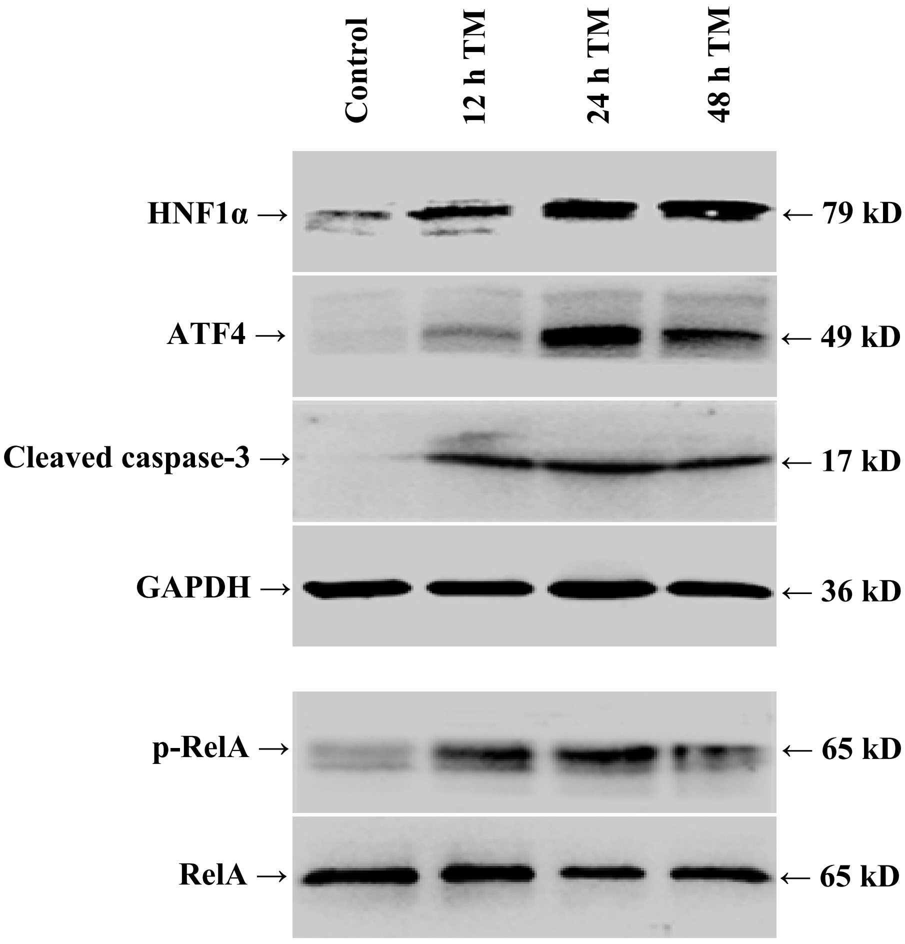

Figure 1H（HepG2 cells）

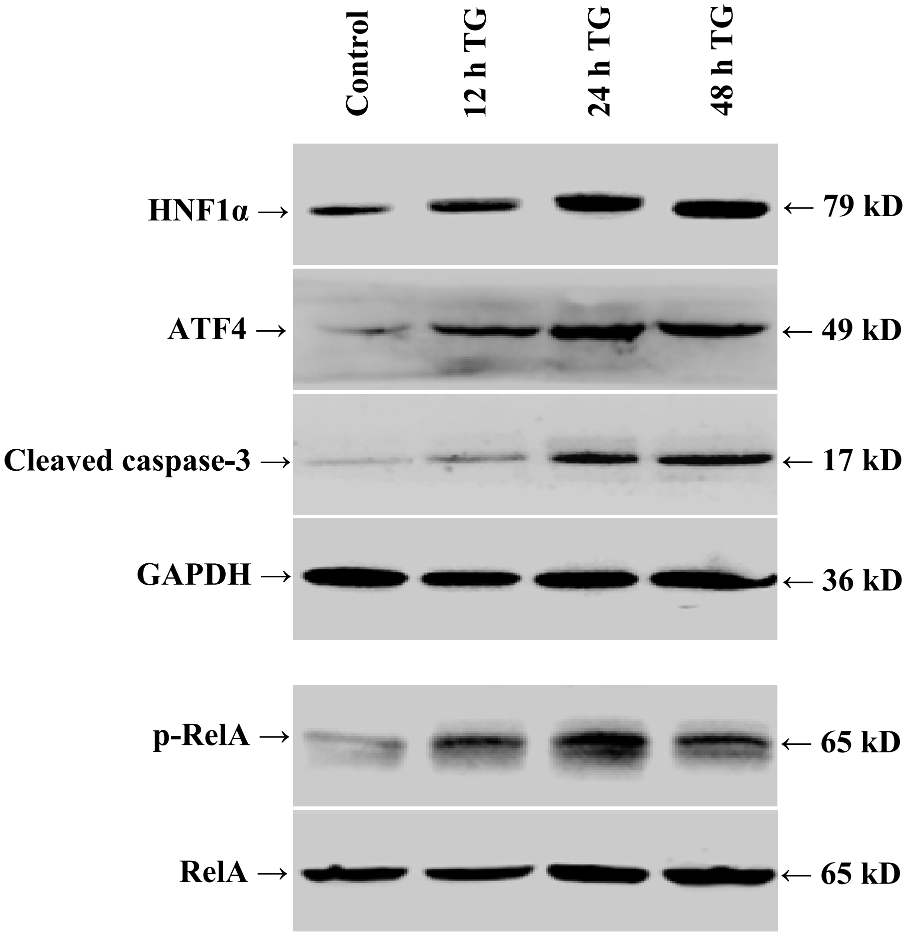

Figure 1J (SK-Hep1 cells)

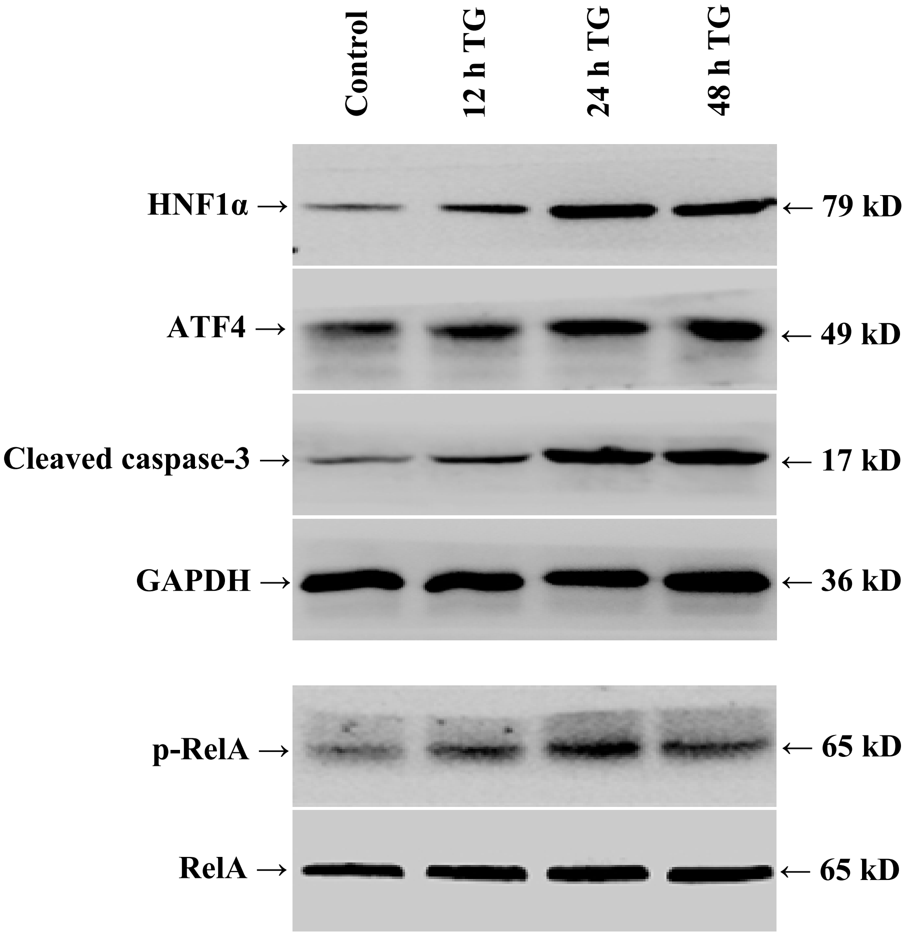

Figure 2A

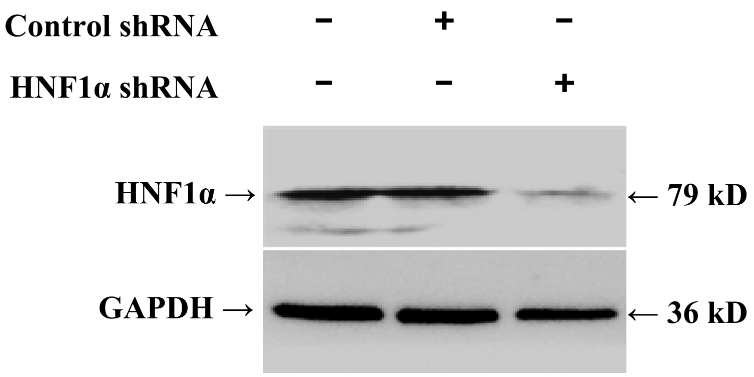

Figure 2C

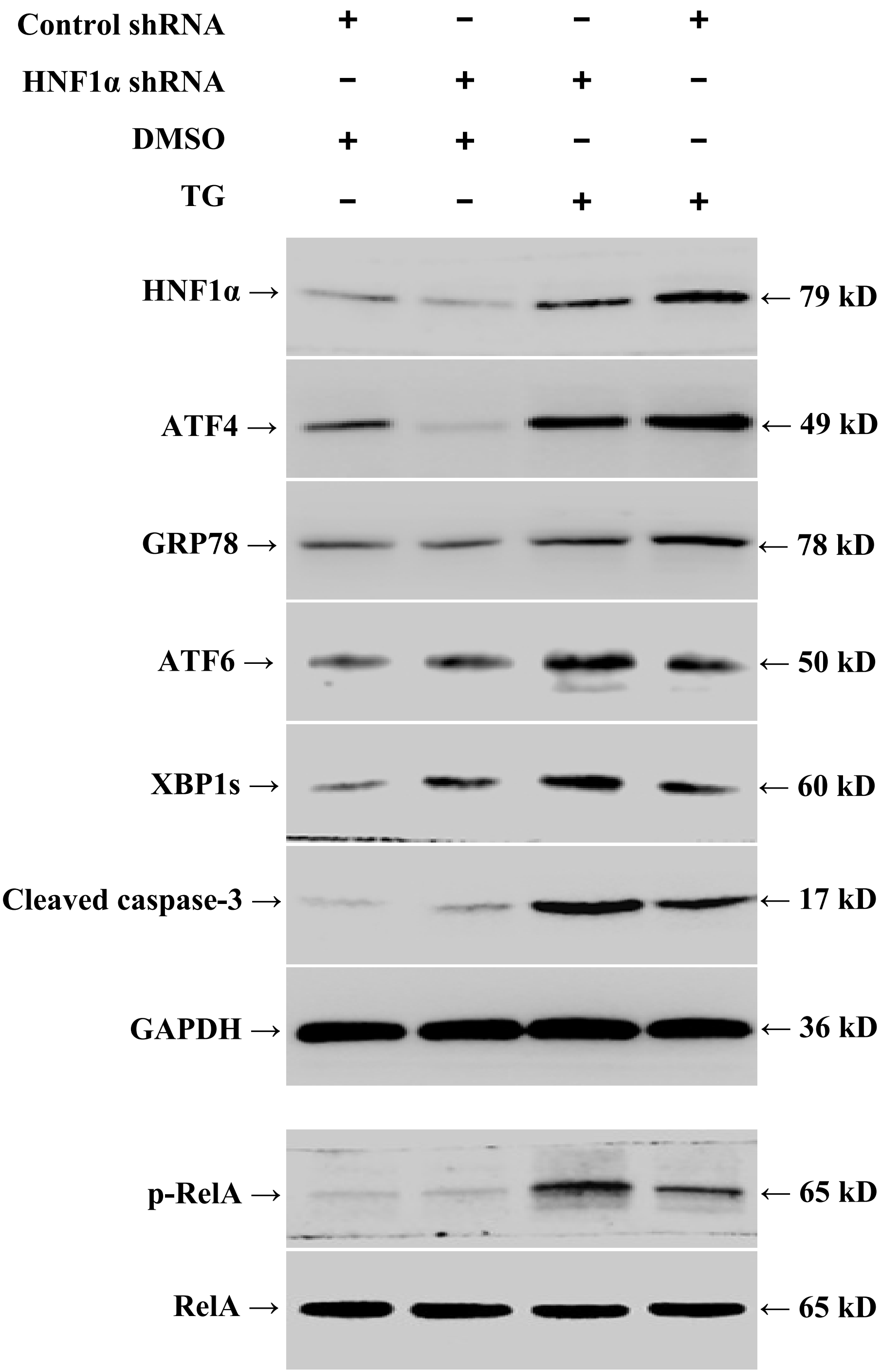

Control shRNA + − − +
HNF1α shRNA − + + −
DMSO + + − −
TG − − + +
HNF1α → ← 79 kD
ATF4 → ← 49 kD
GRP78 → ← 78 kD
ATF6 → ← 50 kD
XBP1s → ← 60 kD
Cleaved caspase-3 → ← 17 kD
GAPDH → ← 36 kD
p-RelA → ← 65 kD
RelA → ← 65 kD

Figure 3A

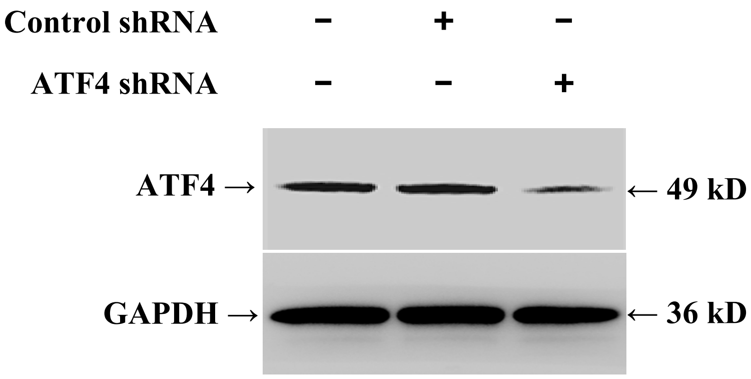

Figure 3C

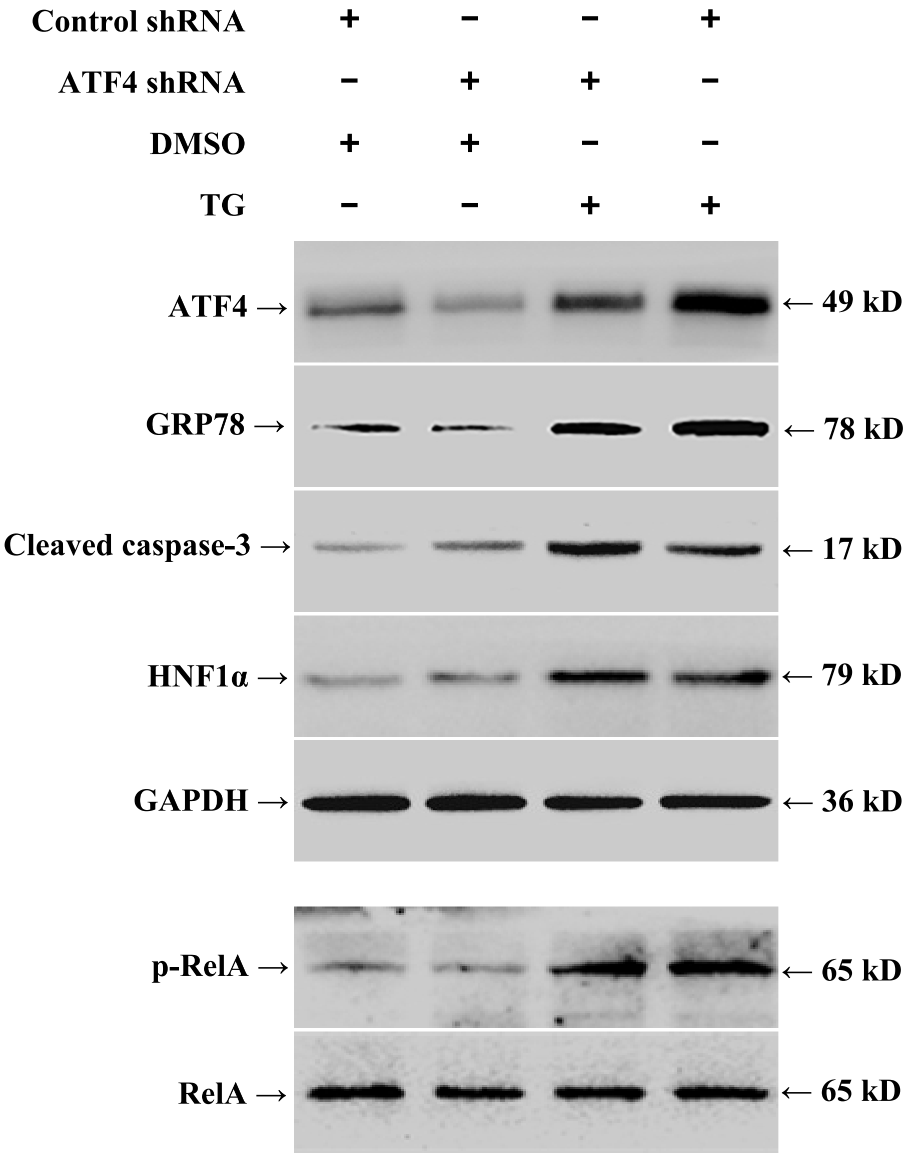

Control shRNA + − − +
ATF4 shRNA − + + −
DMSO + + − −
TG − − + +
ATF4 →
← 49 kD
GRP78 →
← 78 kD
Cleaved caspase-3 →
← 17 kD
HNF1α →
← 79 kD
GAPDH →
← 36 kD
p-RelA →
← 65 kD
RelA →
← 65 kD

Figure 4A

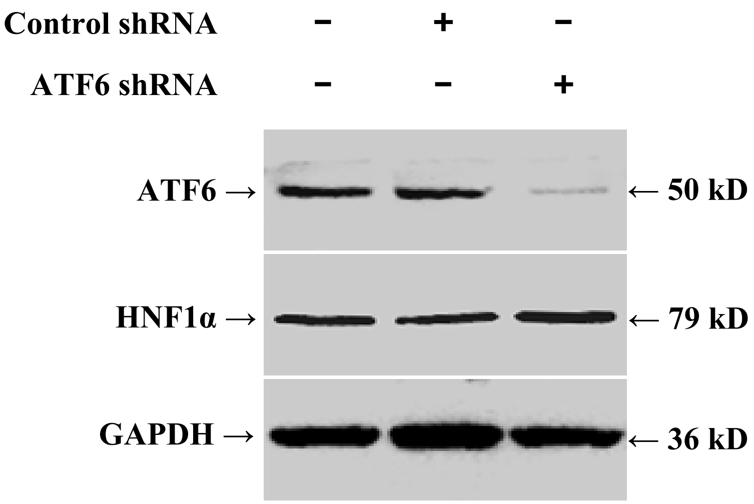

Figure 4B

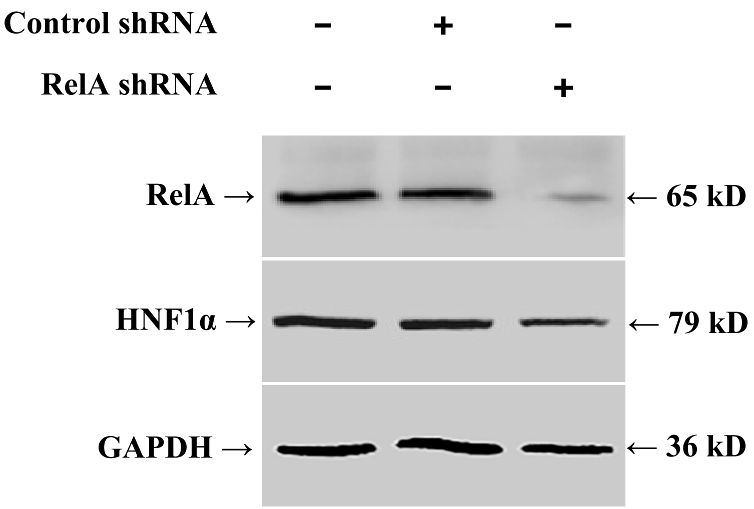

Figure 4D

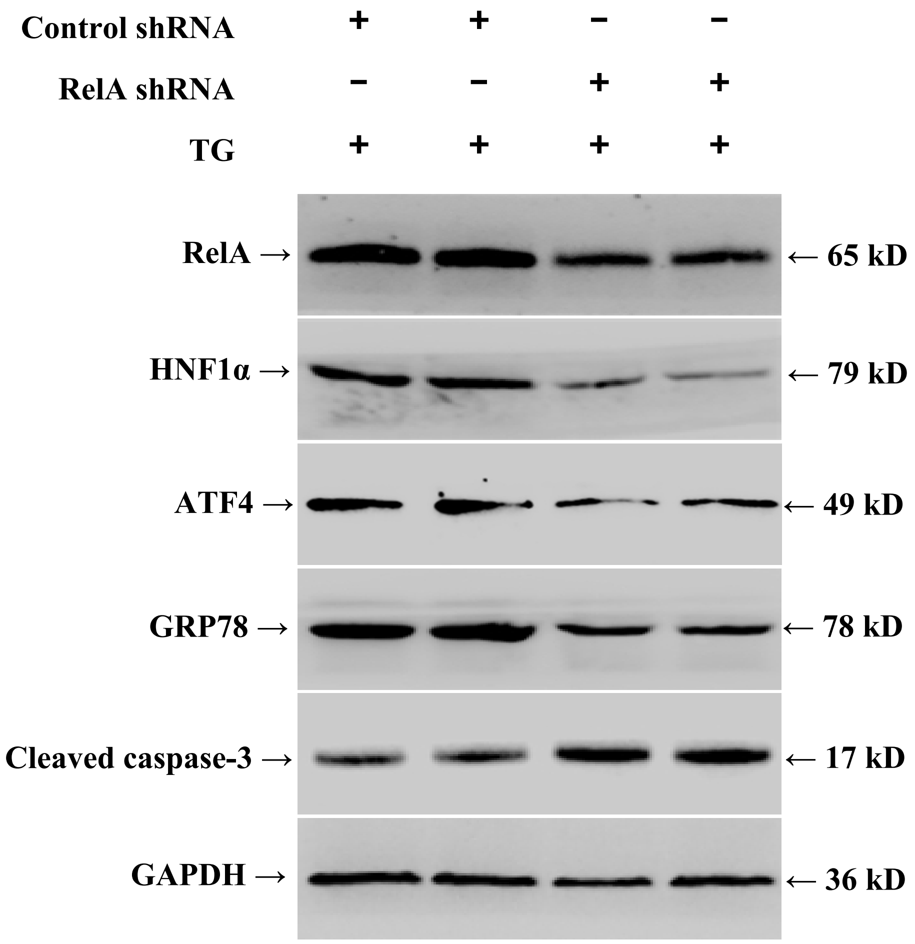

Control shhRNA + + − −
RelA shRNA − − + +
TG + + + +
RelA →
← 65 kD
HNF1α →
← 79 kD
ATF4 →
← 49 kD
GRP78 →
← 78 kD
Cleaved caspase-3 →
← 17 kD
GAPDH →
← 36 kD

Figure 5D (65%)

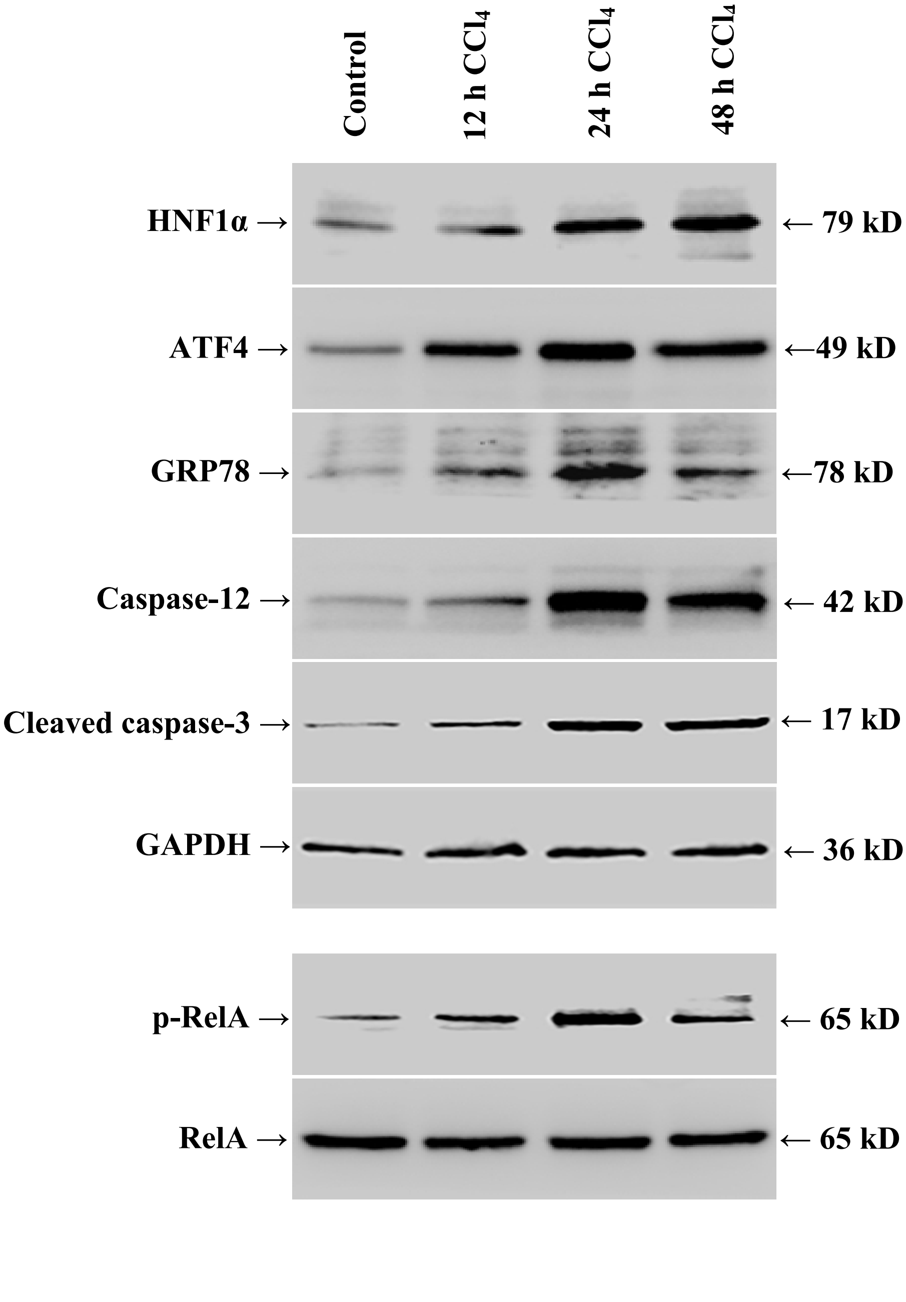
Control
12 h CCl4
24 h CCl4
48 h CCl4
HNF1α →
← 79 kD
ATF4 →
←49 kD
GRP78 →
←78 kD
Caspase-12 →
← 42 kD
Cleaved caspase-3 →
← 17 kD
GAPDH →
← 36 kD
p-RelA →
← 65 kD
RelA →
← 65 kD

Figure 5C(58%)

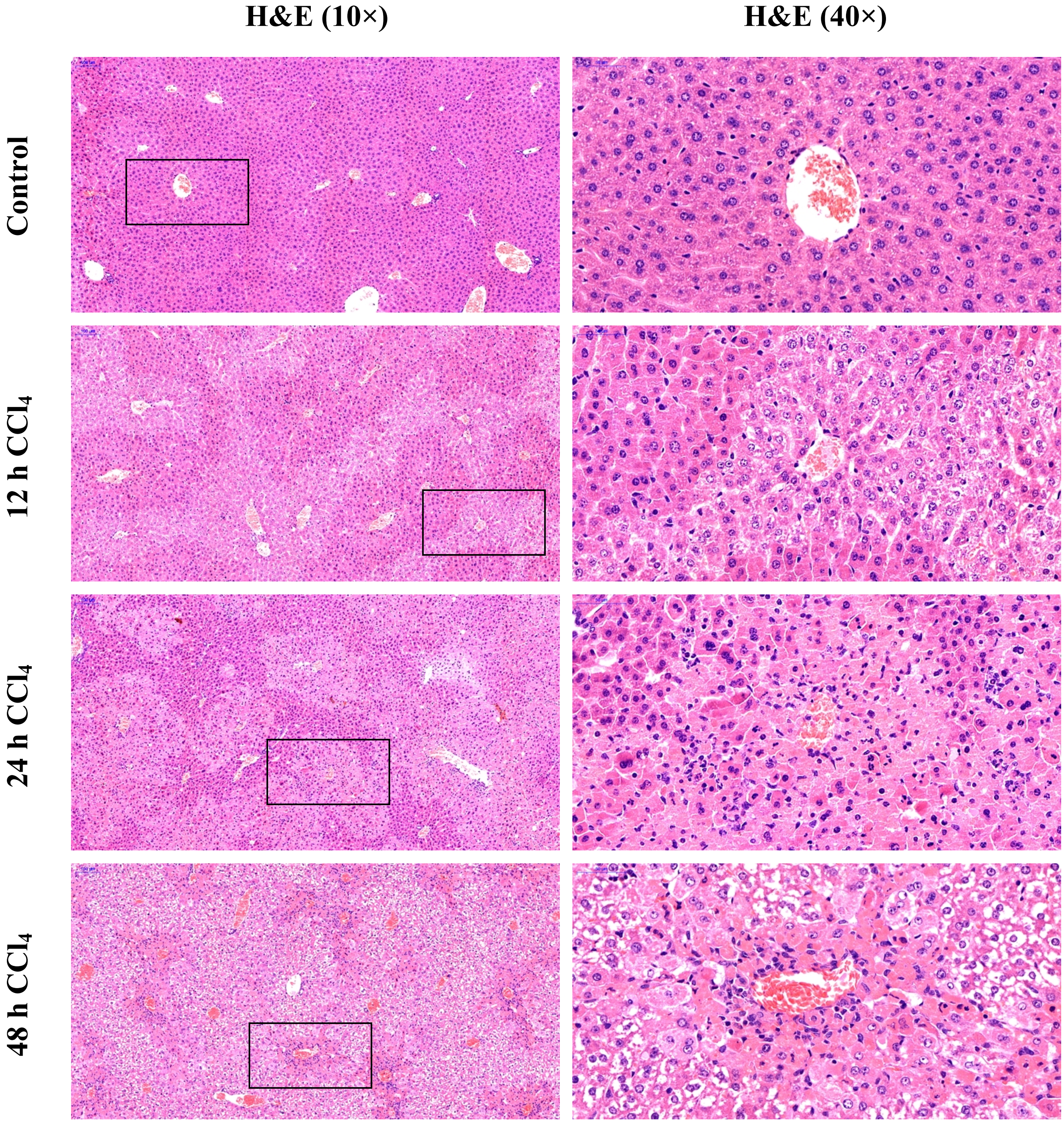

Figure 5I

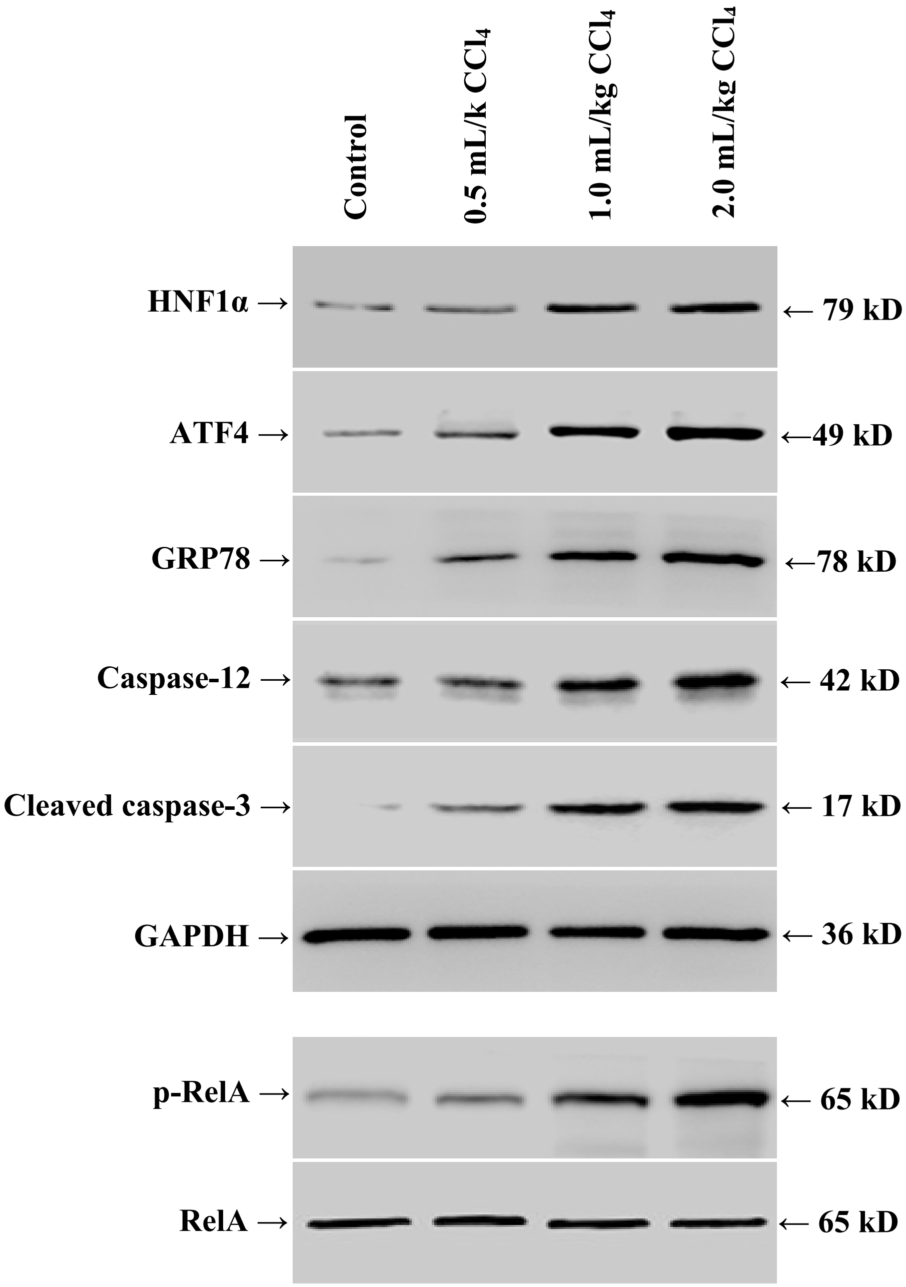

Control
0.5 mL/k CCl_4
1.0 mL/kg CCl_4
2.0 mL/kg CCl_4
HNF1α →
← 79 kD
ATF4 →
←49 kD
GRP78 →
←78 kD
Caspase-12 →
← 42 kD
Cleaved caspase-3 →
← 17 kD
GAPDH →
← 36 kD
p-RelA →
← 65 kD
RelA →
← 65 kD

Figure 5H（58%）

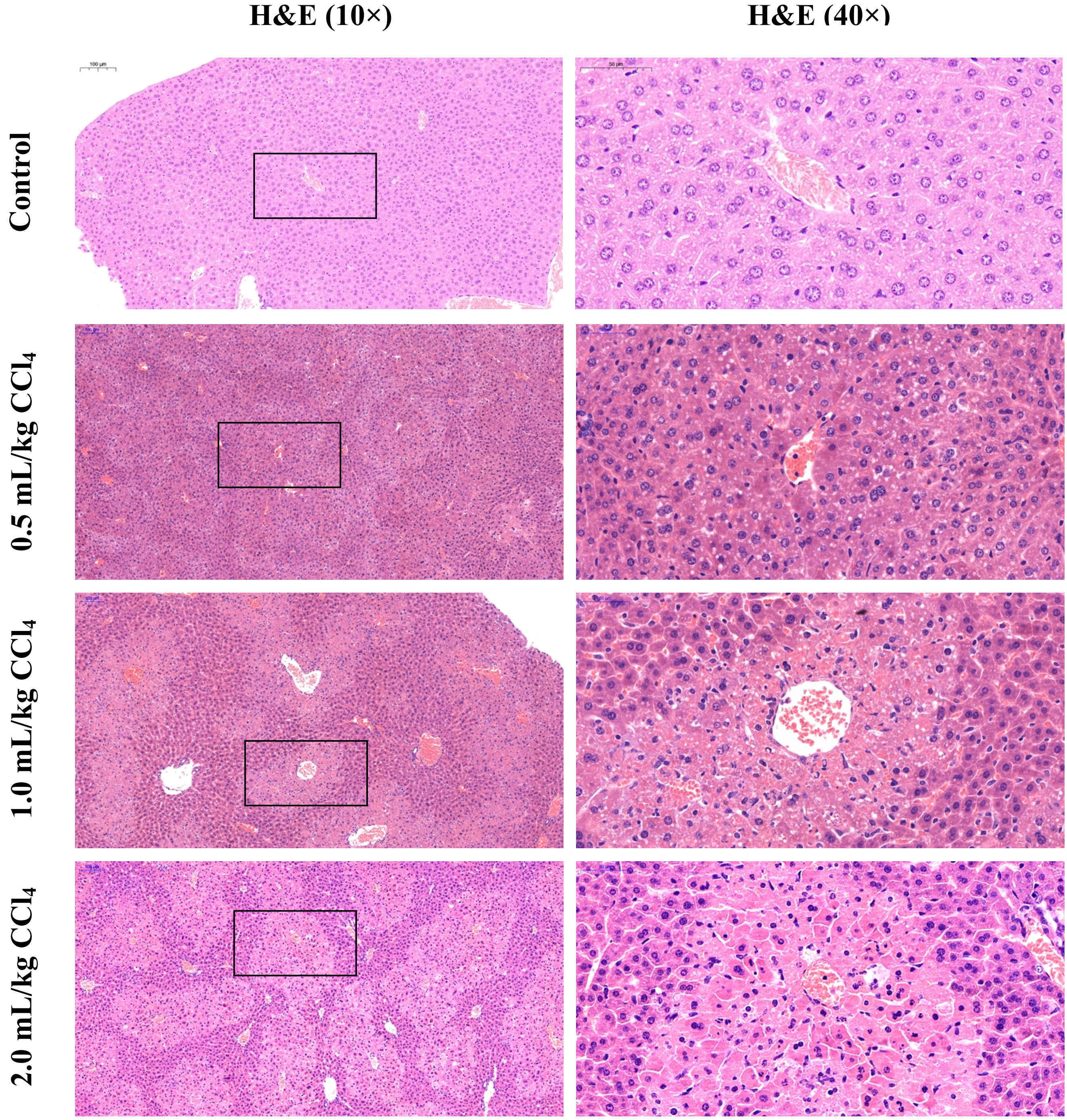
H&E (10×)
H&E (40×)
Control
0.5 mL/kg CCl4
1.0 mL/kg CCl4
2.0 mL/kg CCl4
100 μm
50 μm

Figure 5K（58%）

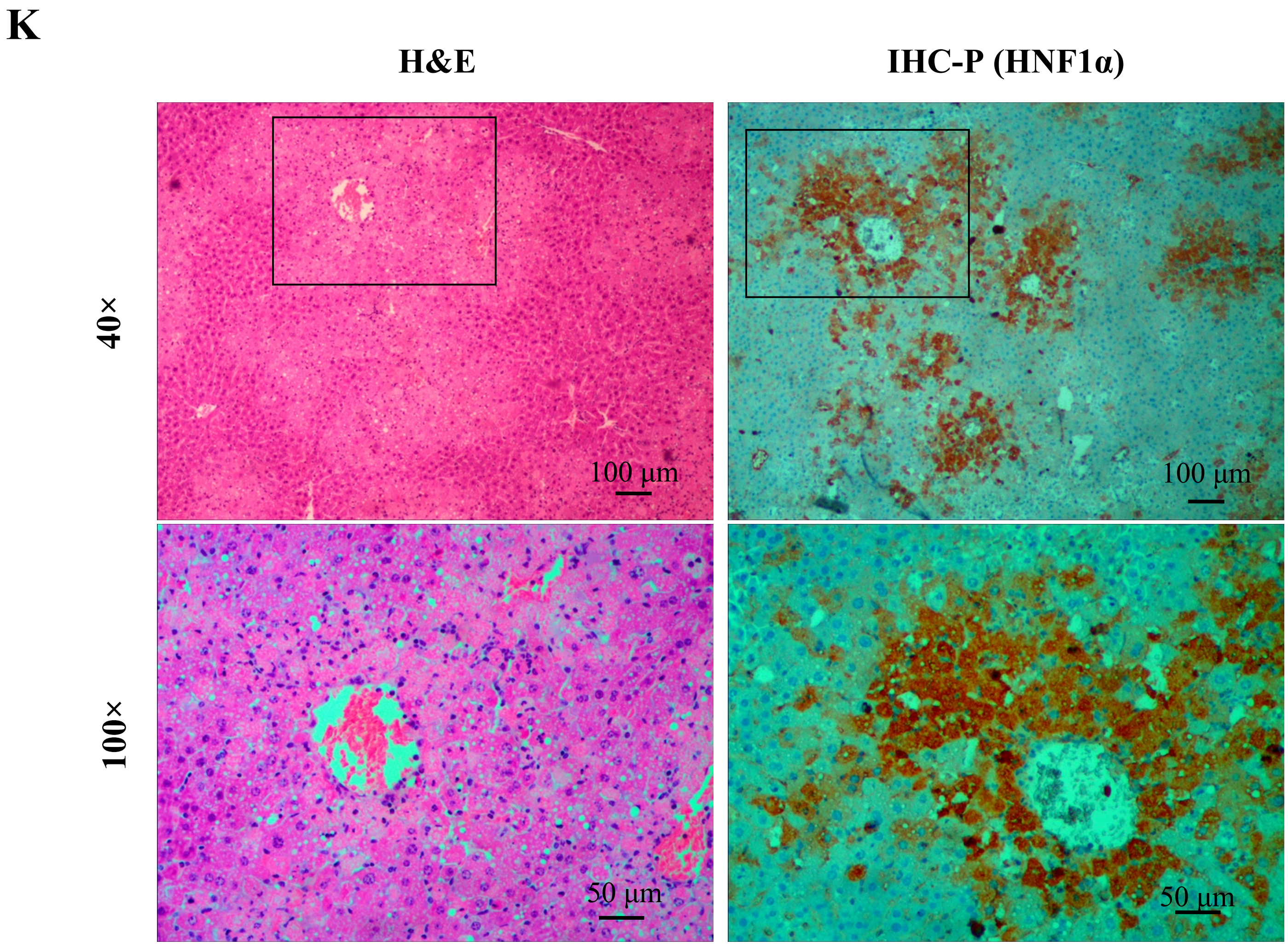

Figure 5L

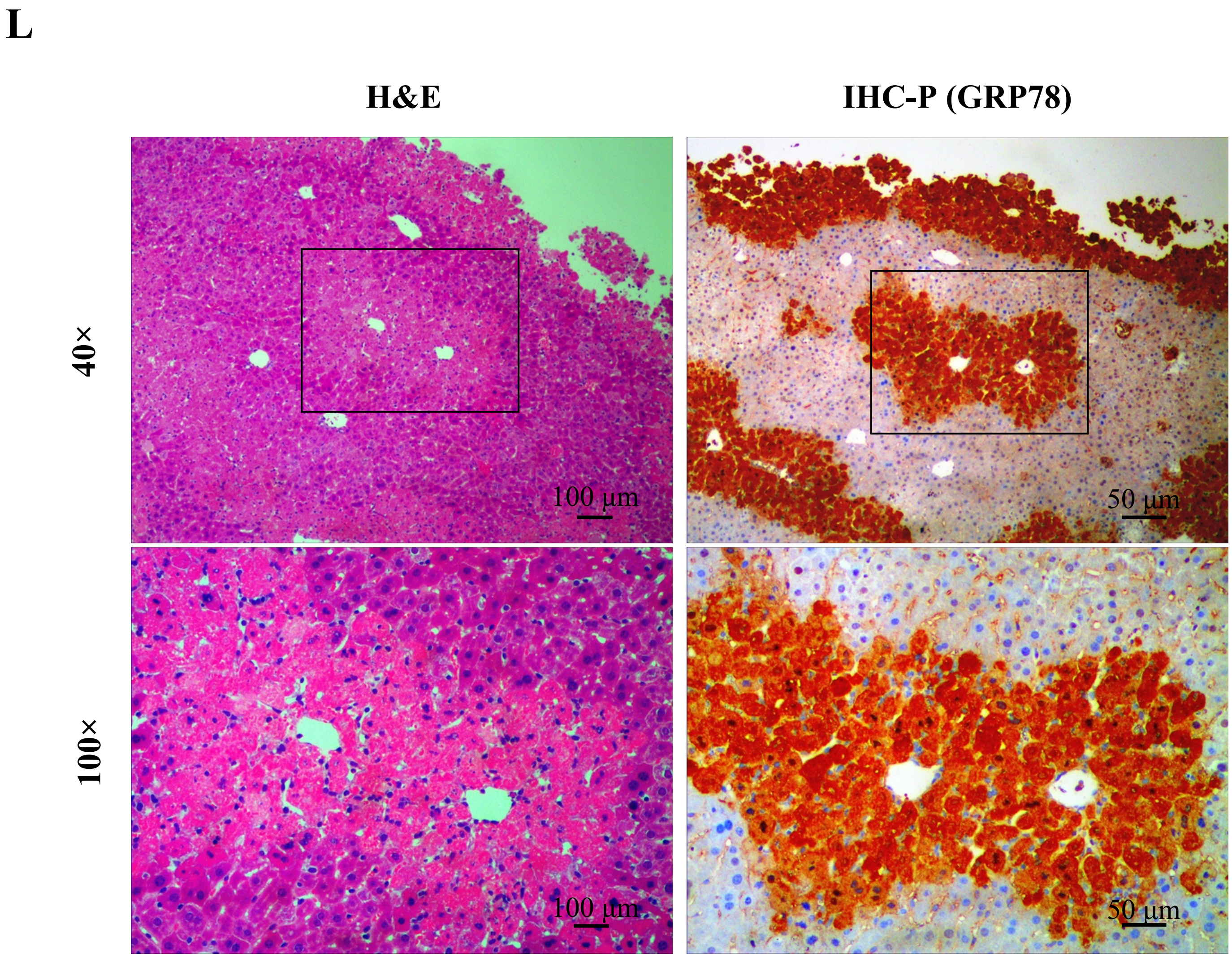

Figure 5E（70%）

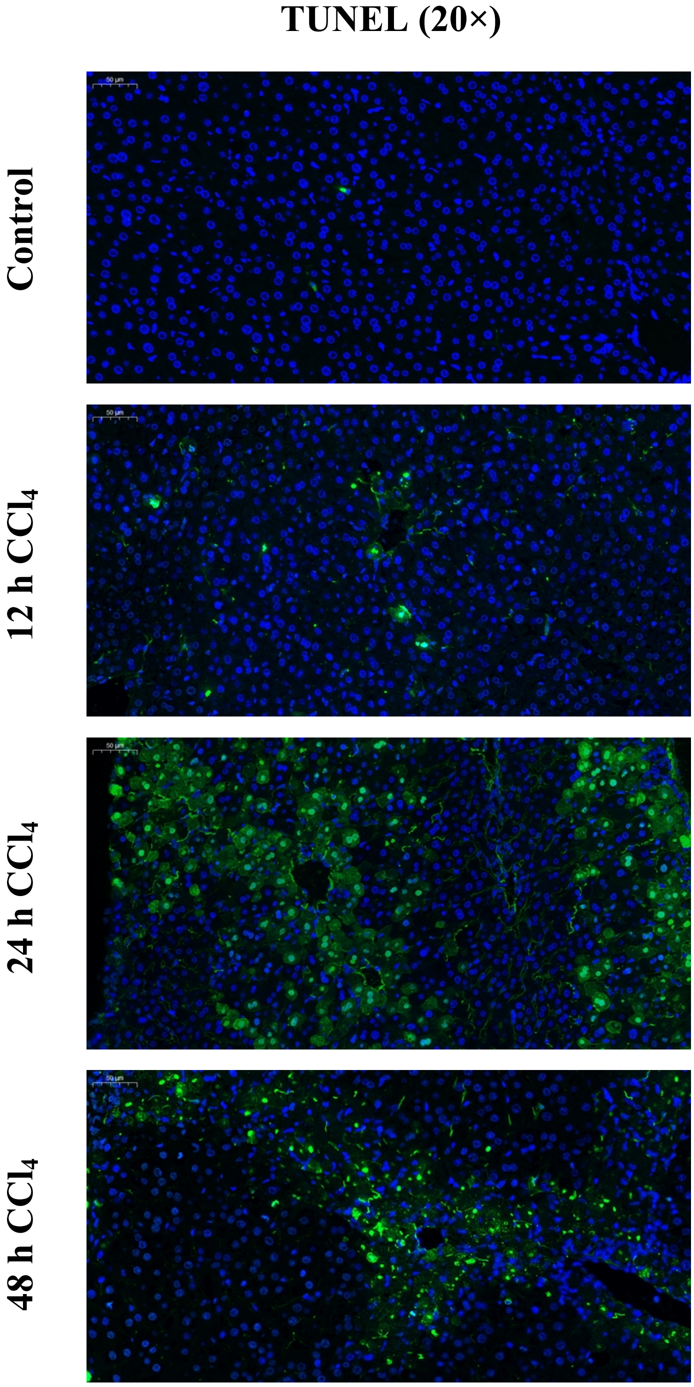

Figure 5J

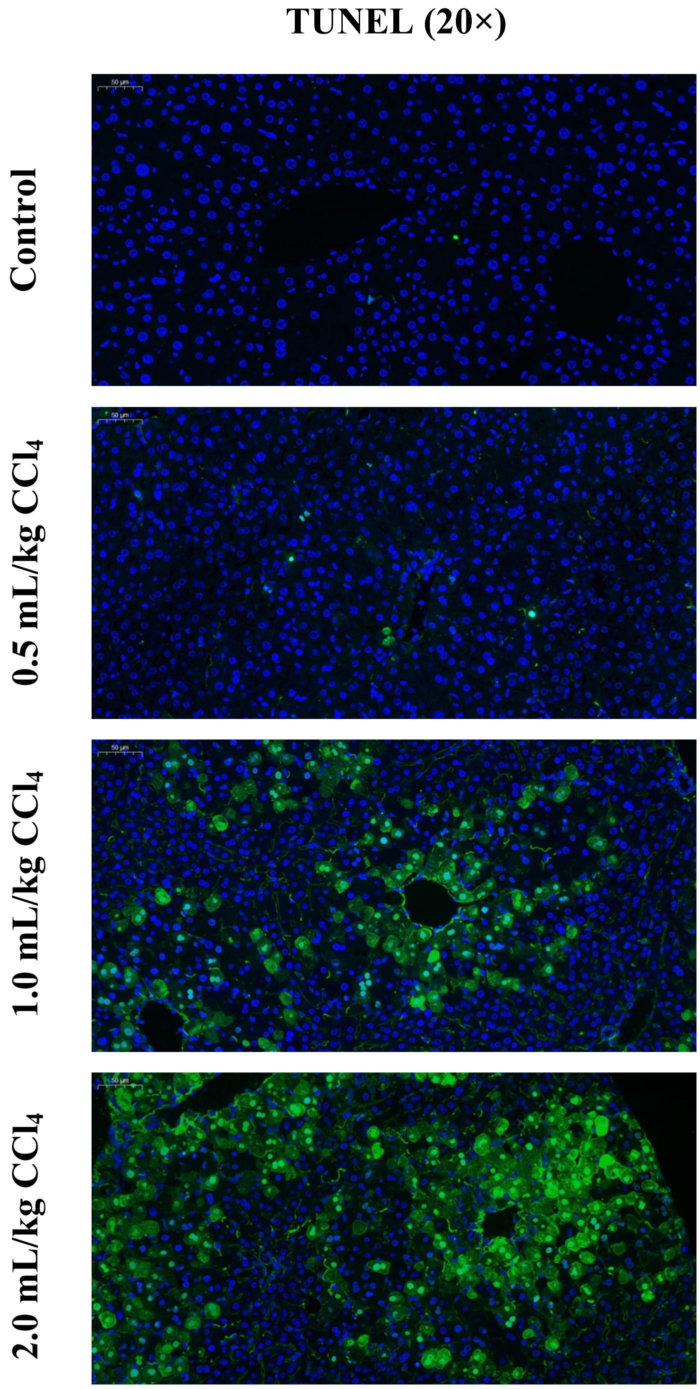

Figure 6A

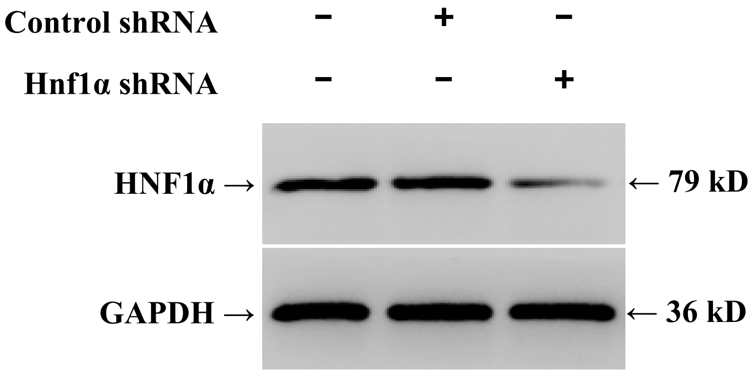

Figure 6D (58%)

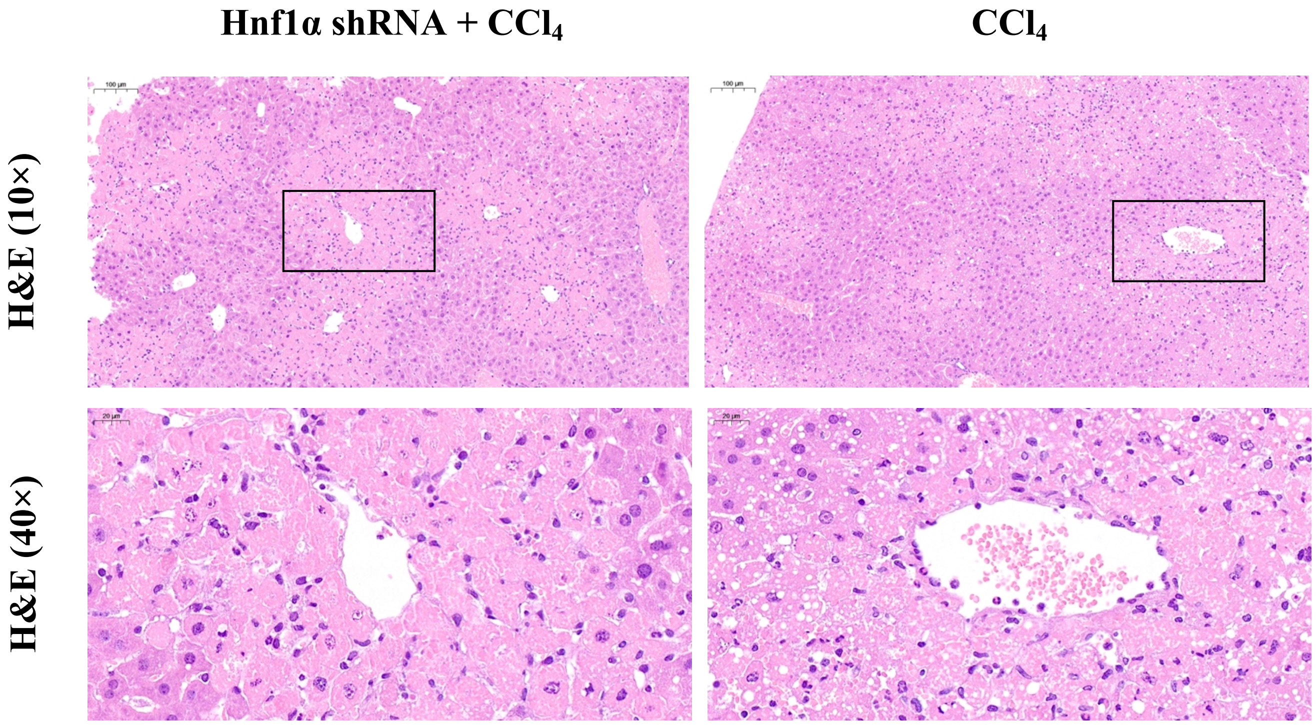

Figure 6E (65%)

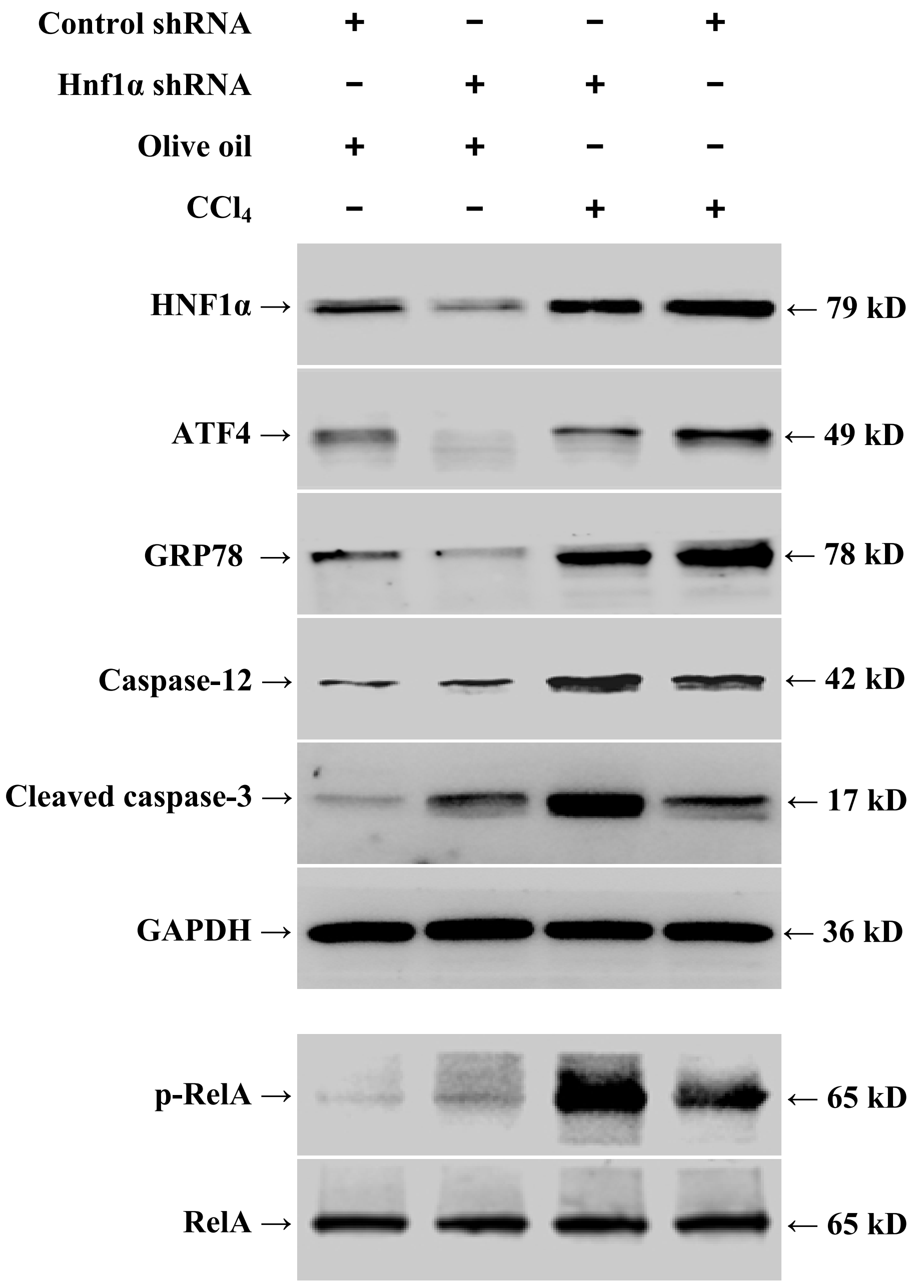
Control shRNA + − − +
Hnf1α shRNA − + + −
Olive oil + + − −
CCl4 − − + +
HNF1α → ← 79 kD
ATF4 → ← 49 kD
GRP78 → ← 78 kD
Caspase-12 → ← 42 kD
Cleaved caspase-3 → ← 17 kD
GAPDH → ← 36 kD
p-RelA → ← 65 kD
RelA → ← 65 kD

Figure 6F

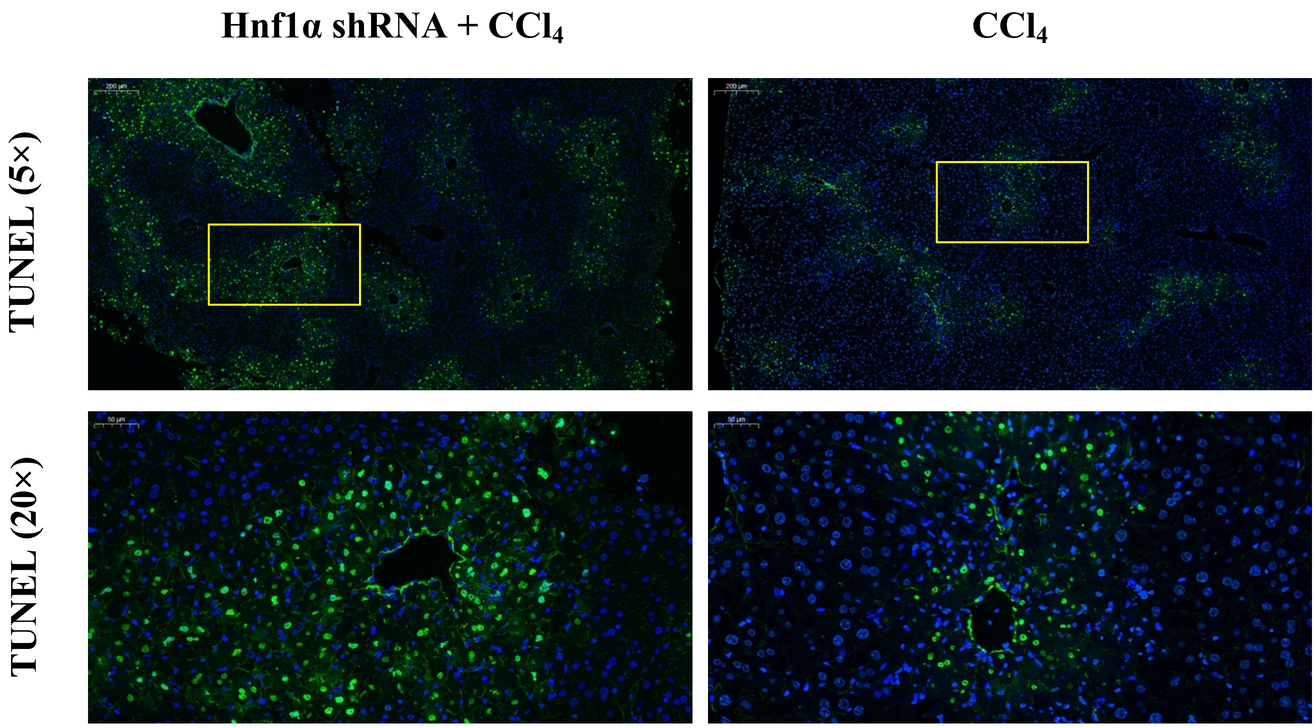

Figute 7C（50%）

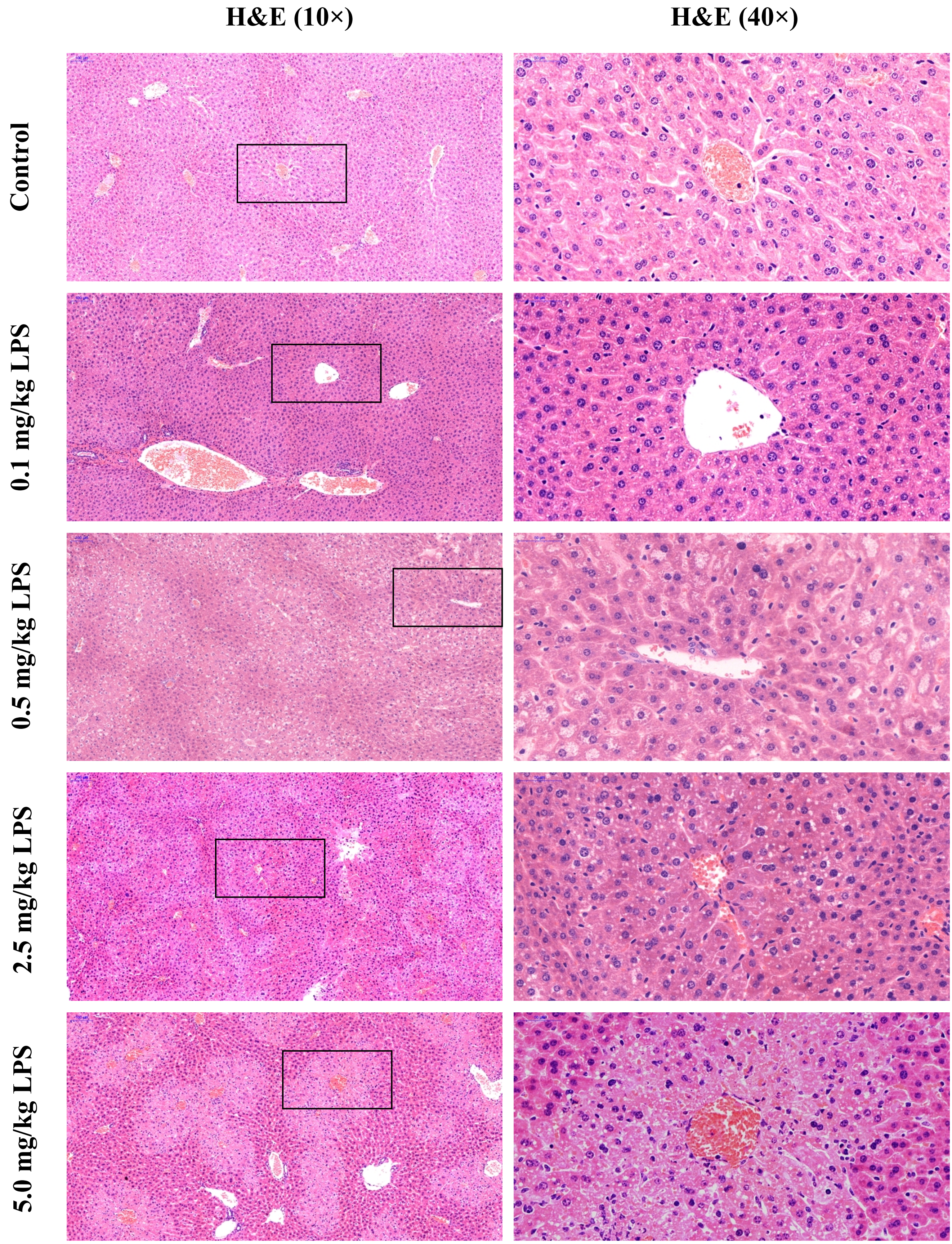

H&E (10×)
H&E (40×)
Control
0.1 mg/kg LPS
0.5 mg/kg LPS
2.5 mg/kg LPS
5.0 mg/kg LPS

Figure 7D

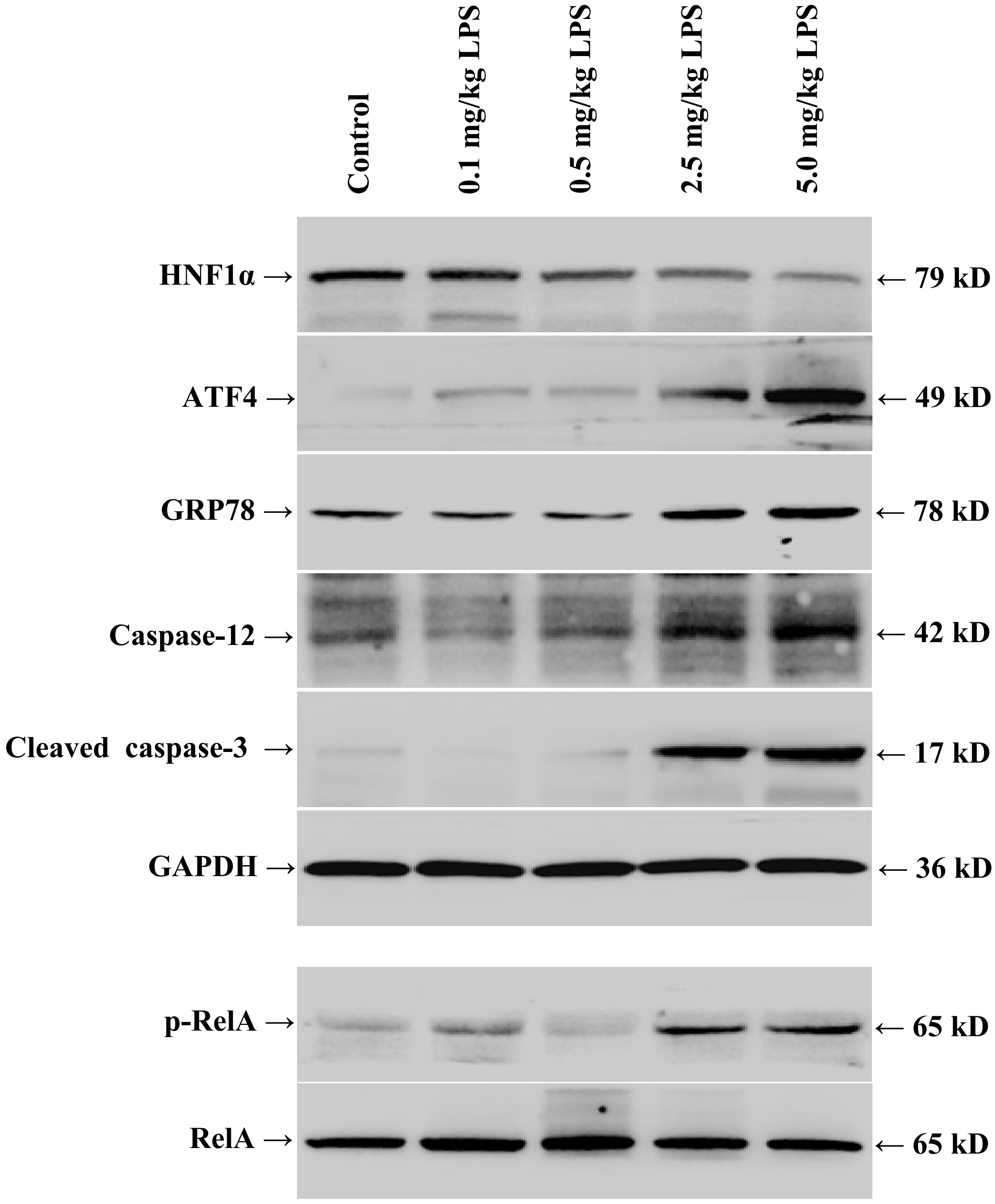
Control
0.1 mg/kg LPS
0.5 mg/kg LPS
2.5 mg/kg LPS
5.0 mg/kg LPS
HNF1α →
← 79 kD
ATF4 →
← 49 kD
GRP78 →
← 78 kD
Caspase-12 →
← 42 kD
Cleaved caspase-3 →
← 17 kD
GAPDH →
← 36 kD
p-RelA →
← 65 kD
RelA →
← 65 kD

Fgure 7E(70%)

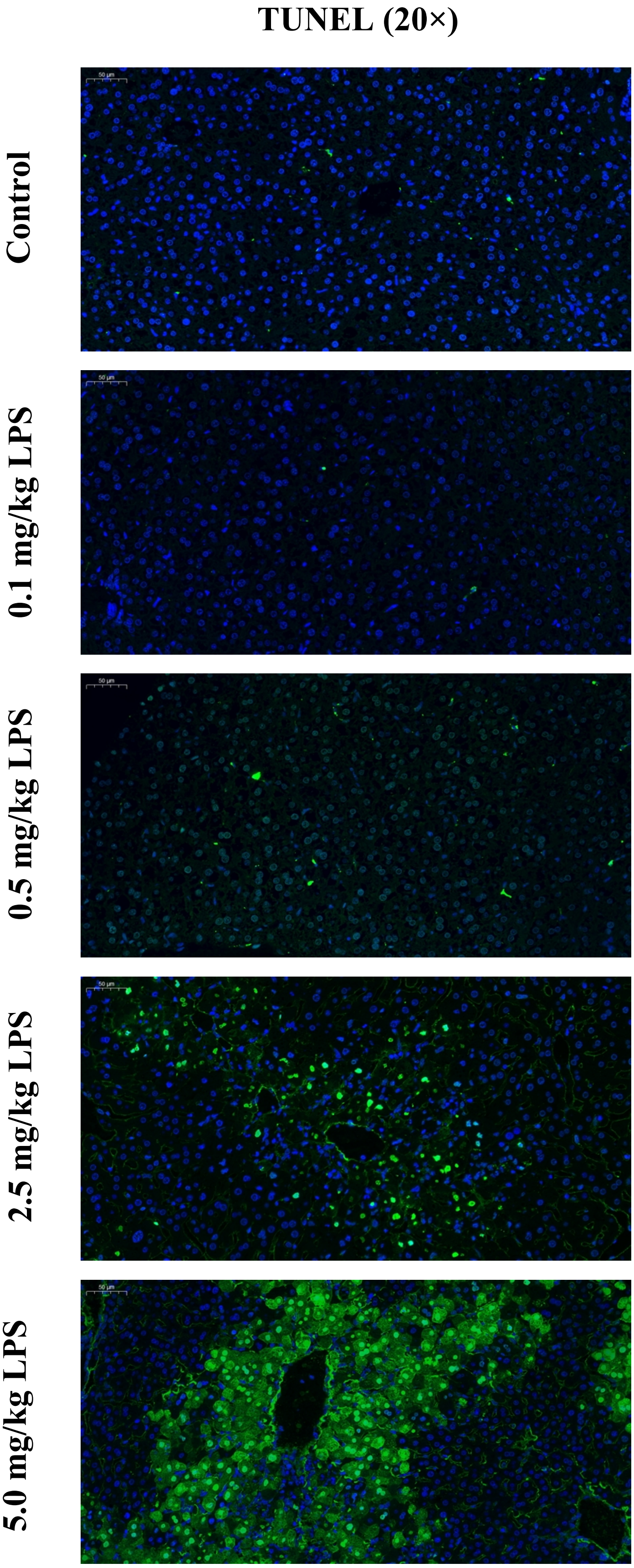
TUNEL (20×)
Control
0.1 mg/kg LPS
0.5 mg/kg LPS
2.5 mg/kg LPS
5.0 mg/kg LPS

Figure 8C （65%）

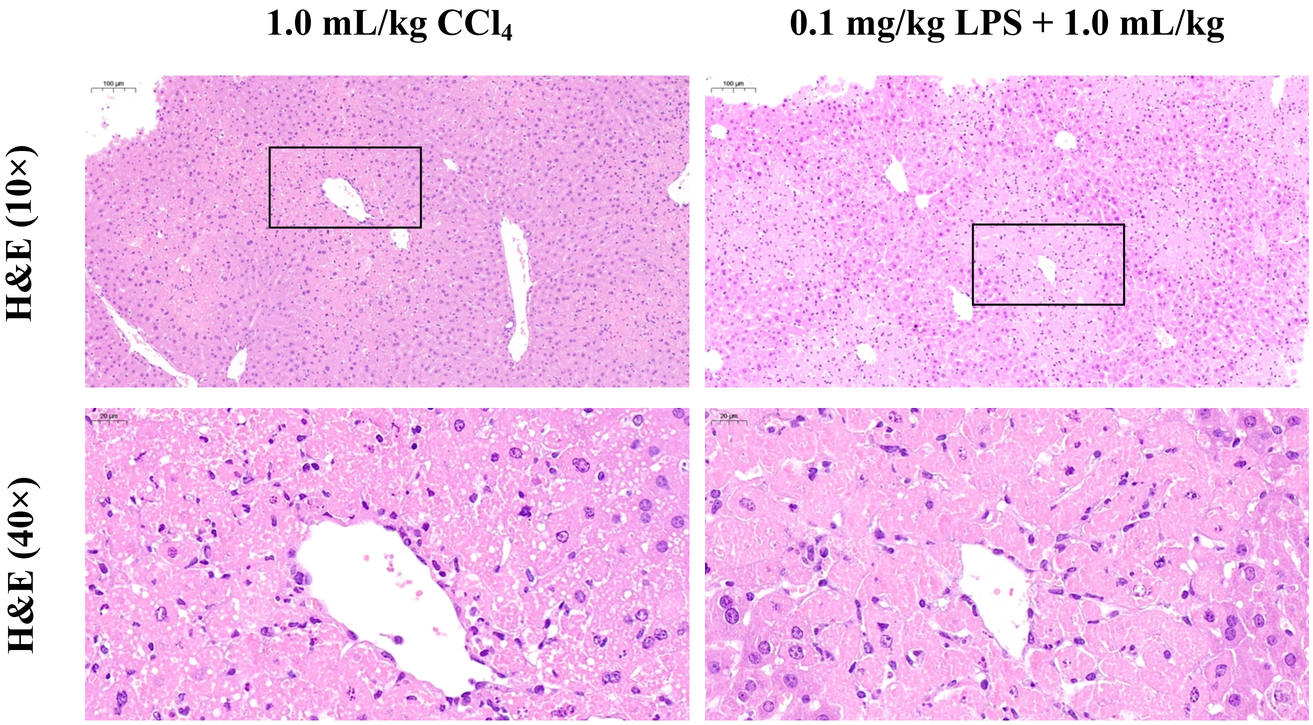

Figure 8D

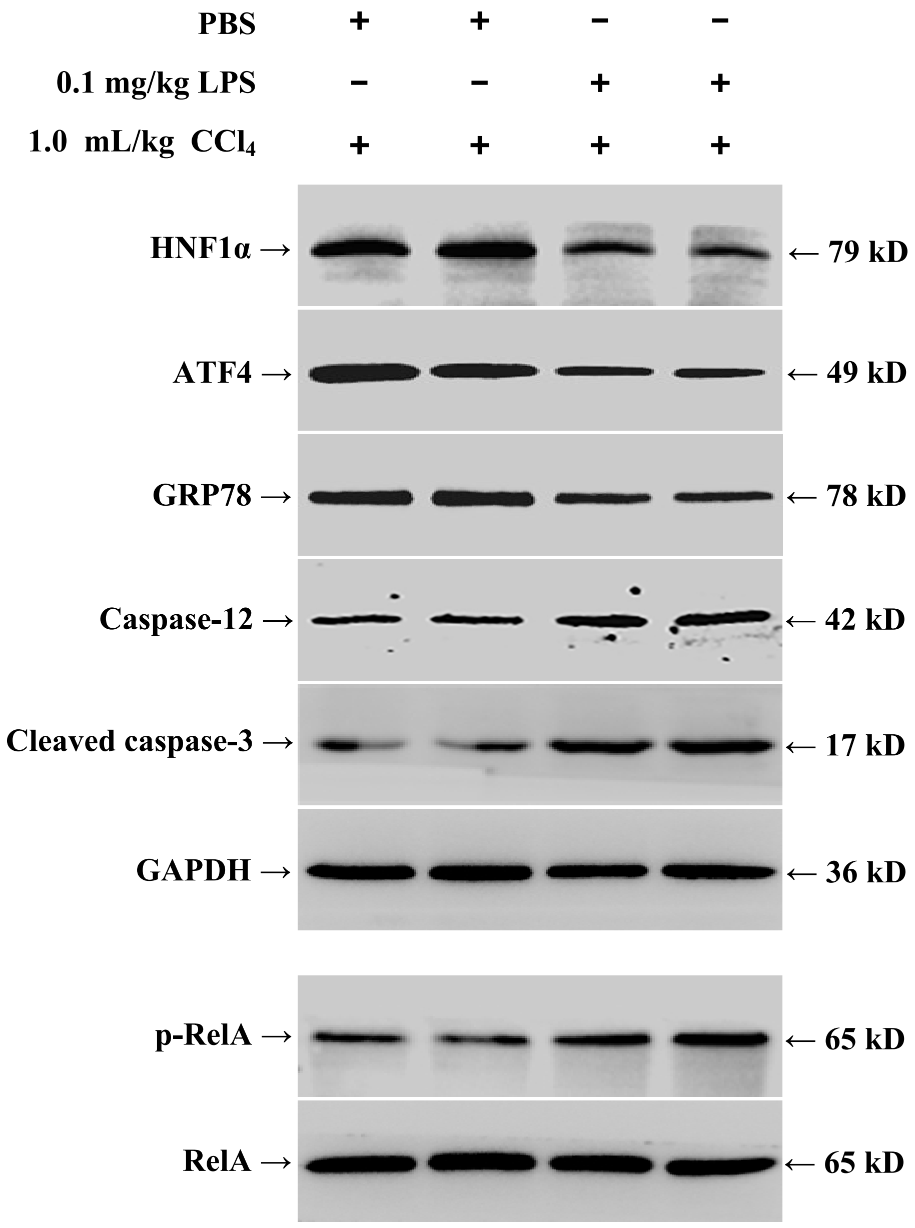
PBS + + − −
0.1 mg/kg LPS − − + +
1.0 mL/kg CCl_4 + + + +
HNF1α →
← 79 kD
ATF4 →
← 49 kD
GRP78 →
← 78 kD
Caspase-12 →
← 42 kD
Cleaved caspase-3 →
← 17 kD
GAPDH →
← 36 kD
p-RelA →
← 65 kD
RelA →
← 65 kD

Figure 8E

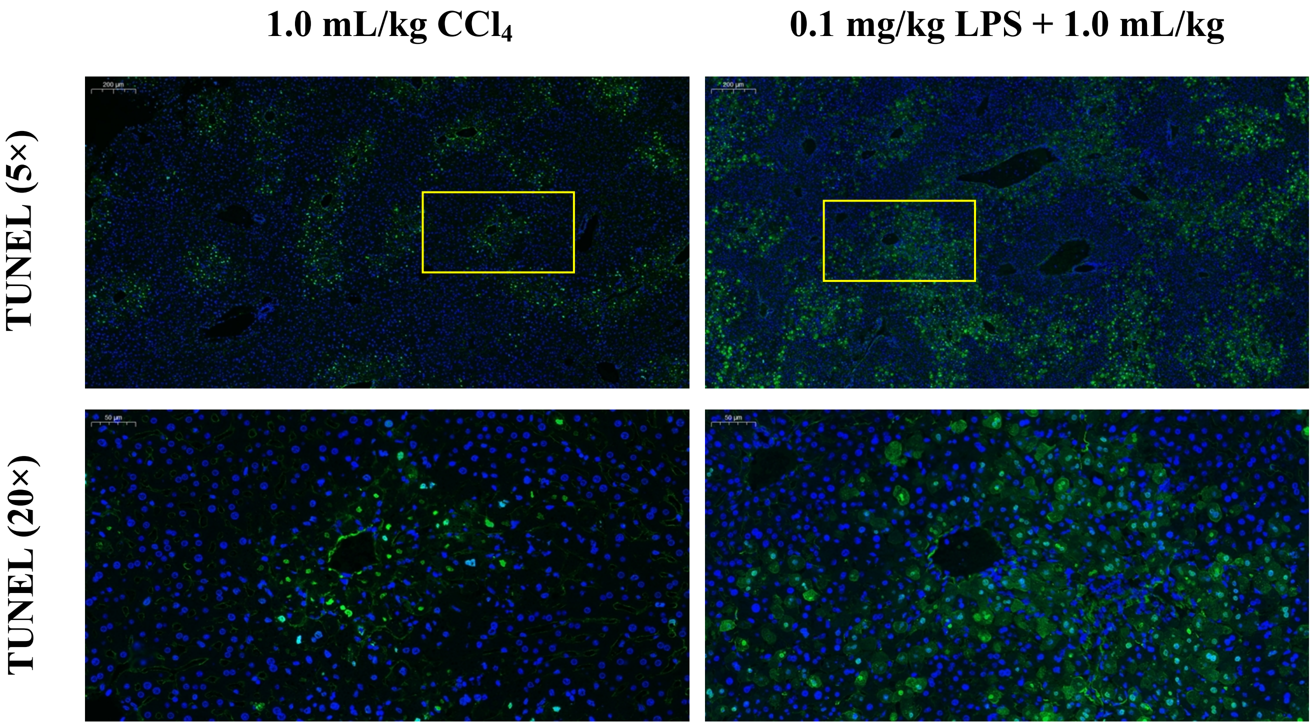

Supplement: Supplementary file 1 — Supplementary Information 1. [file 41598_2022_15846_MOESM1_ESM.pdf]
