# Supplementary material for: Feedback loop between hepatocyte nuclear factor 1α and endoplasmic reticulum stress mitigates liver injury by downregulating hepatocyte apoptosis
Source: Sci Rep. 2022 Jul 8;12:11602. doi: 10.1038/s41598-022-15846-8 (PMC9270423; doi:10.1038/s41598-022-15846-8)

Figure 1B

HNF1α

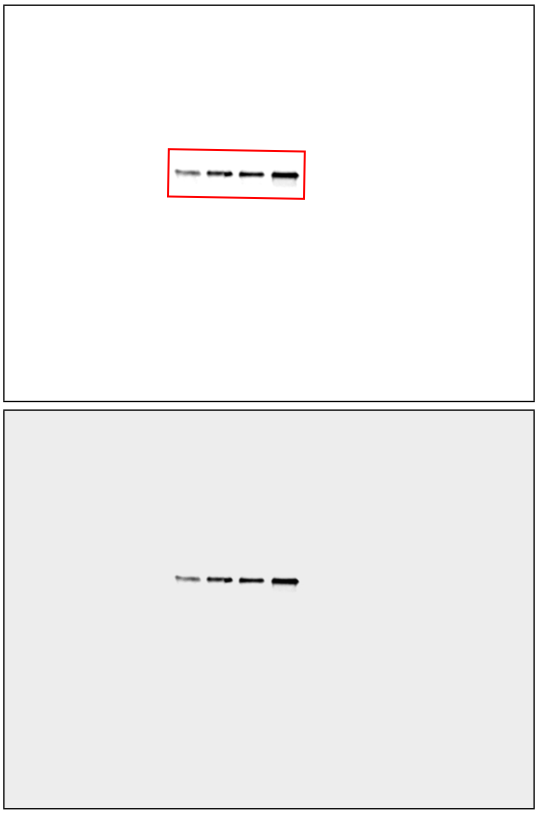

ATF4

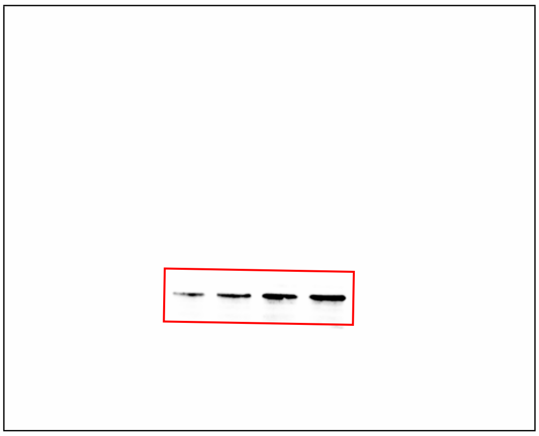

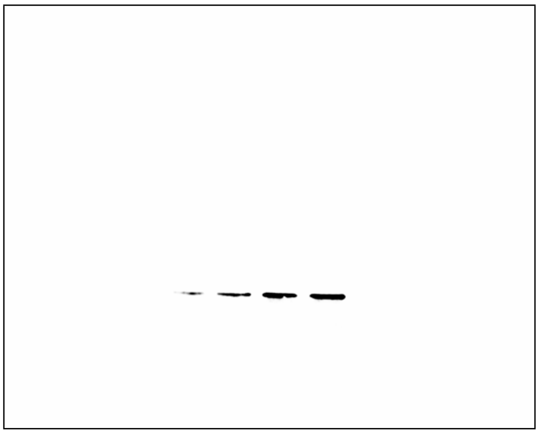

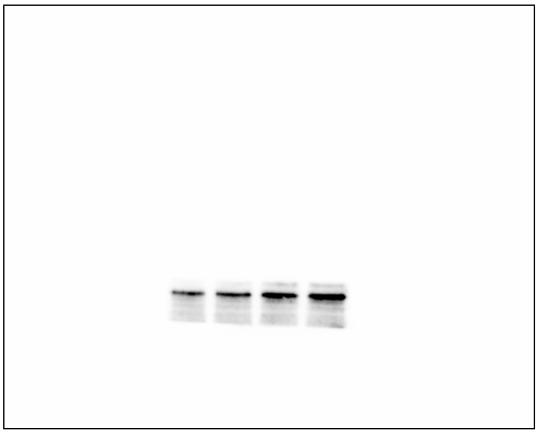

GRP78

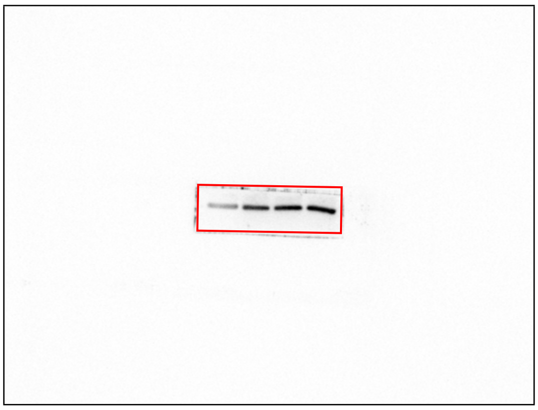

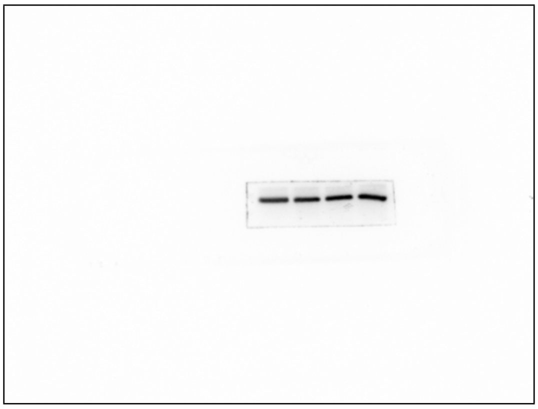

ATF6

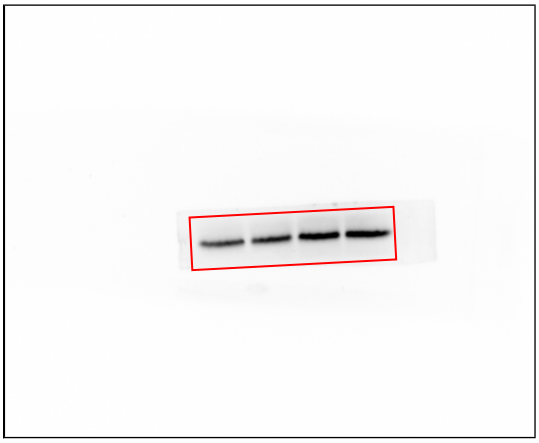

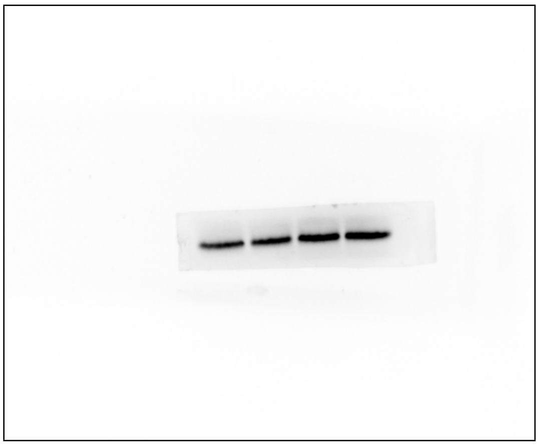

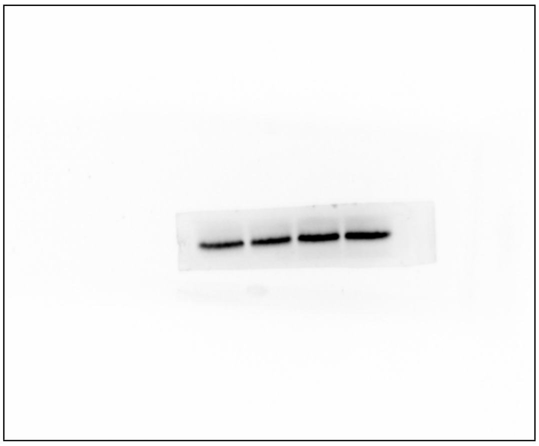

XBP1s

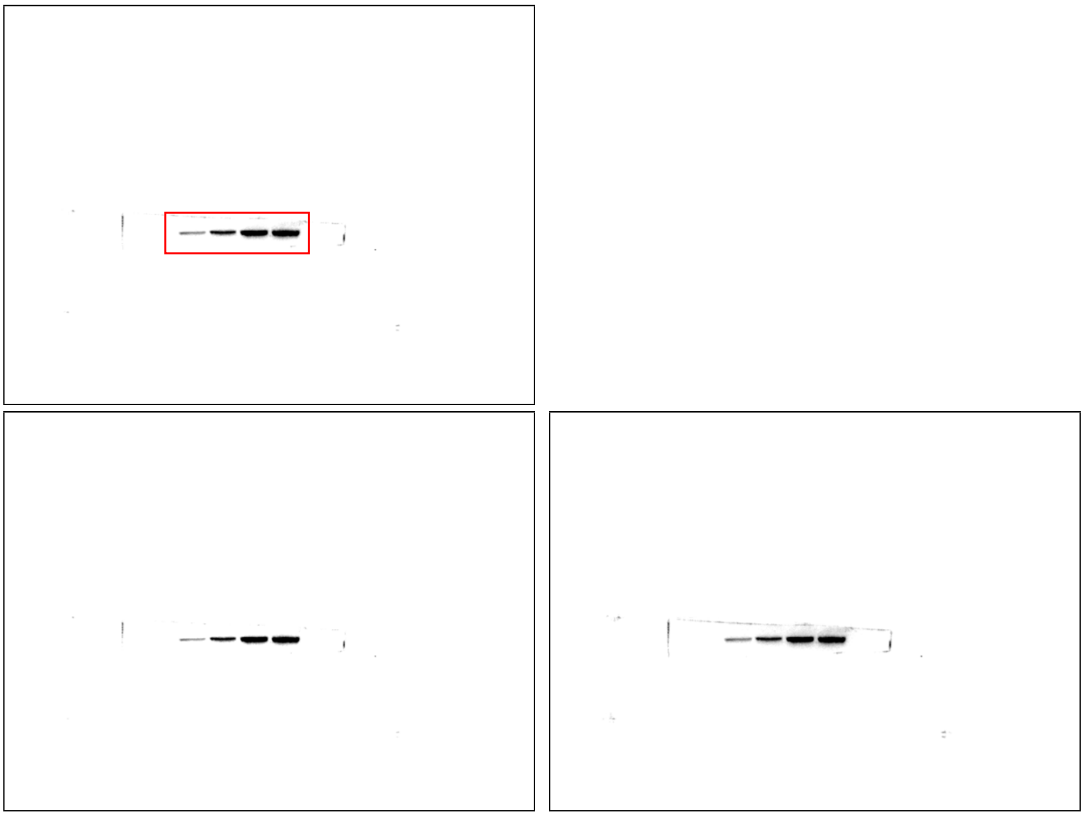

Cleaved caspase-3

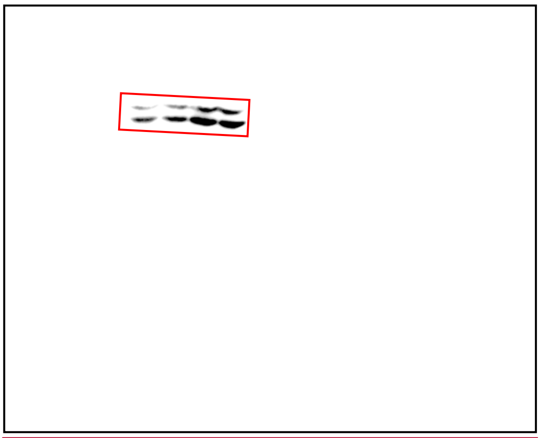

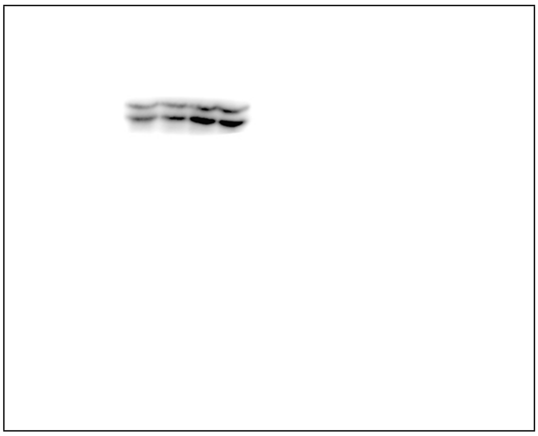

GAPDH

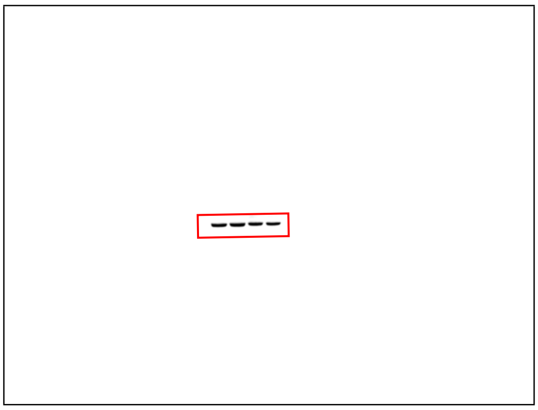

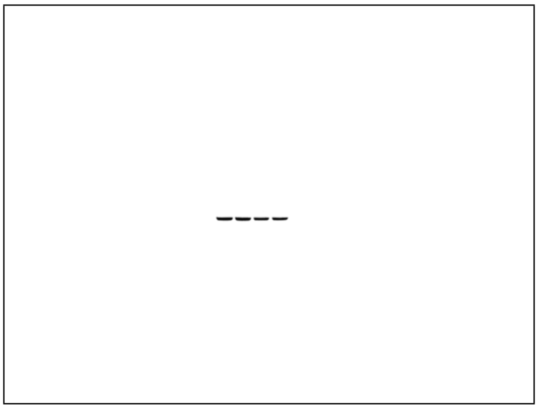

p-RelA

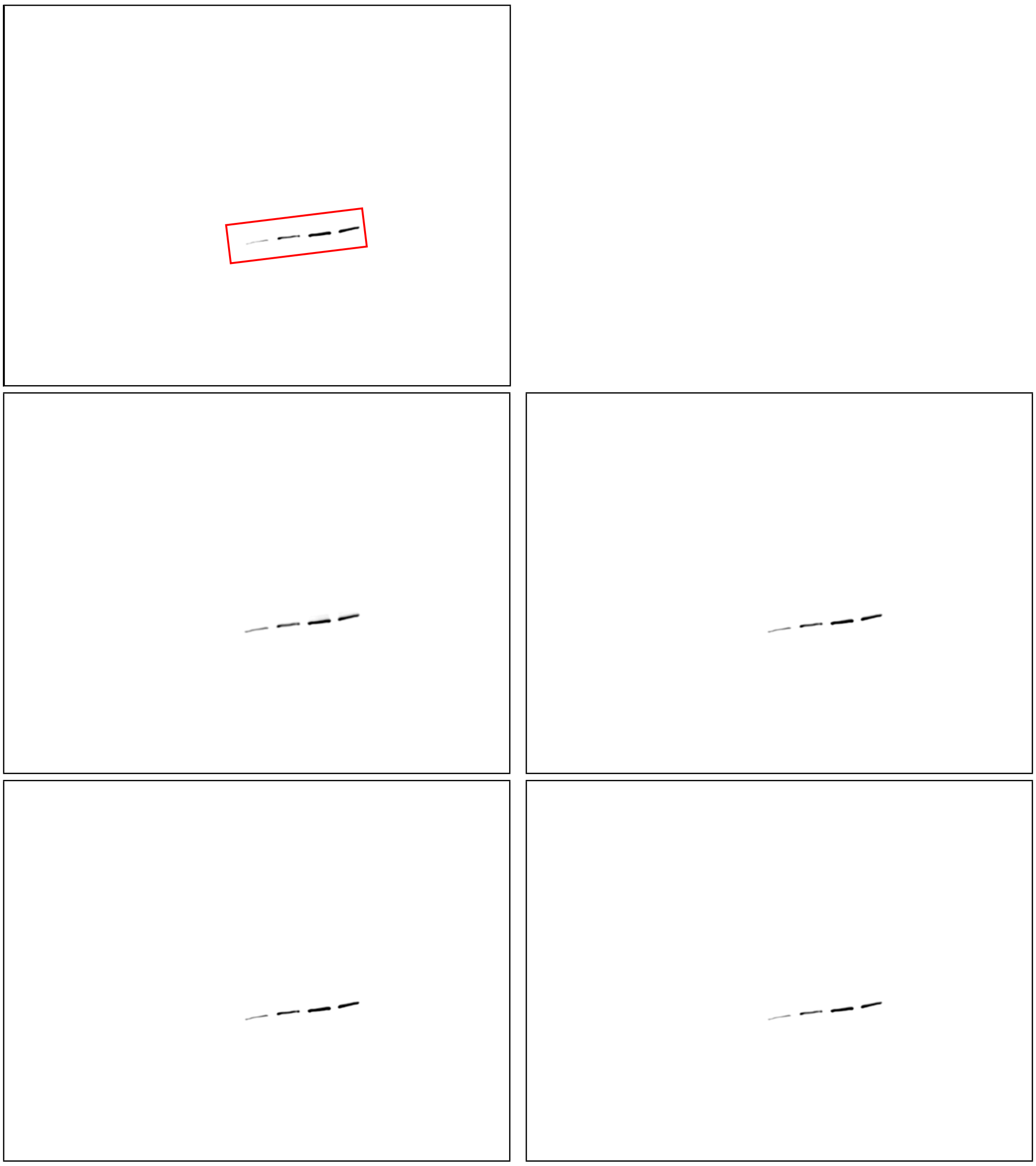

RelA

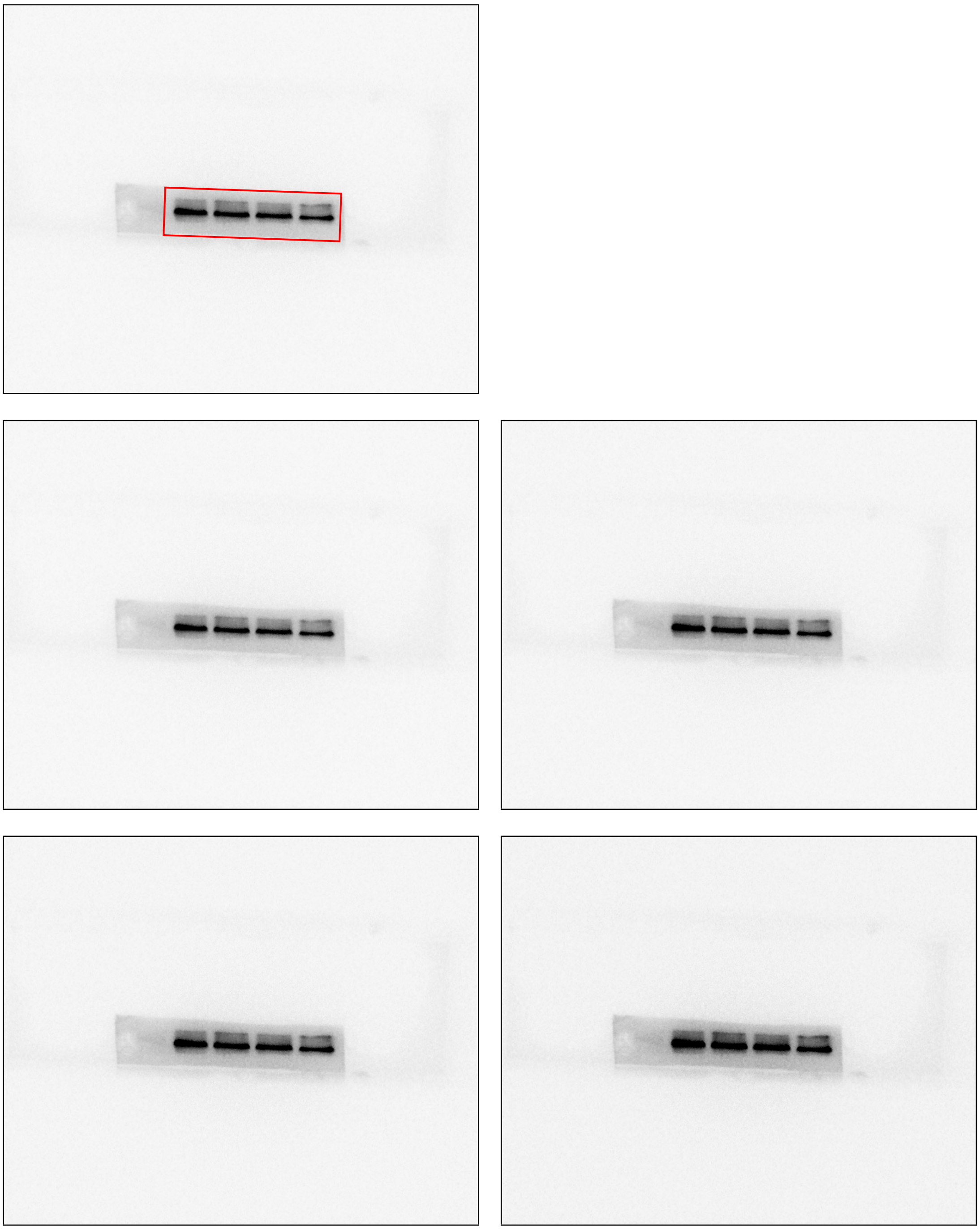

Figure 1D

HNF1α

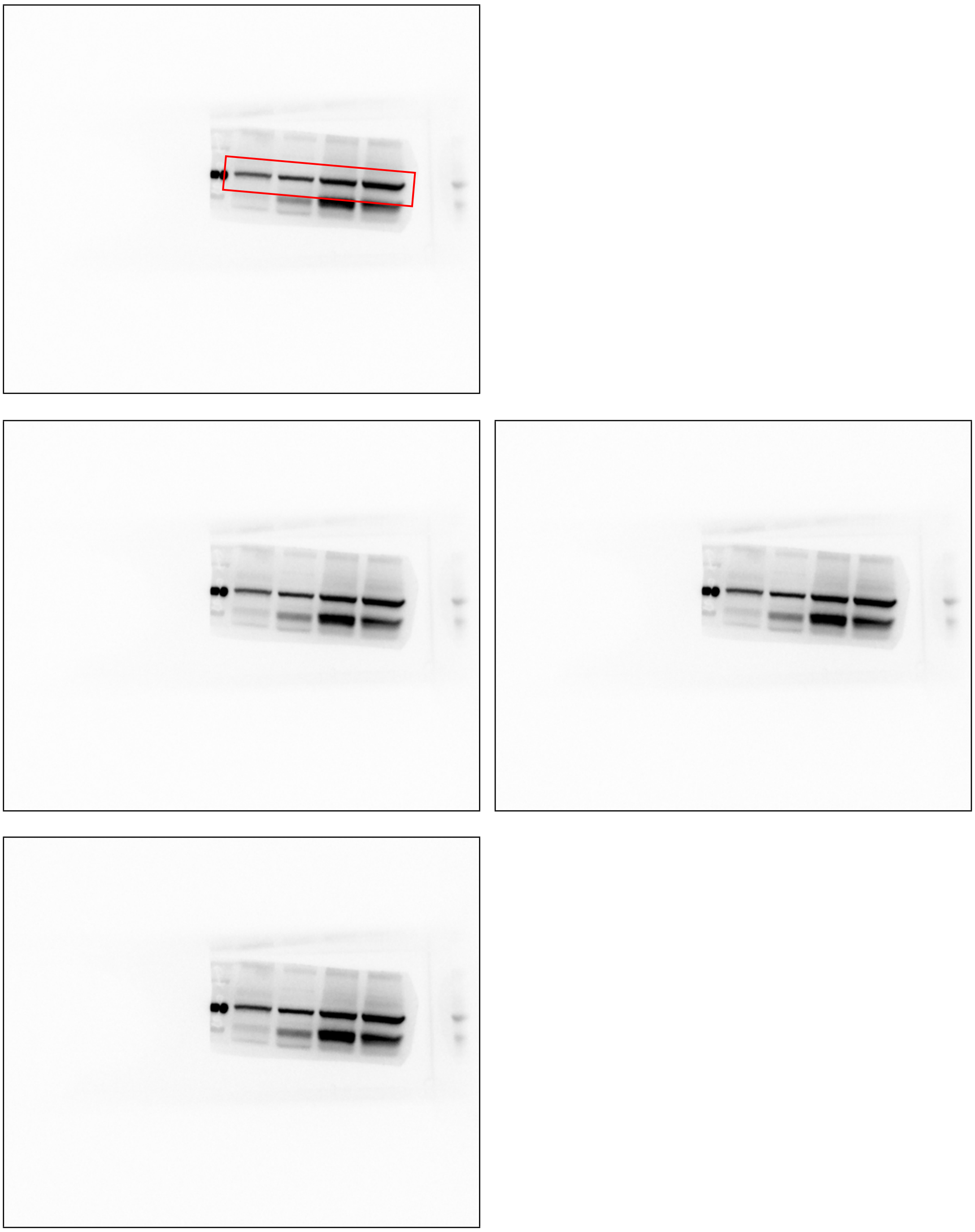

ATF4

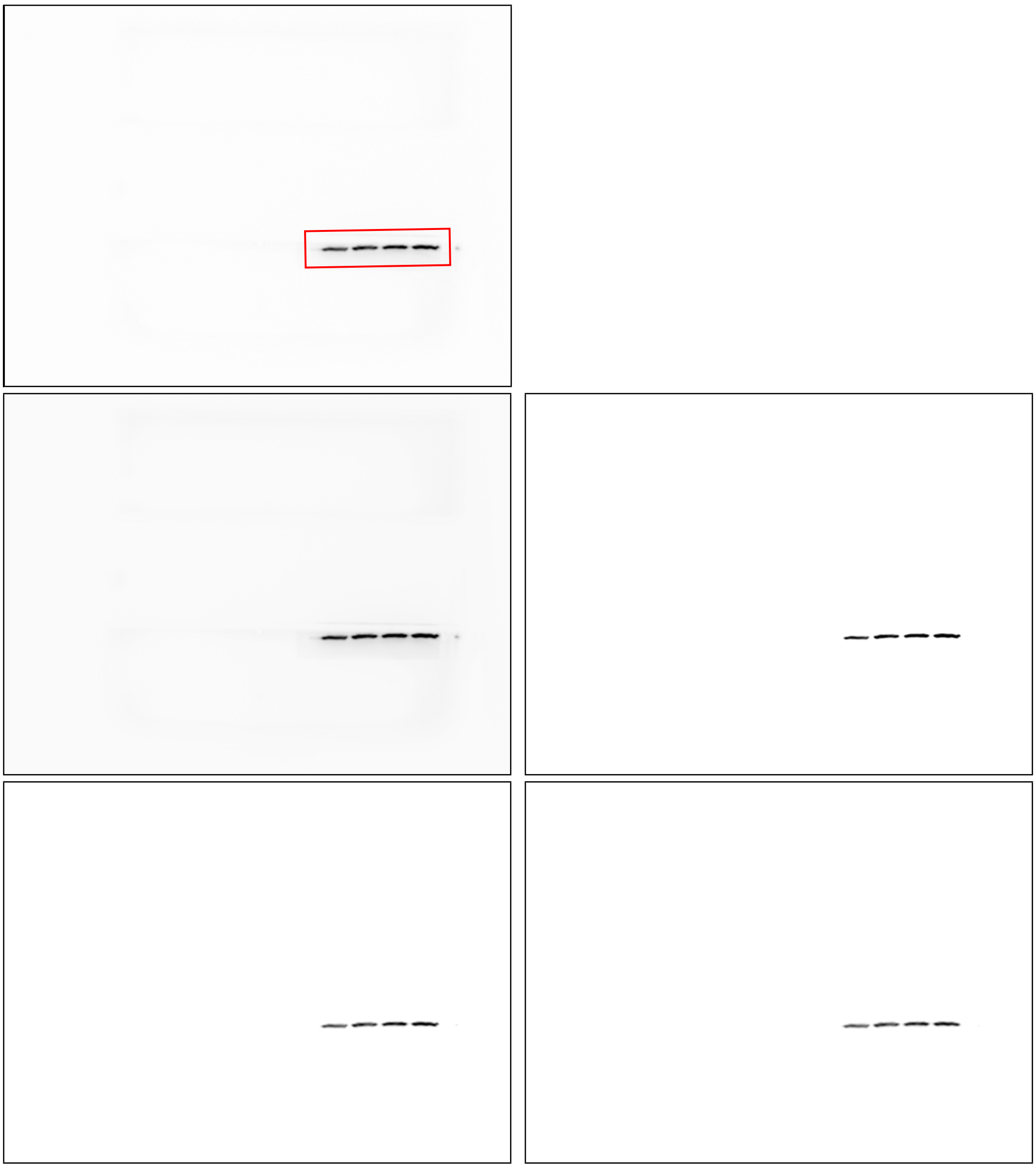

GRP78

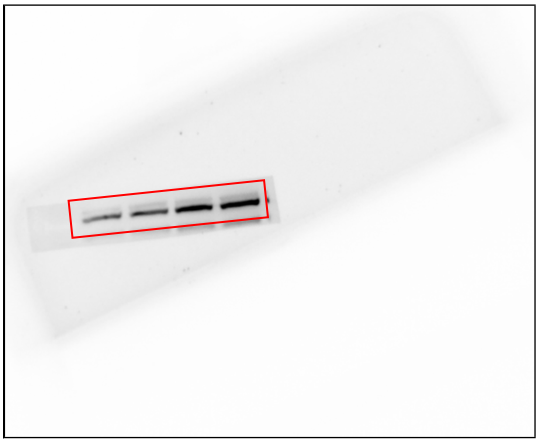

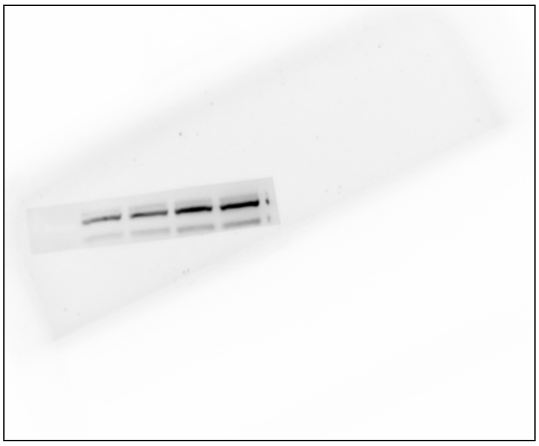

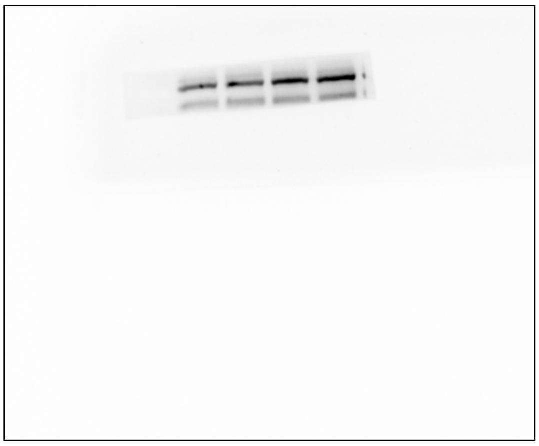

ATF6

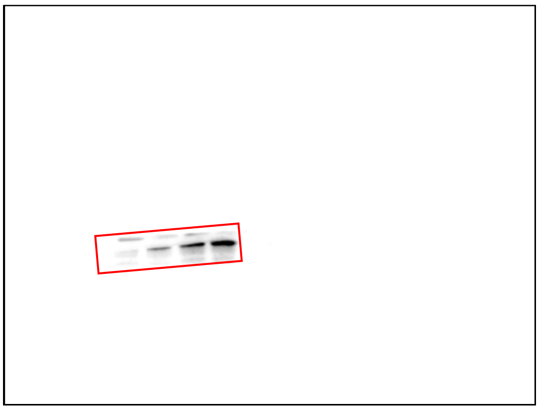

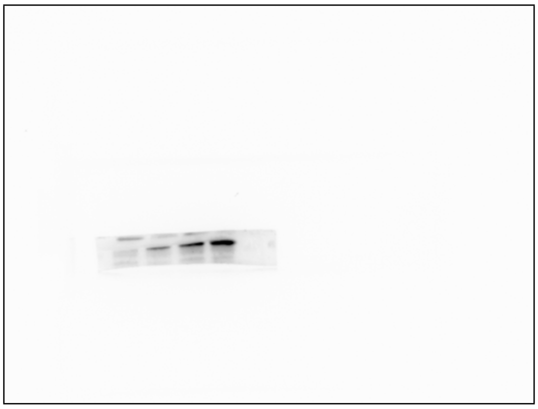

XBP1s

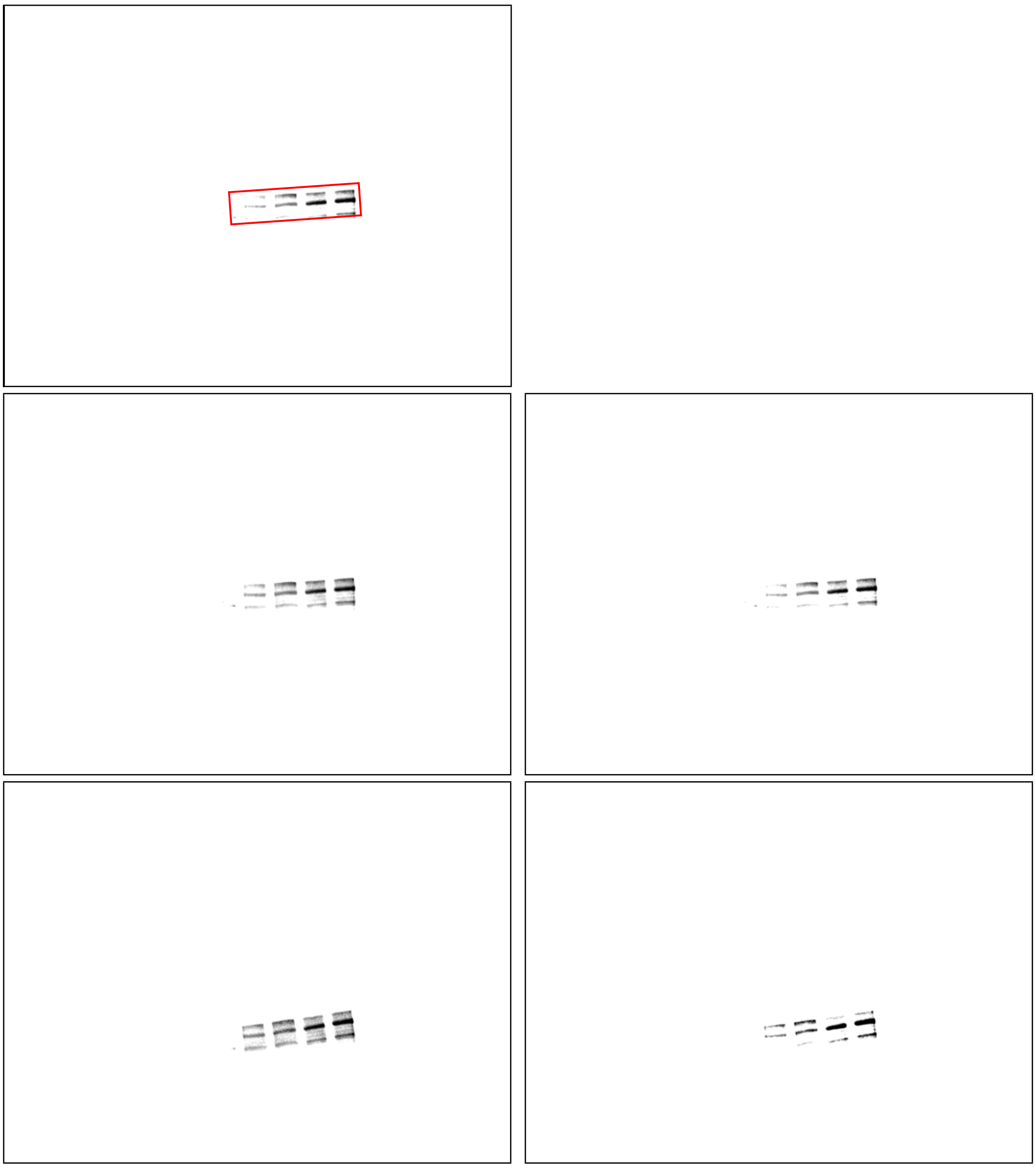

Cleaved caspase-3

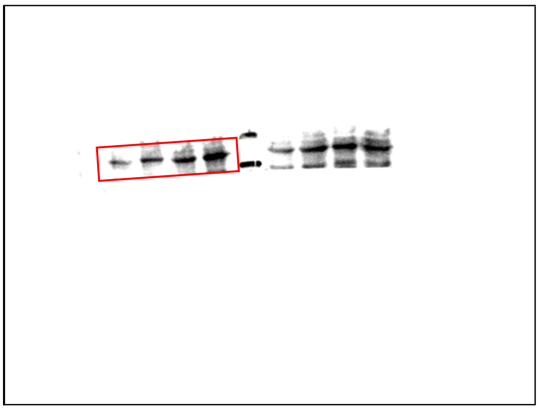

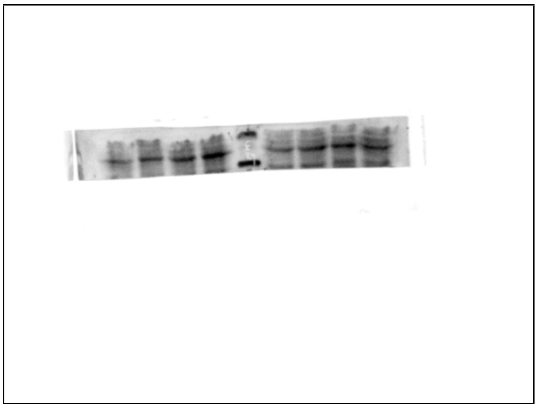

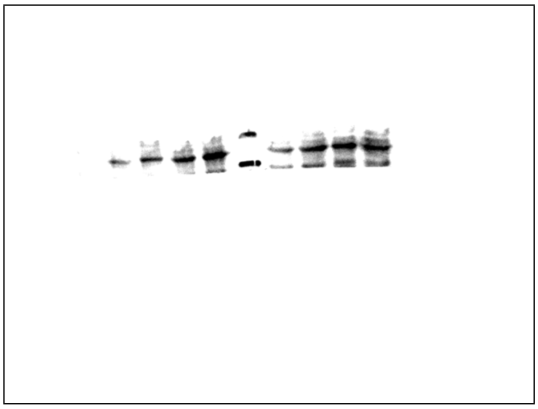

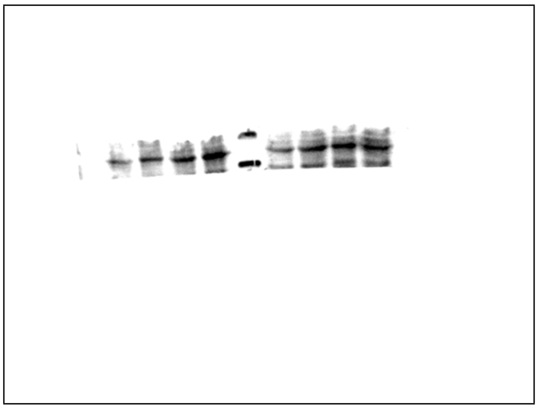

GAPDH

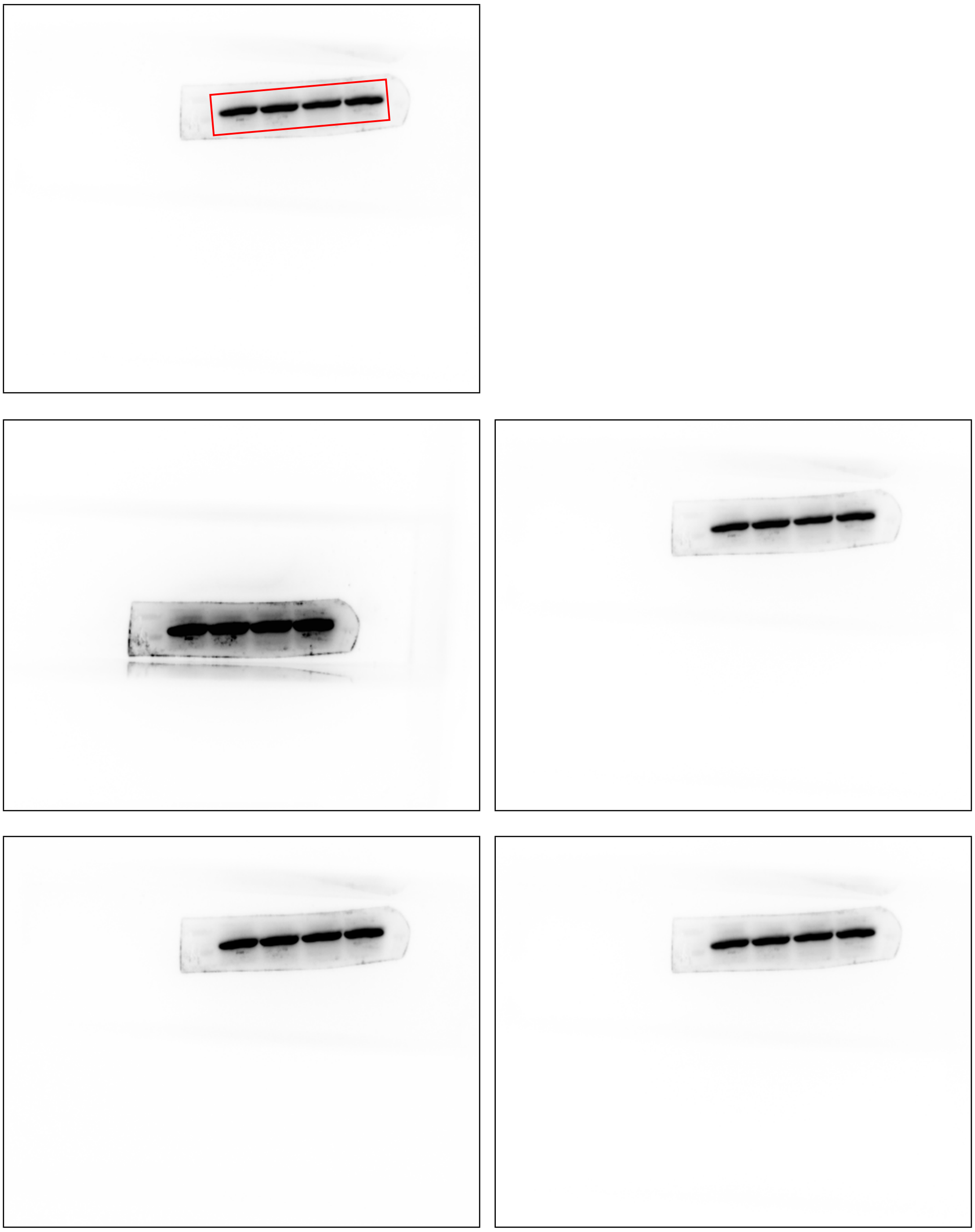

p-RelA

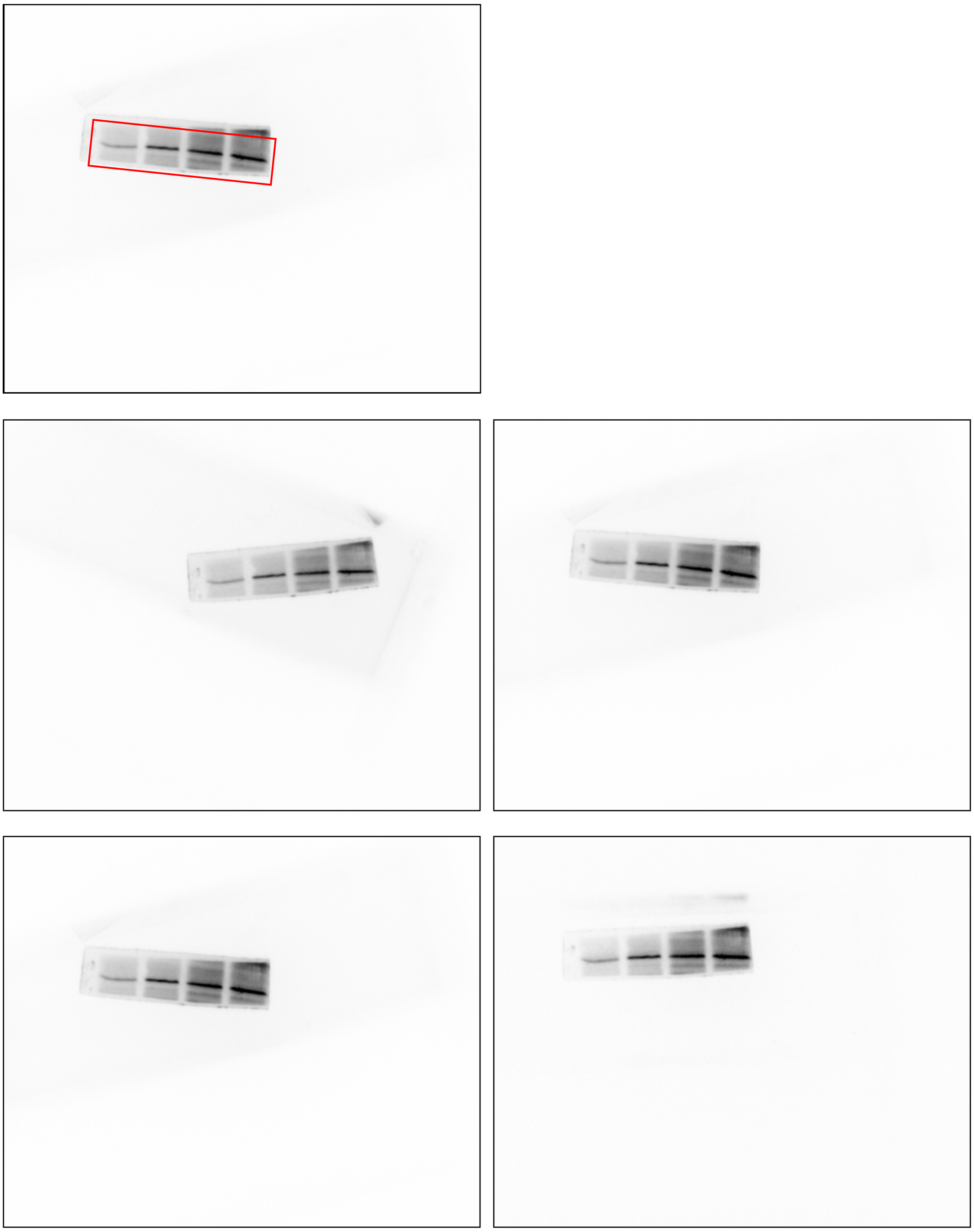

RelA

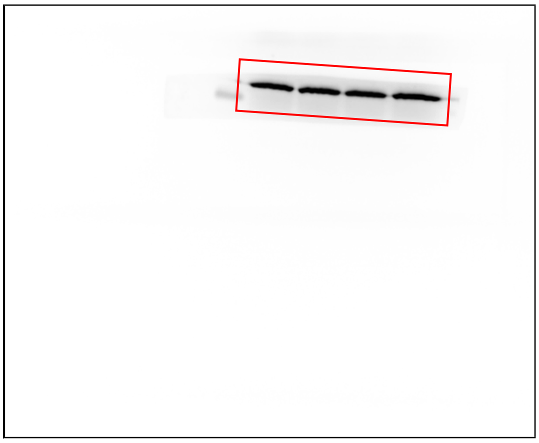

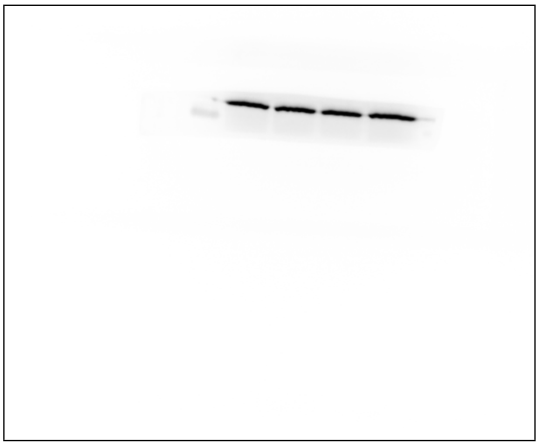

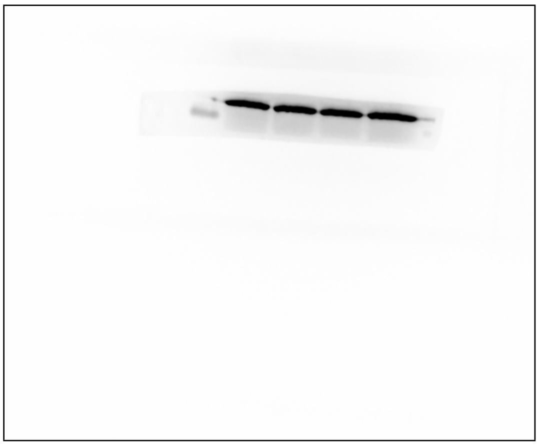

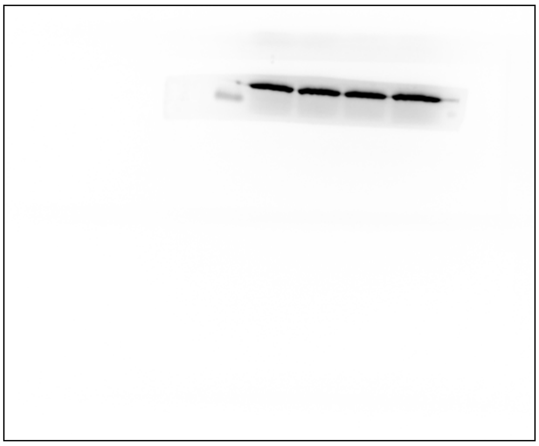

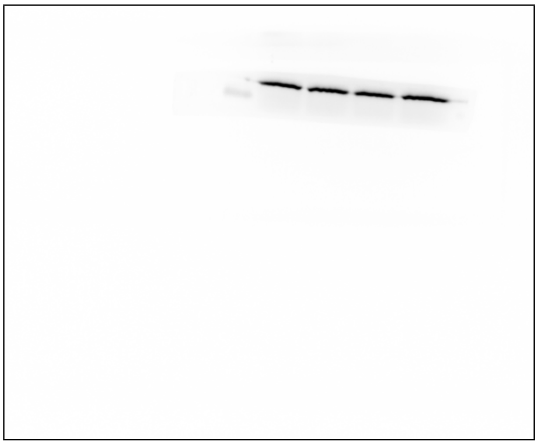

Figure 1F

HNF1α

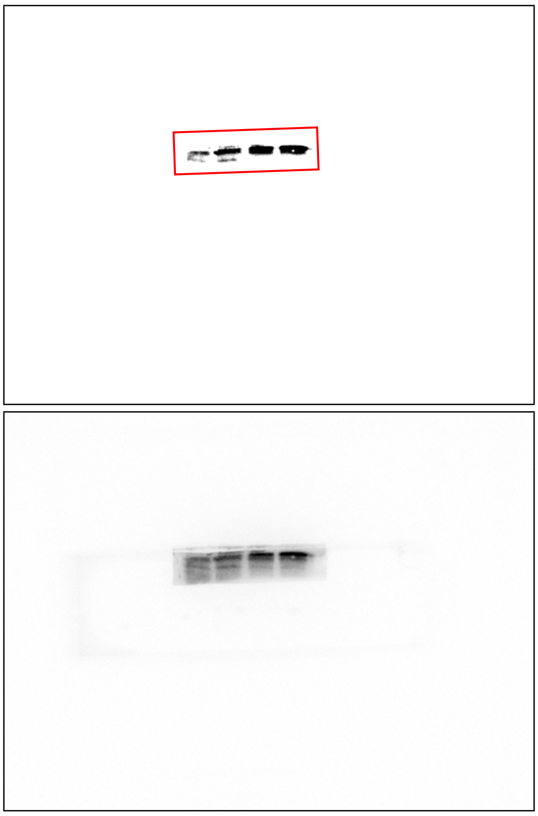

ATF4

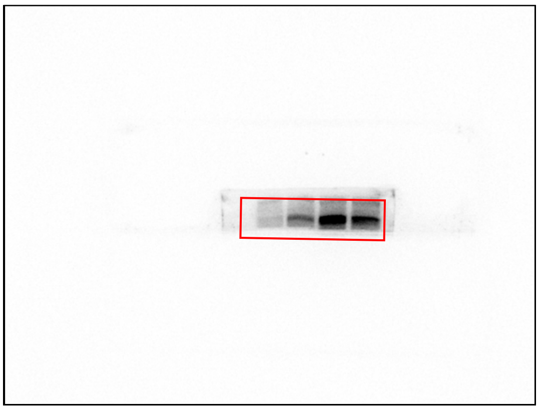

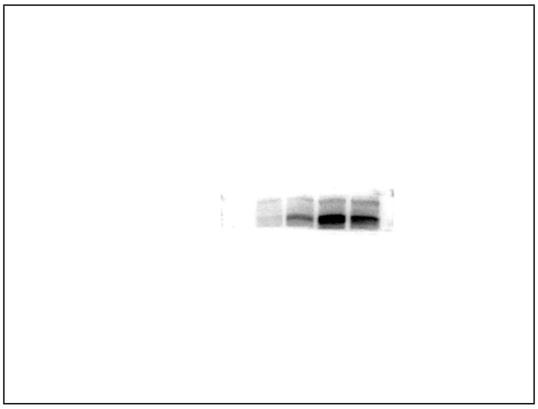

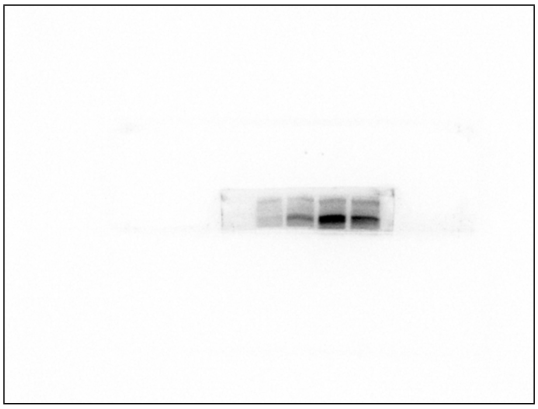

Cleaved caspase-3

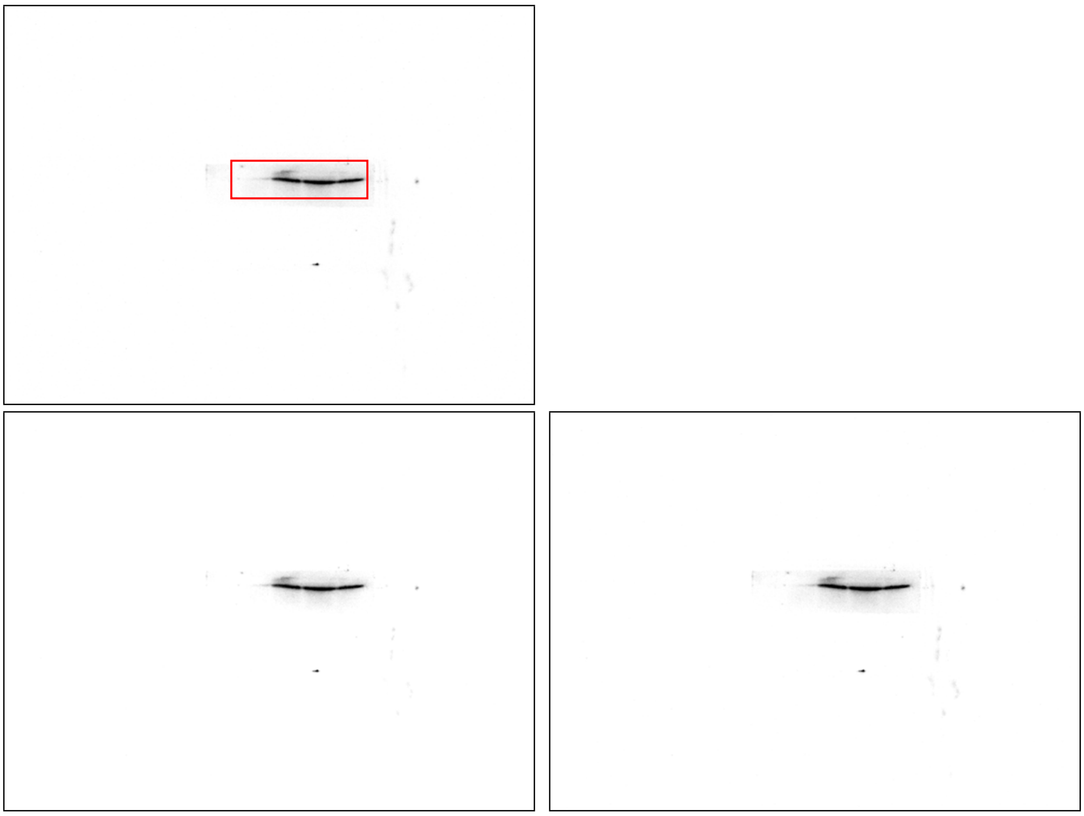

GAPDH

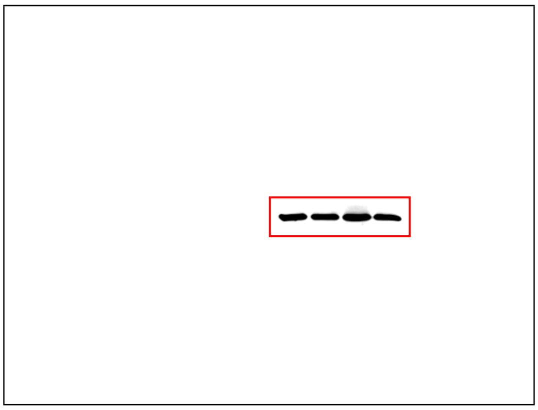

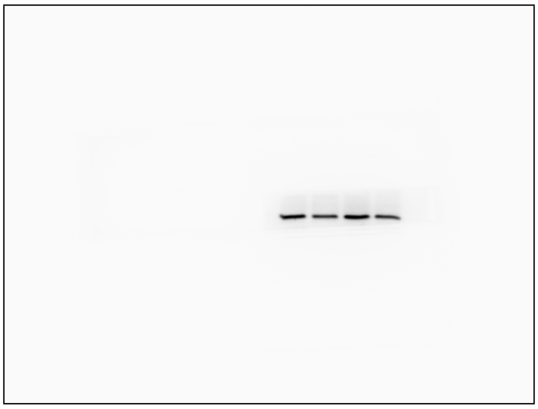

p-RelA

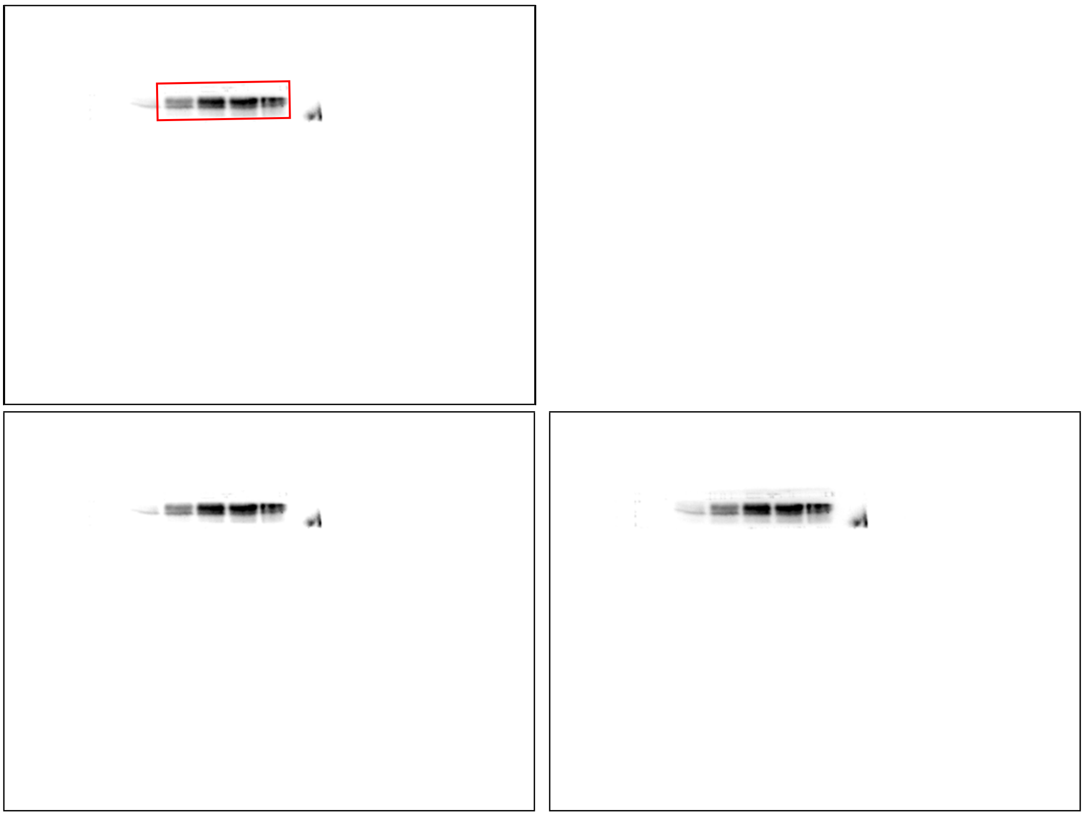

RelA

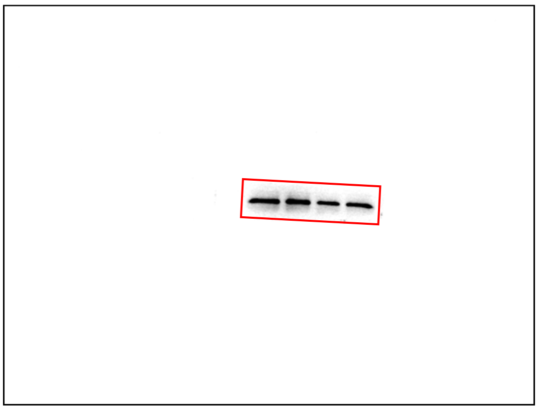

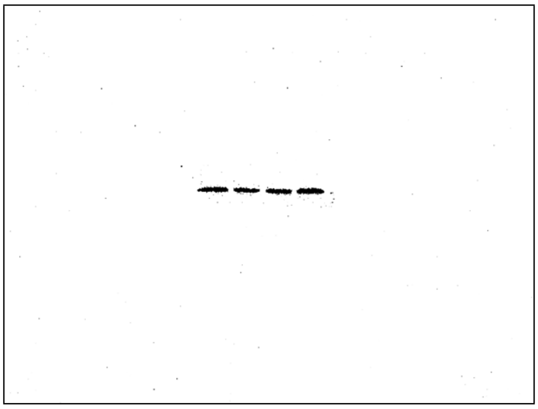

Figure 1H

HNF1α

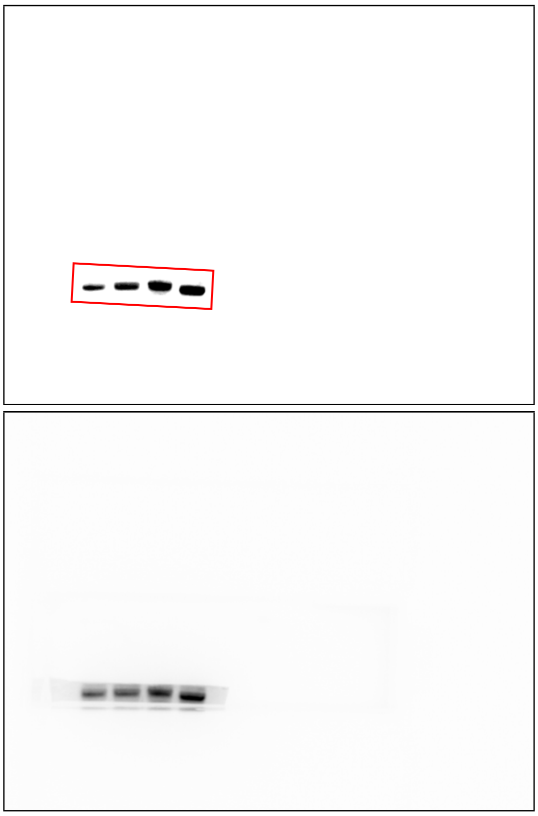

ATF4

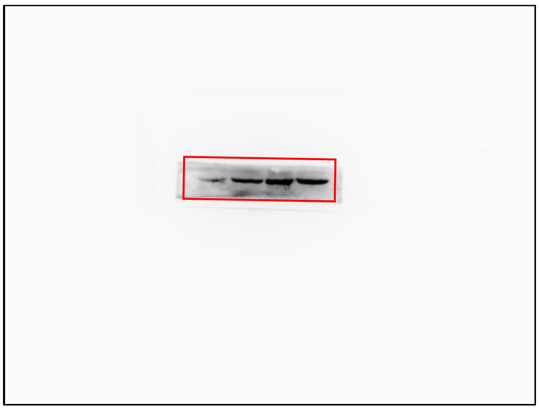

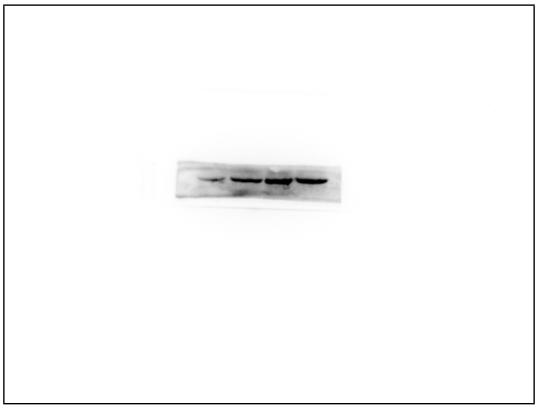

Cleaved caspase-3

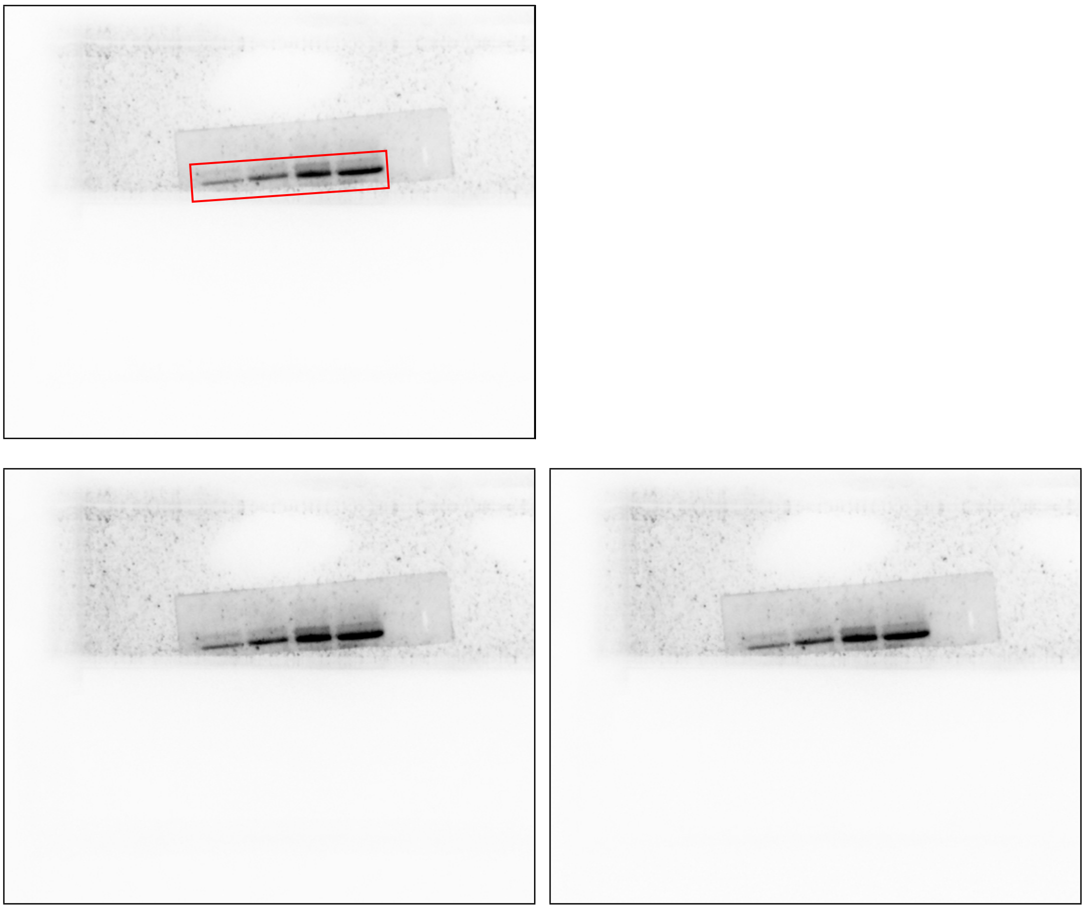

GAPDH

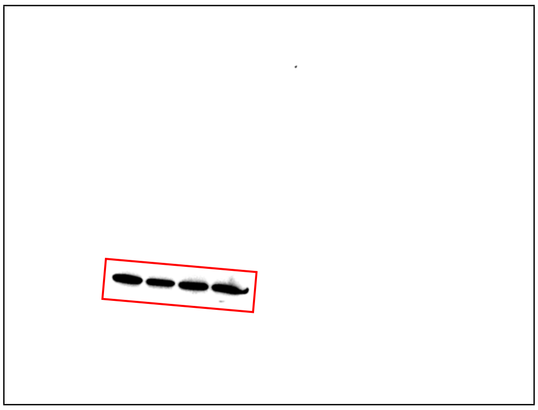

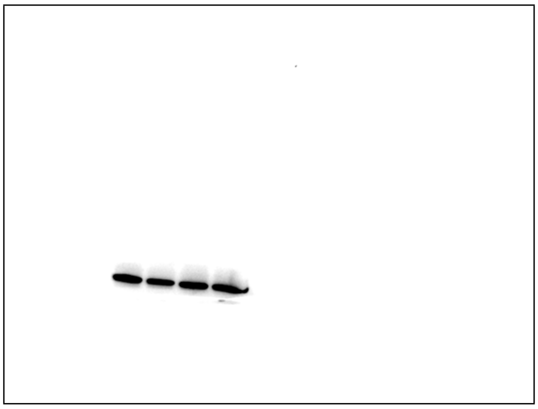

p-RelA

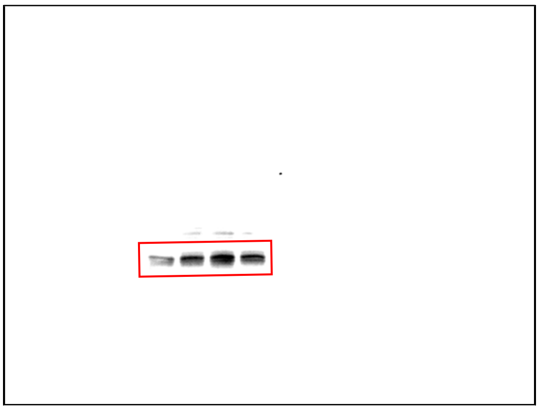

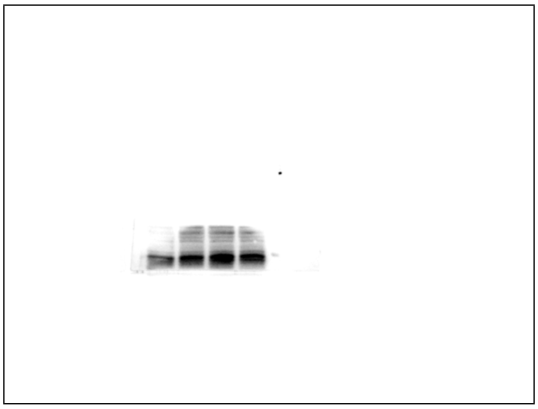

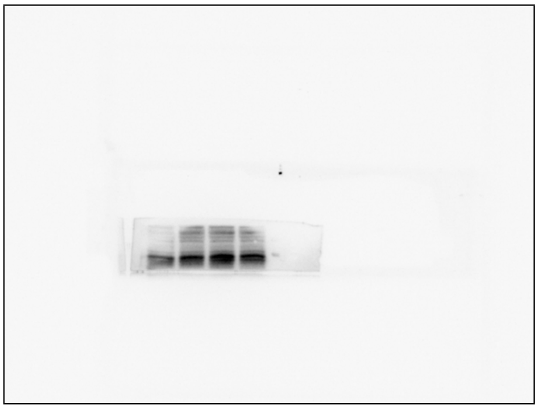

RelA

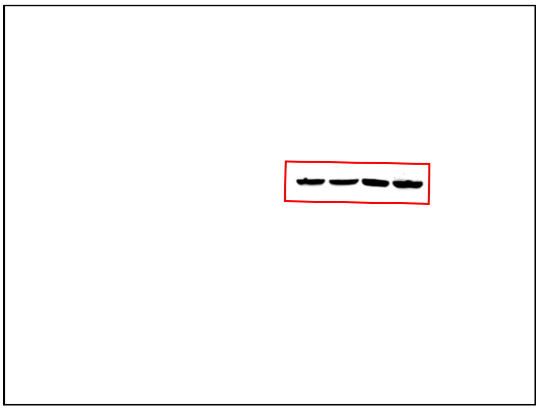

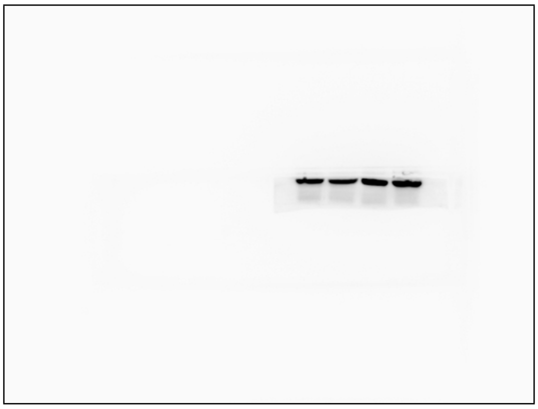

Figure 1J

HNF1α

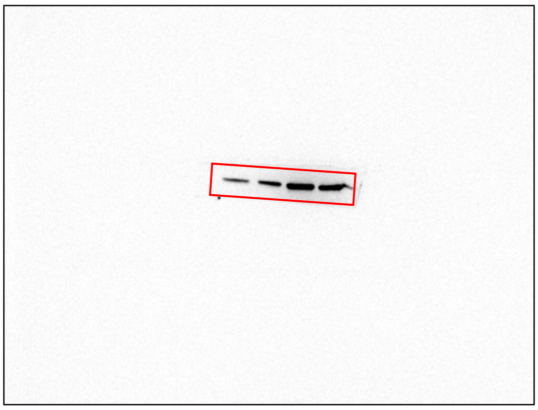

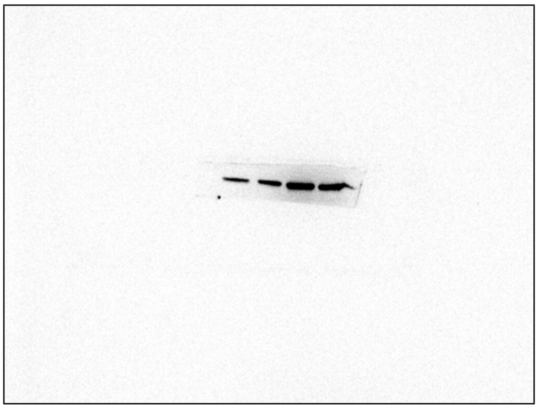

ATF4

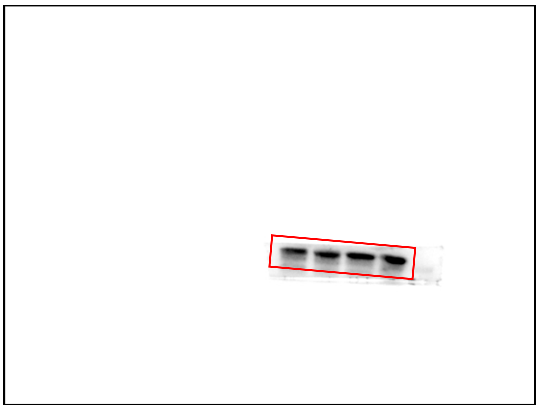

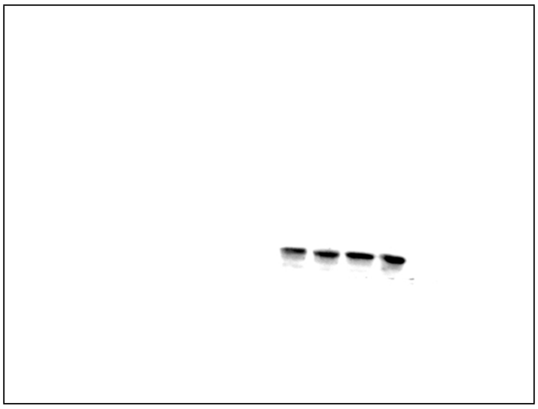

Cleaved caspase-3

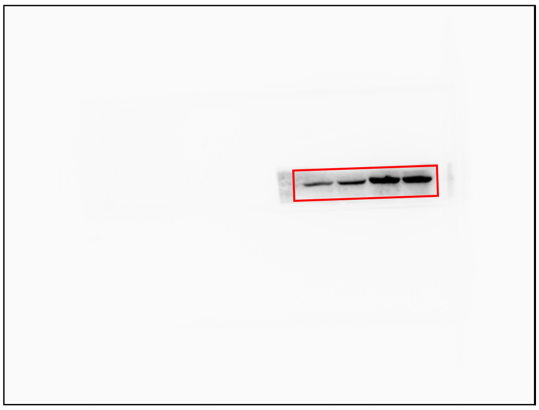

GAPDH

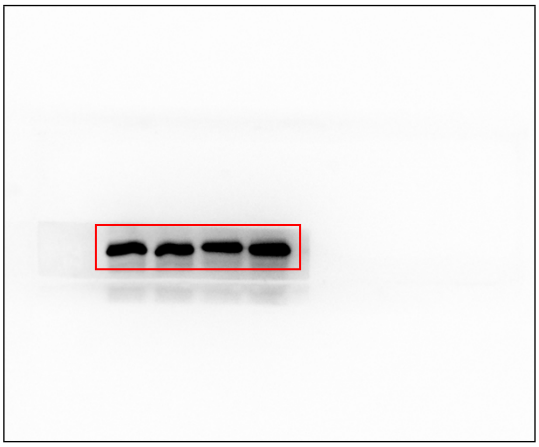

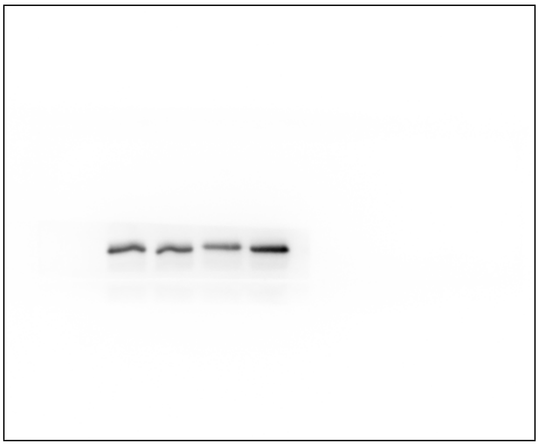

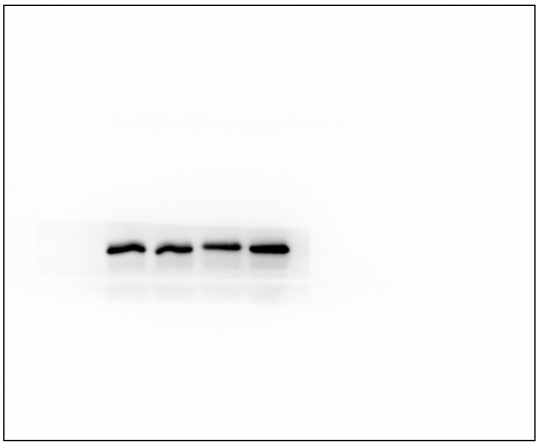

p-RelA

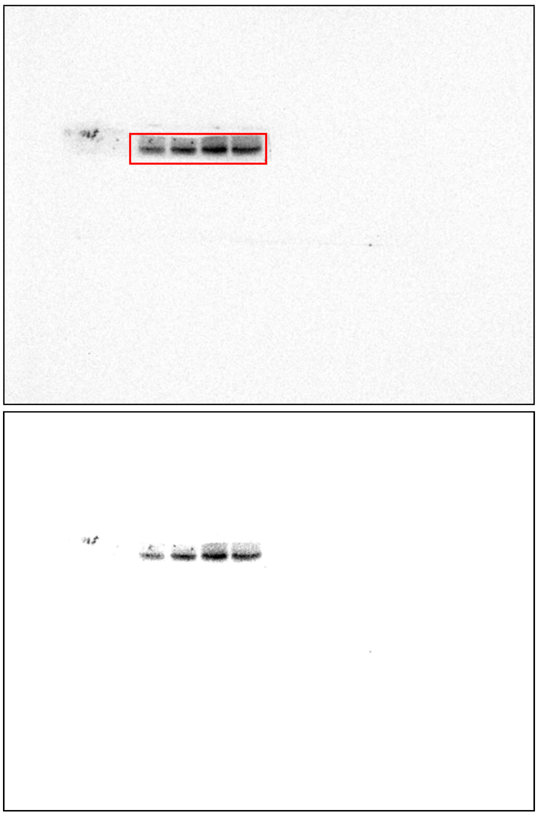

RelA

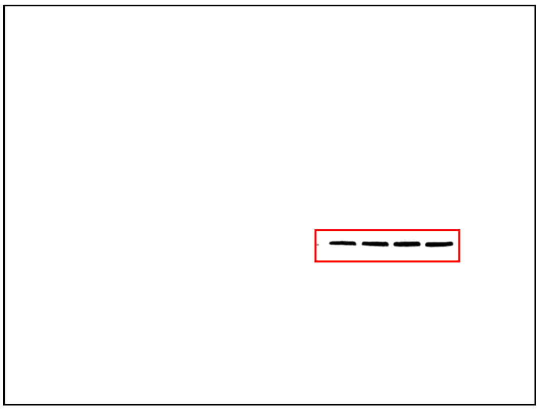

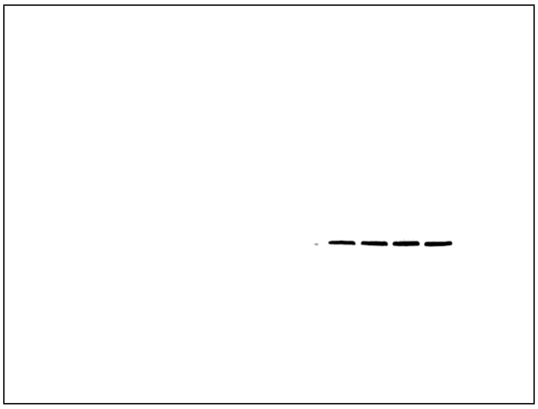

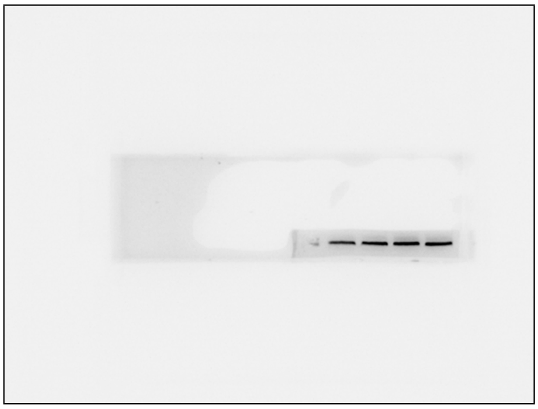

Figure 2A

HNF1α

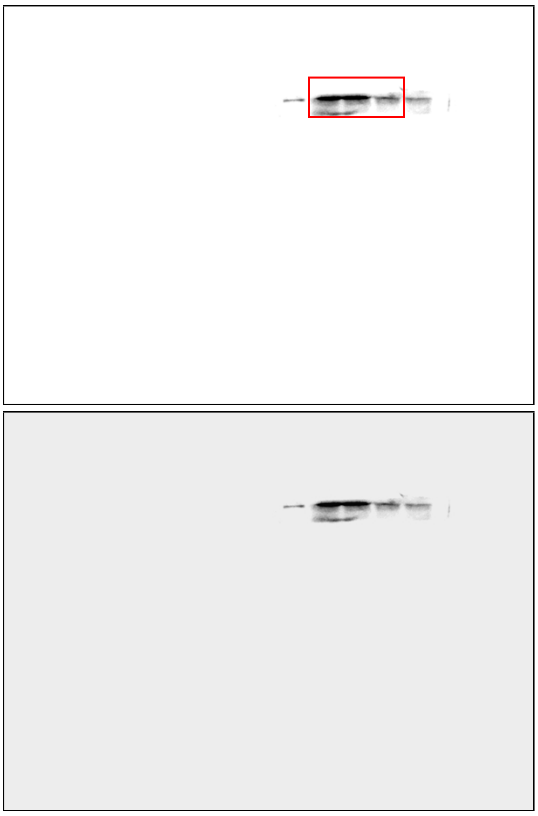

GAPDH

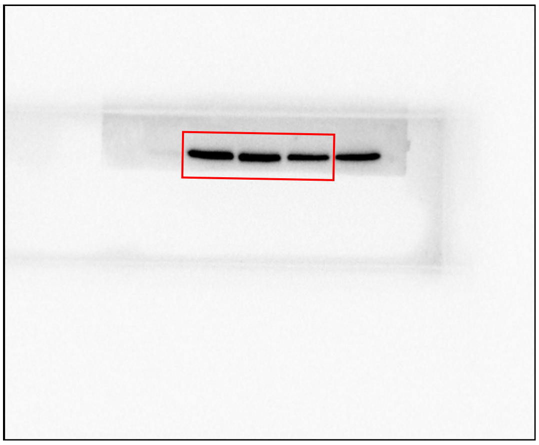

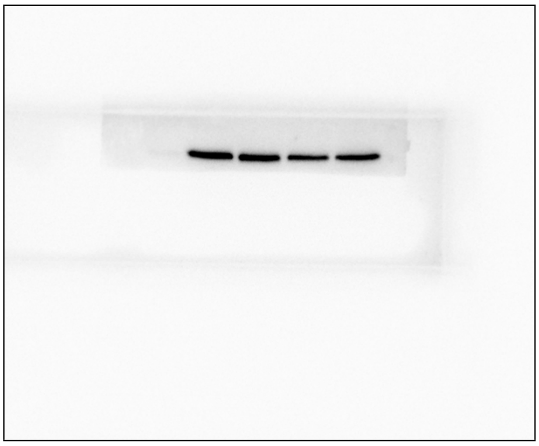

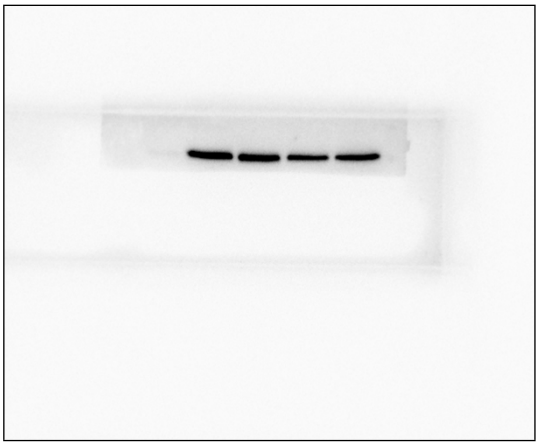

Figure 2C

HNF1α

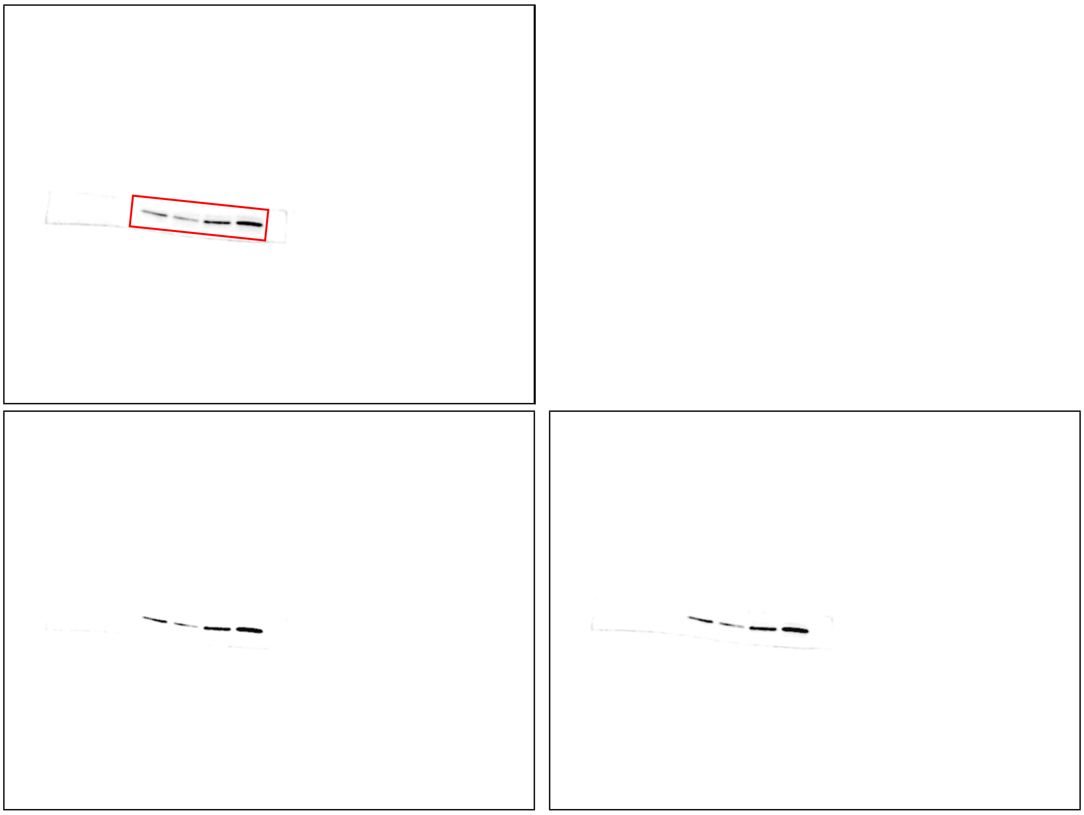

ATF4

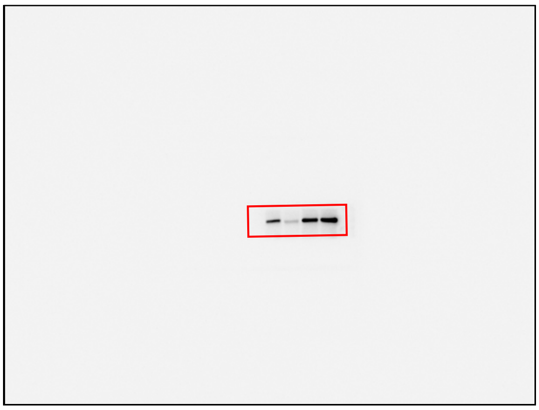

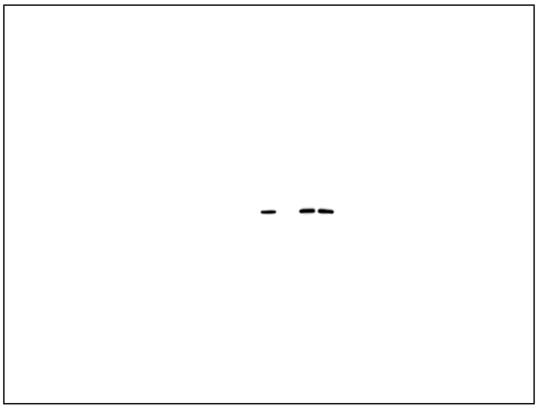

GRP78

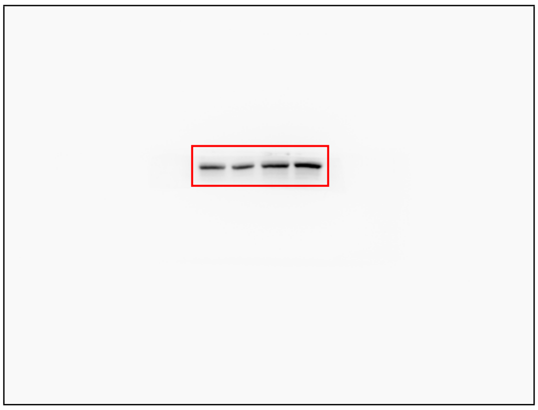

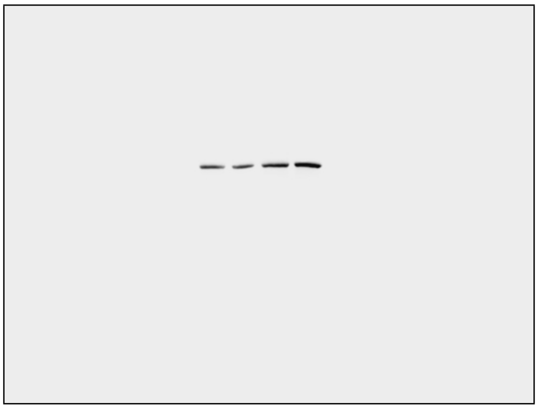

ATF6

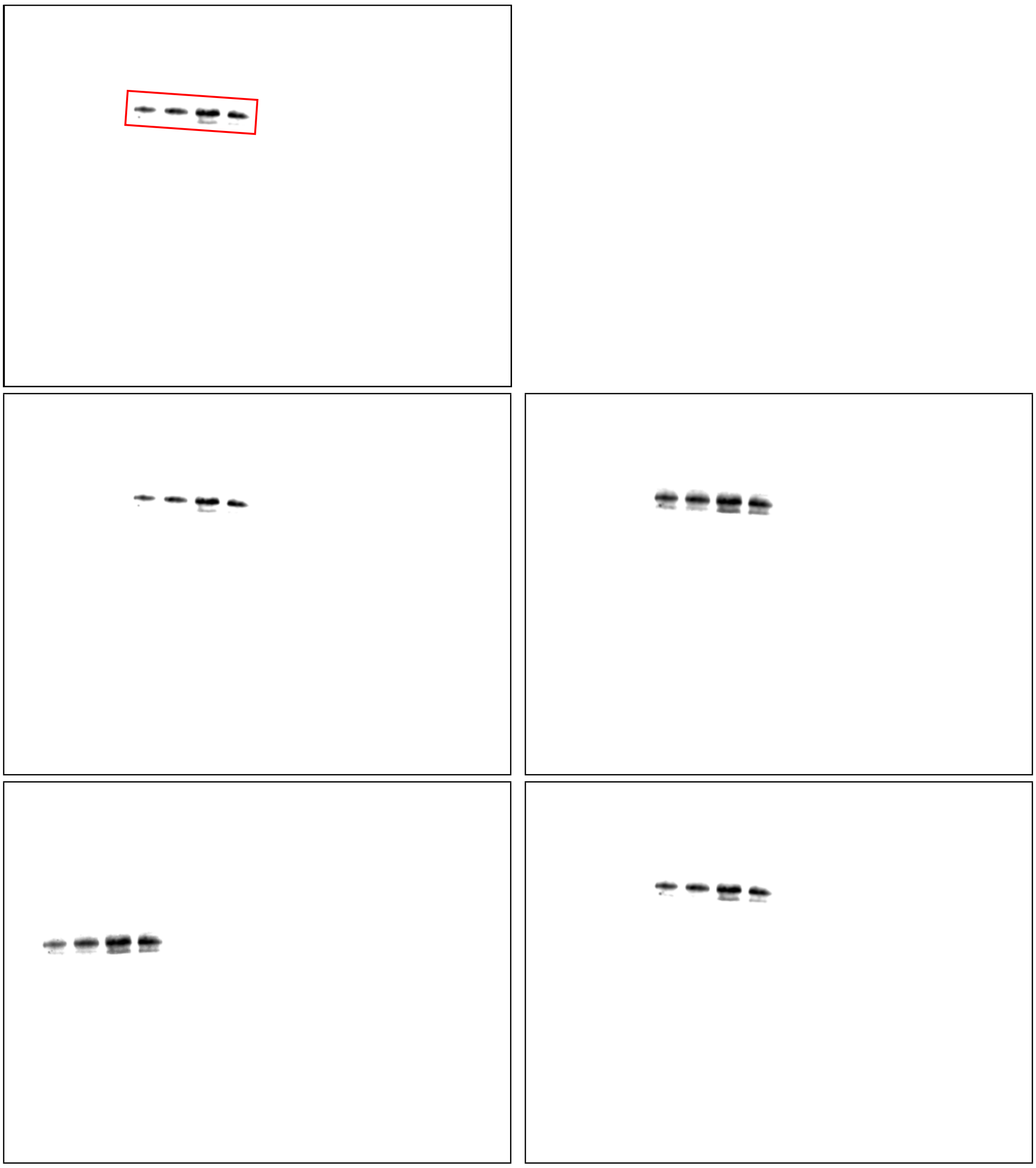

XBP1s

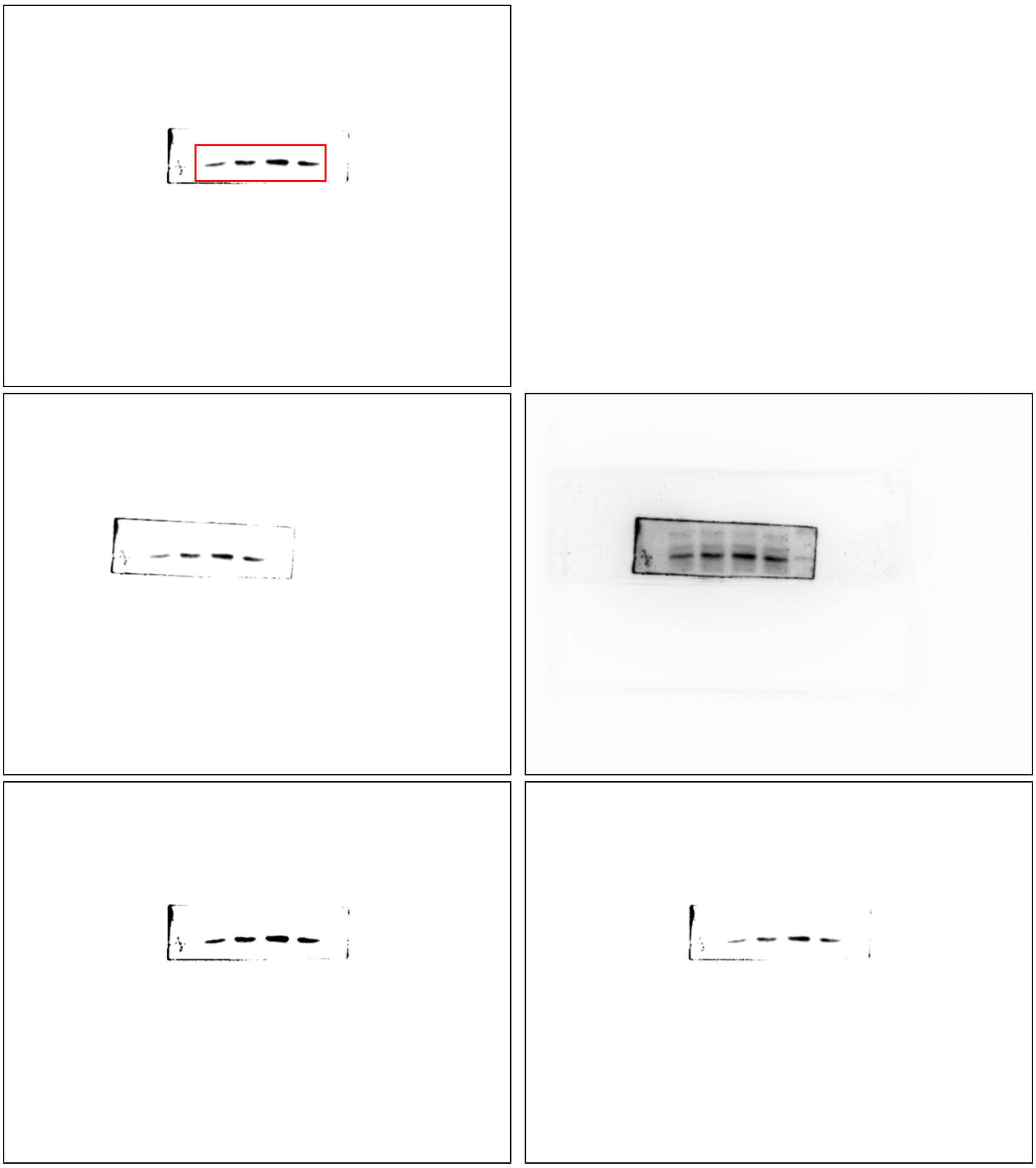

Cleaved caspase-3

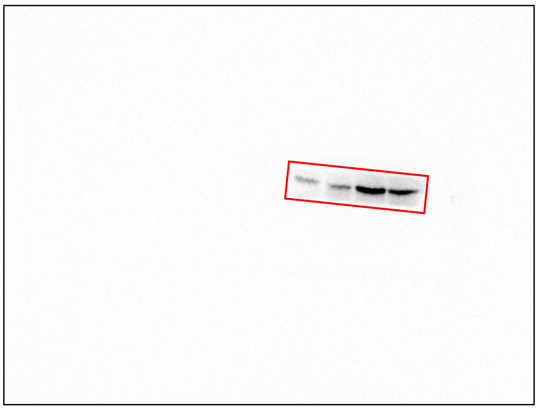

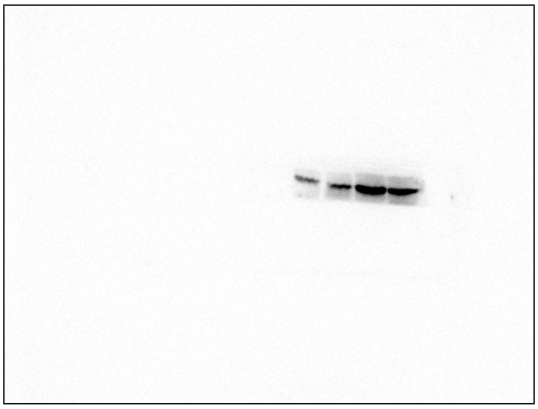

GAPDH

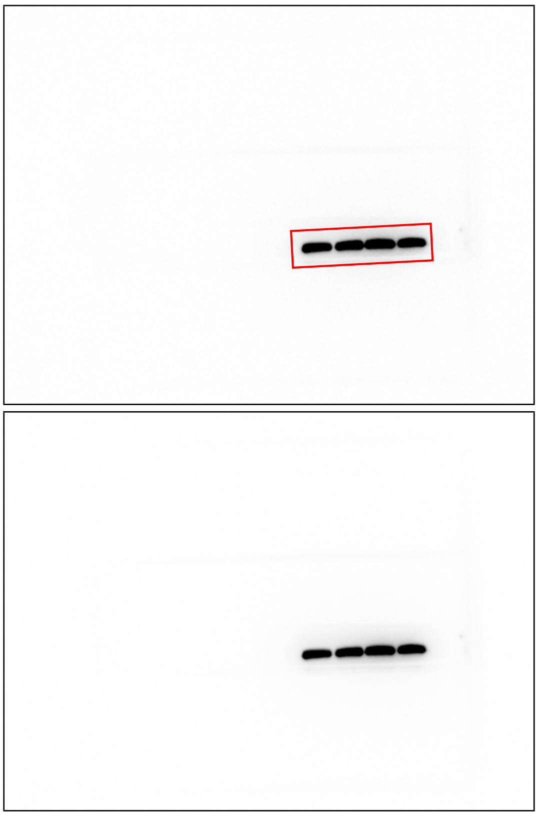

p-RelA

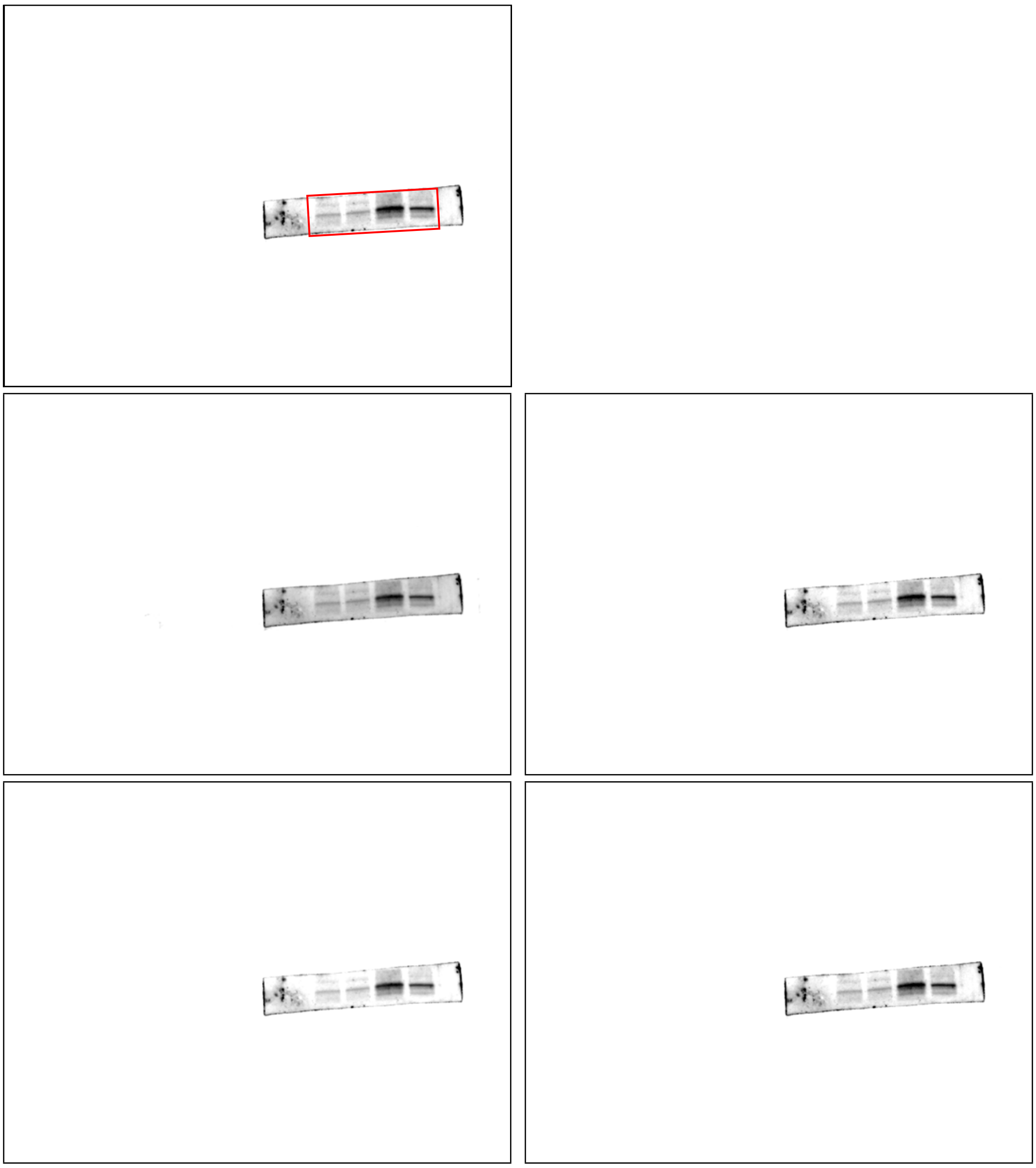

RelA

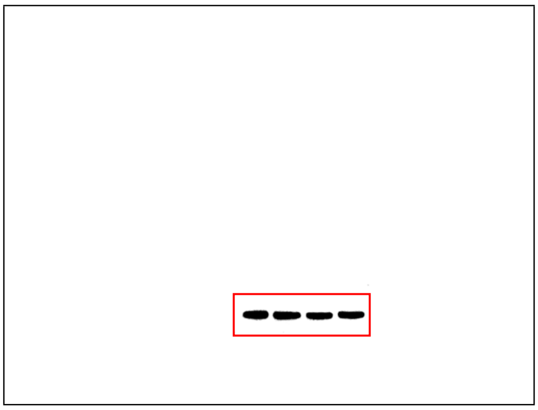

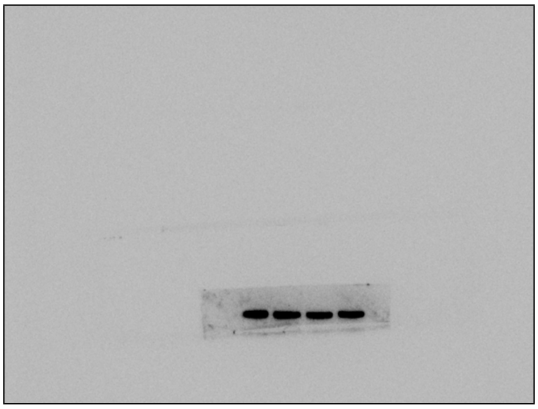

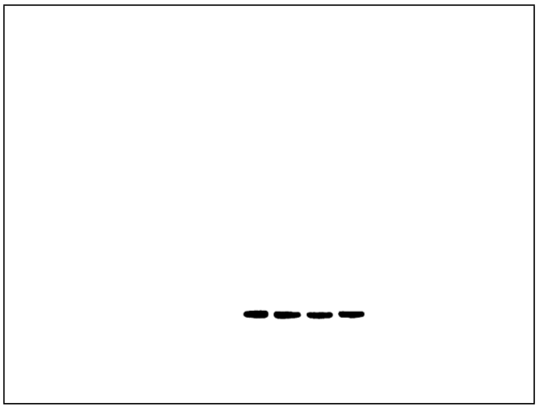

Figure 3A

ATF4

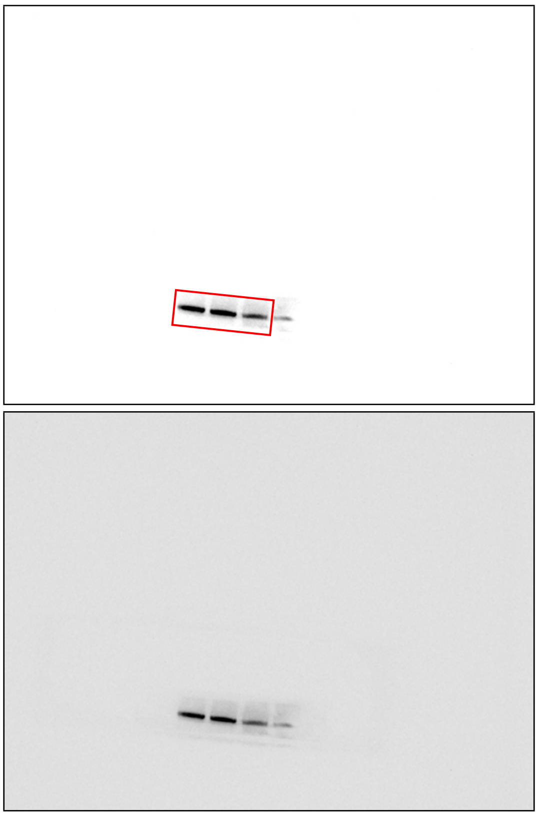

GAPDH

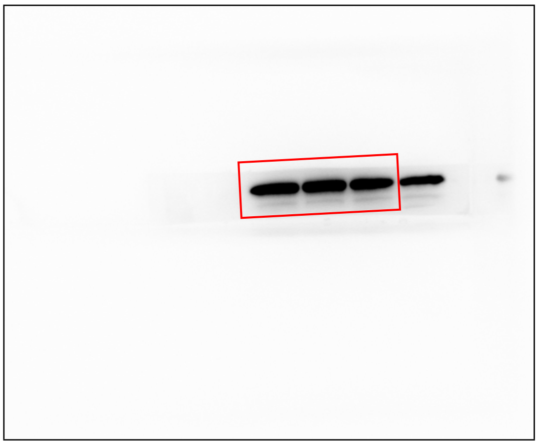

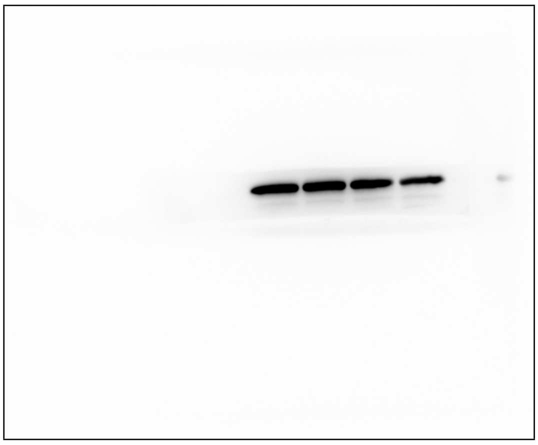

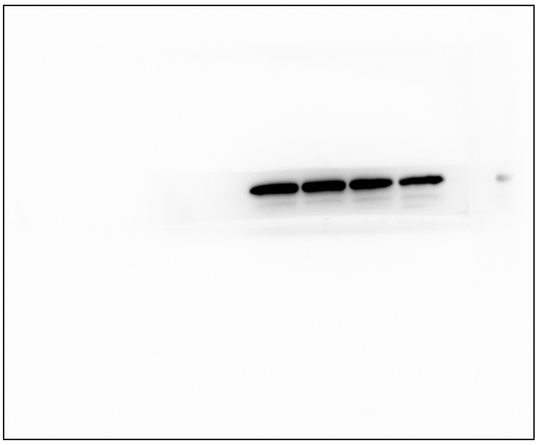

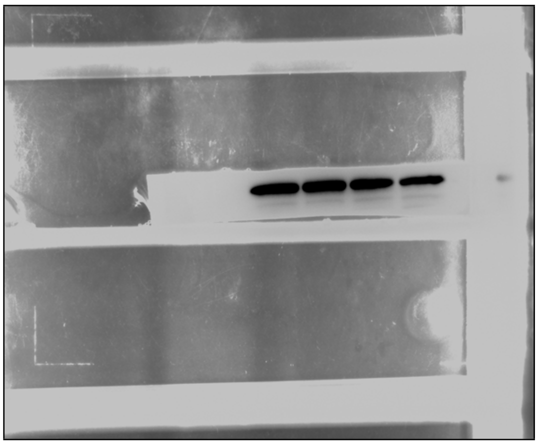

Figure 3C

ATF4

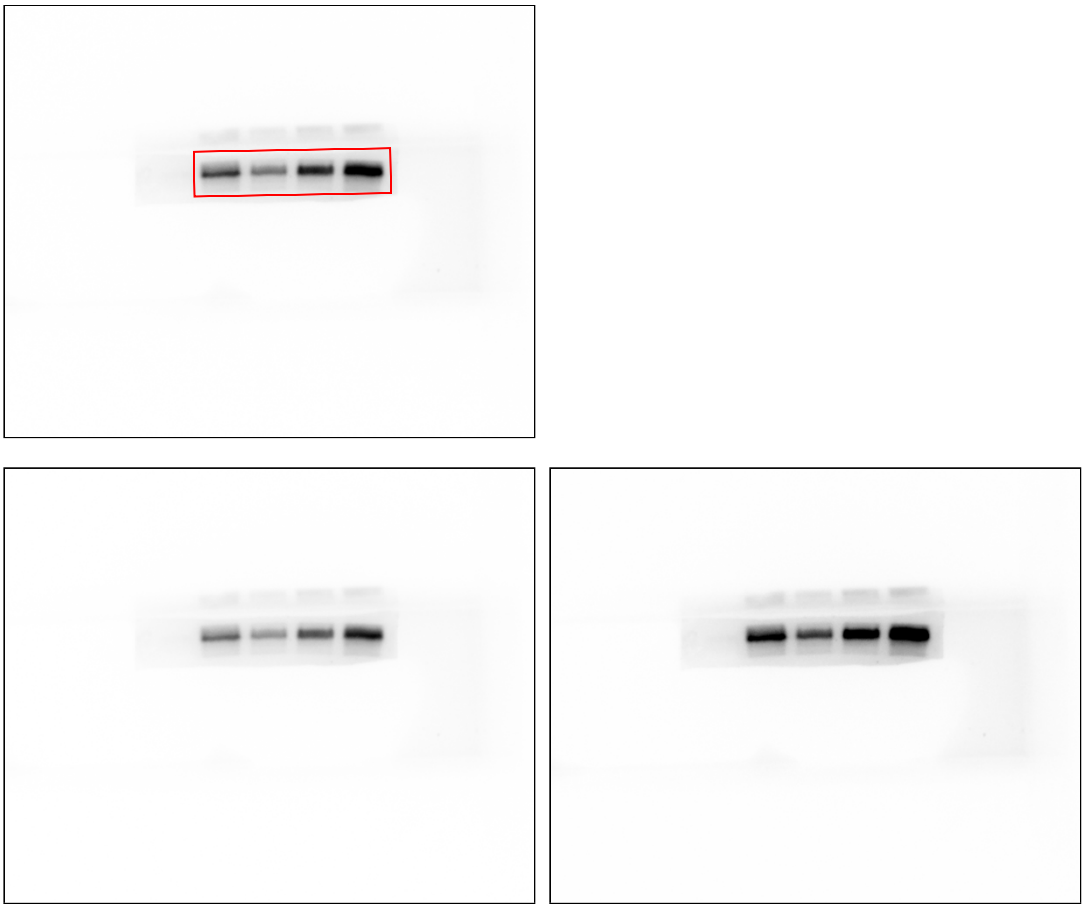

GRP78

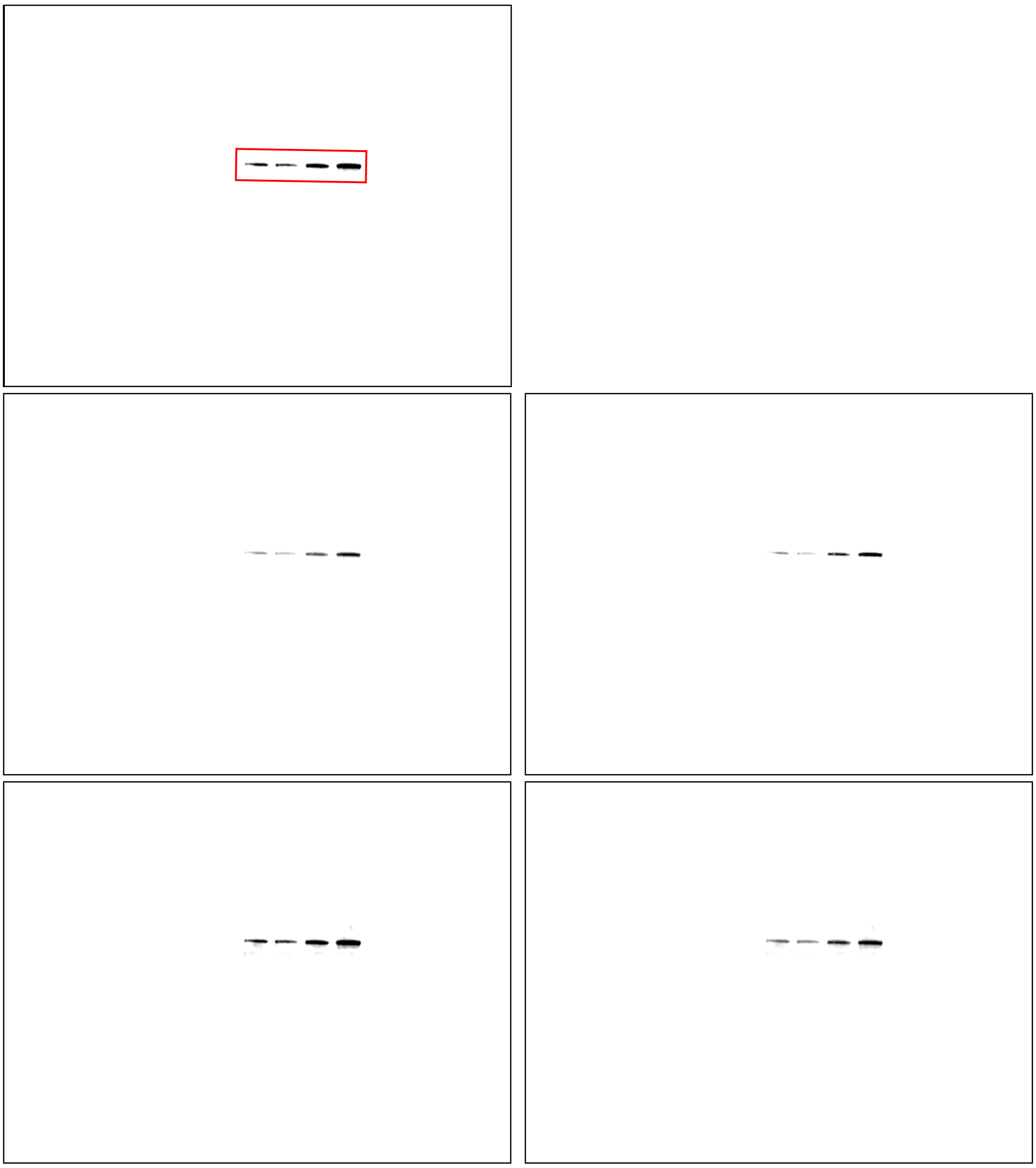

Cleaved caspase-3

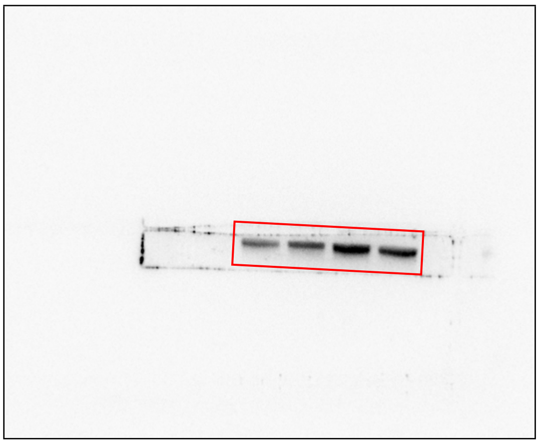

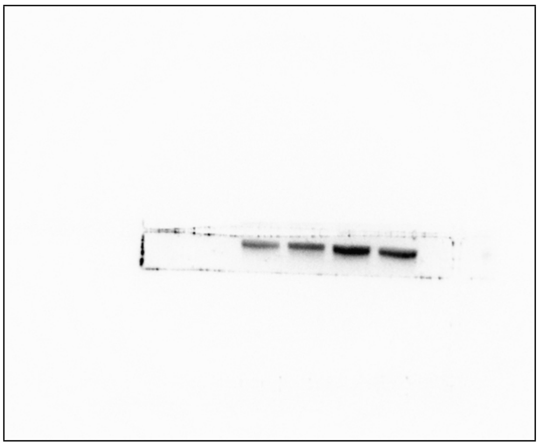

HNF1α

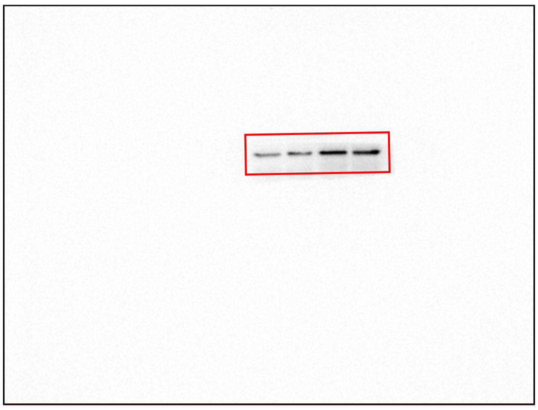

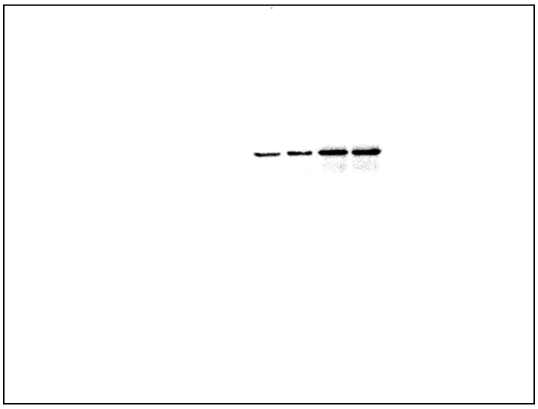

GAPDH

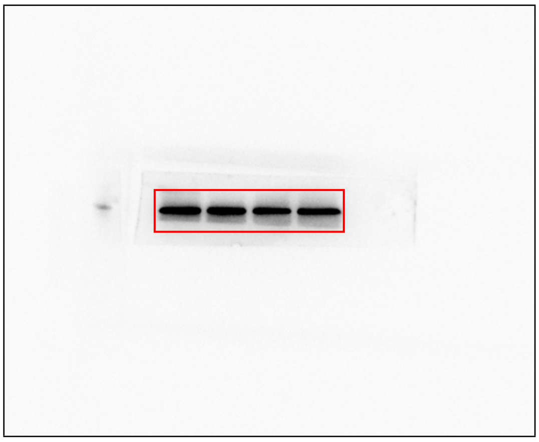

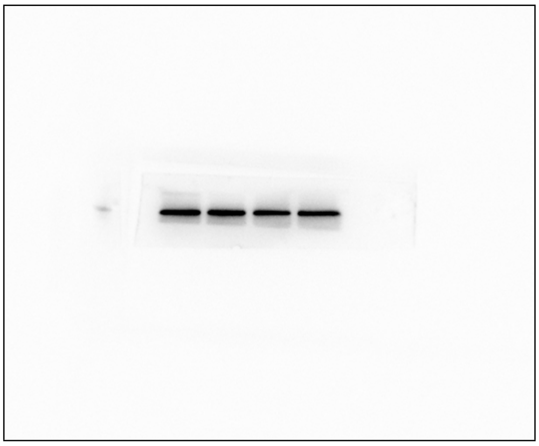

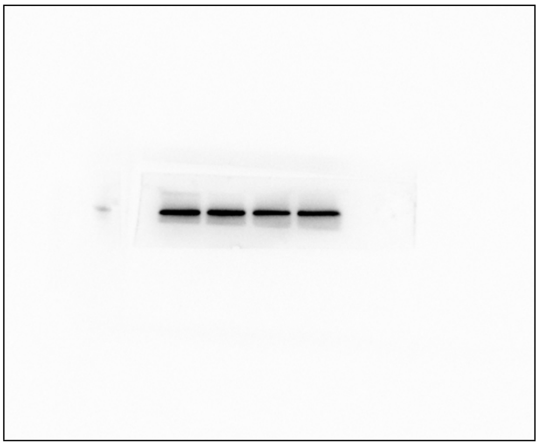

p-RelA

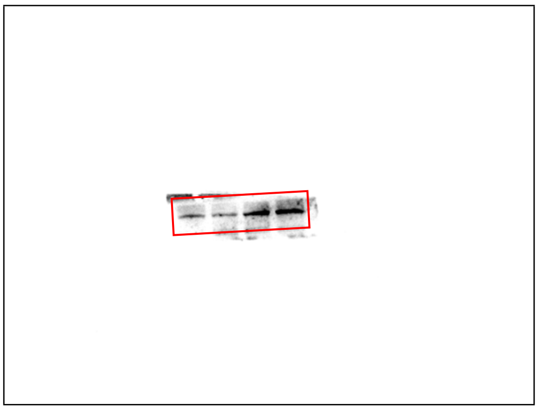

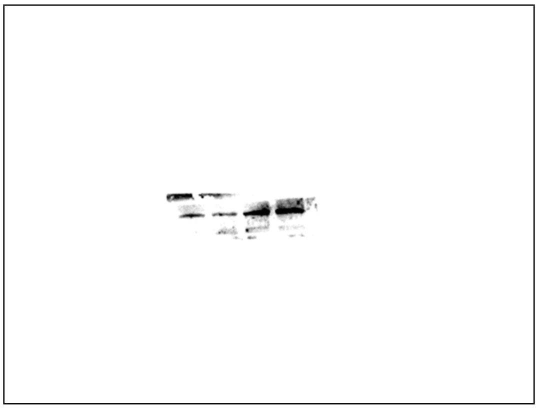

RelA

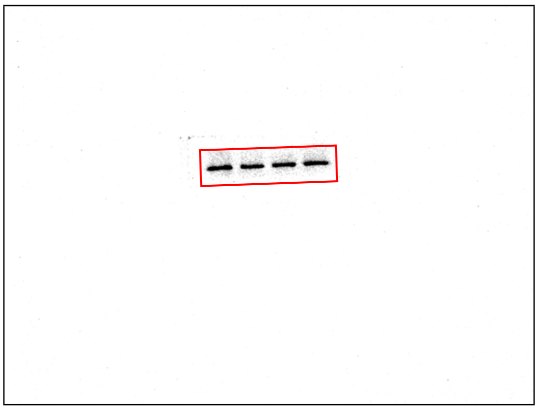

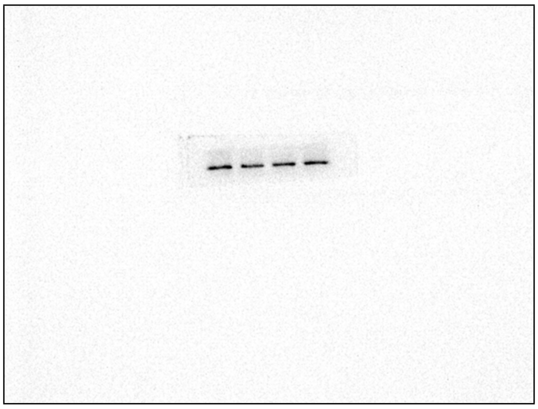

Figure 4A

ATF6

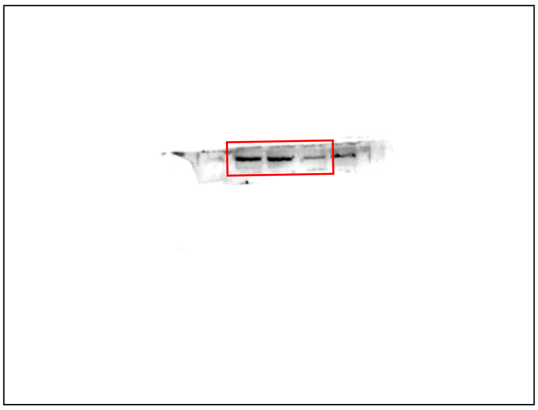

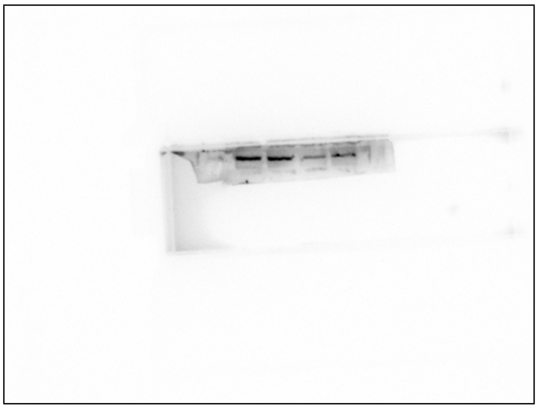

HNF1α

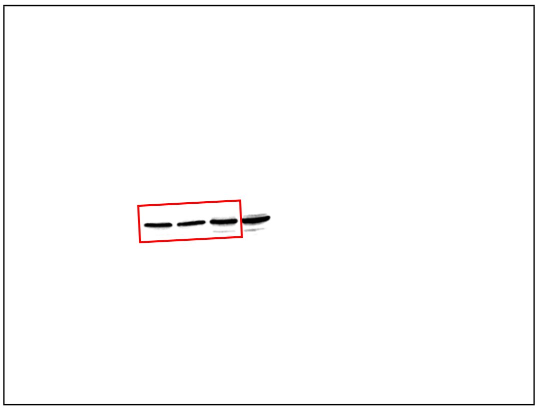

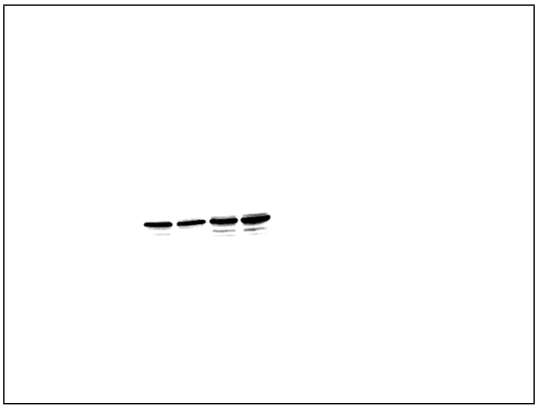

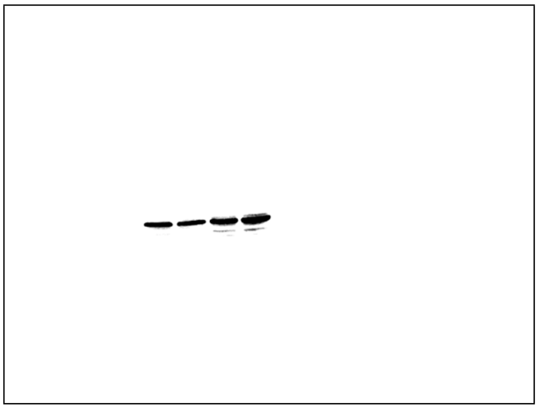

GAPDH

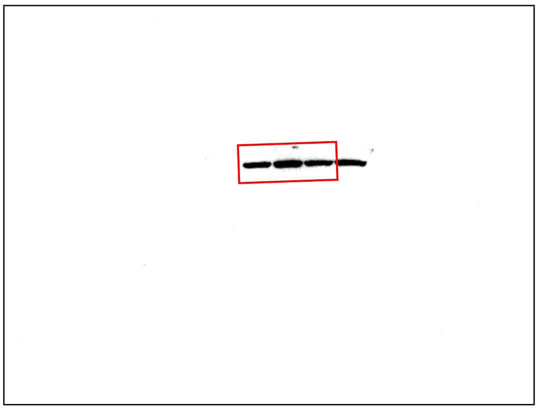

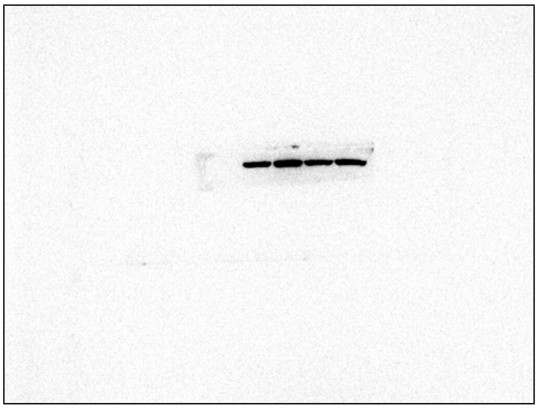

Figure 4B

RelA

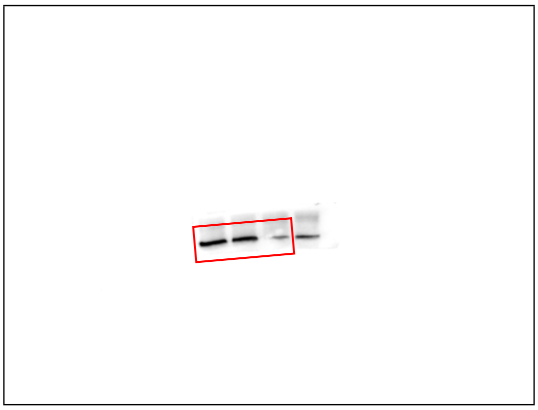

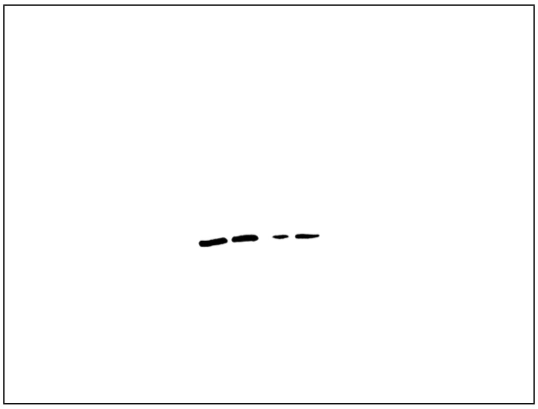

HNF1α

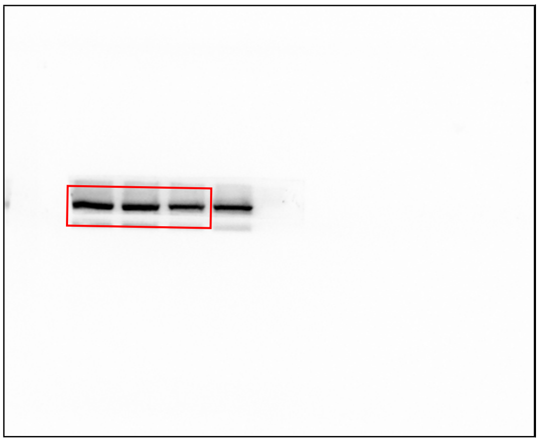

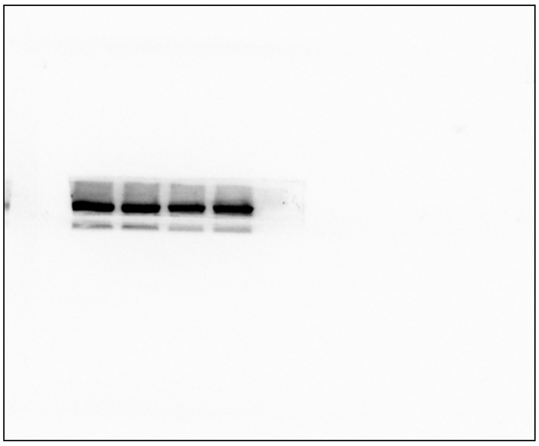

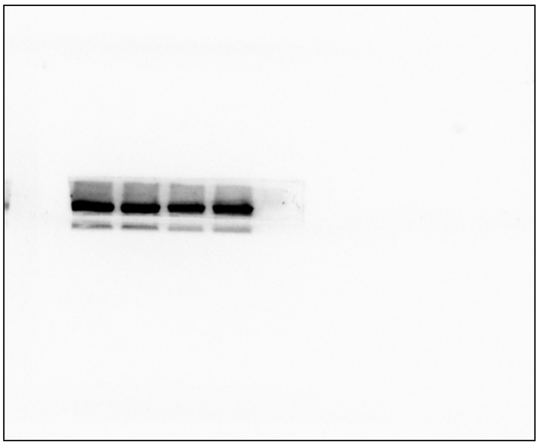

GAPDH

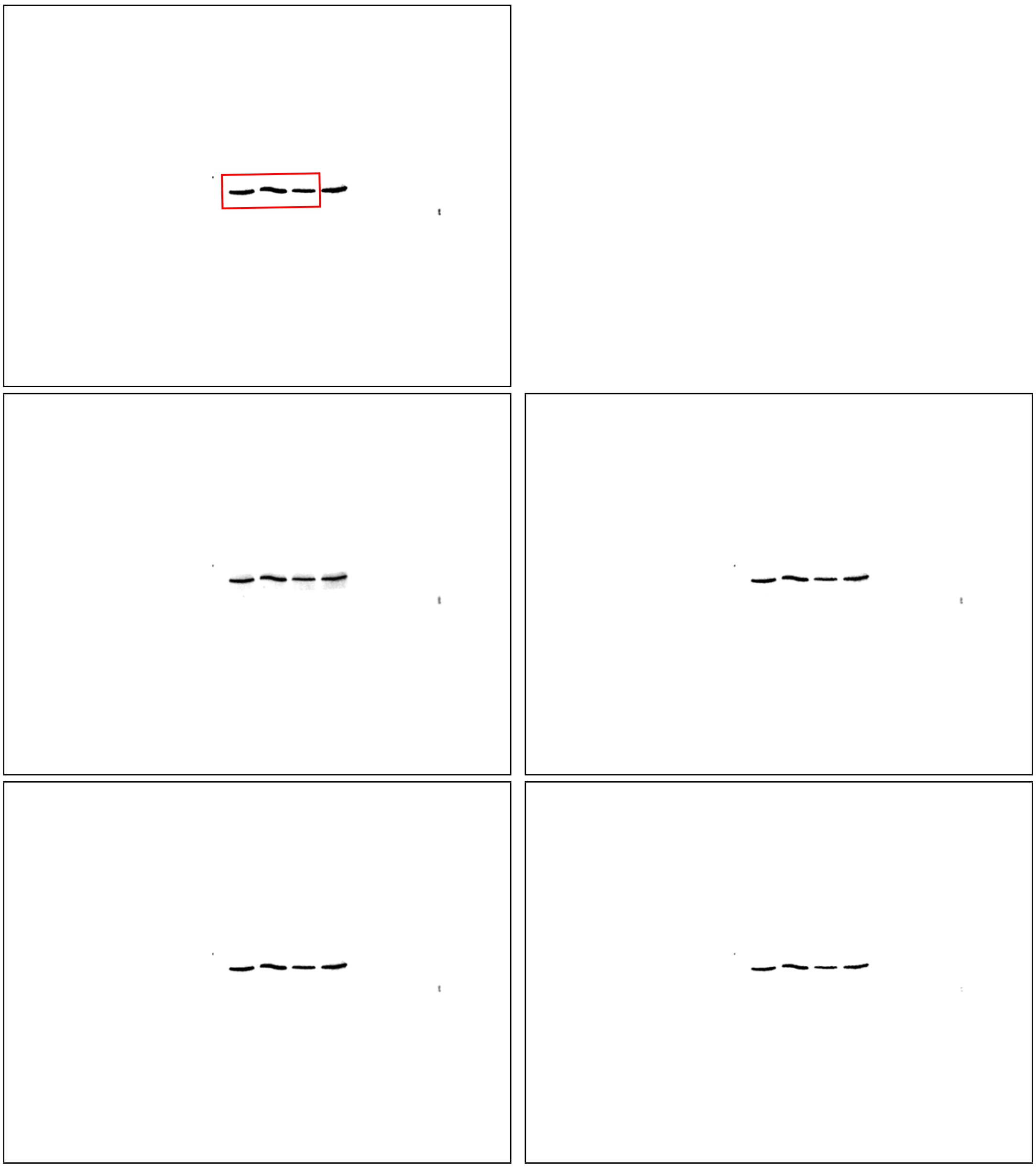

Figure 4D

RelA

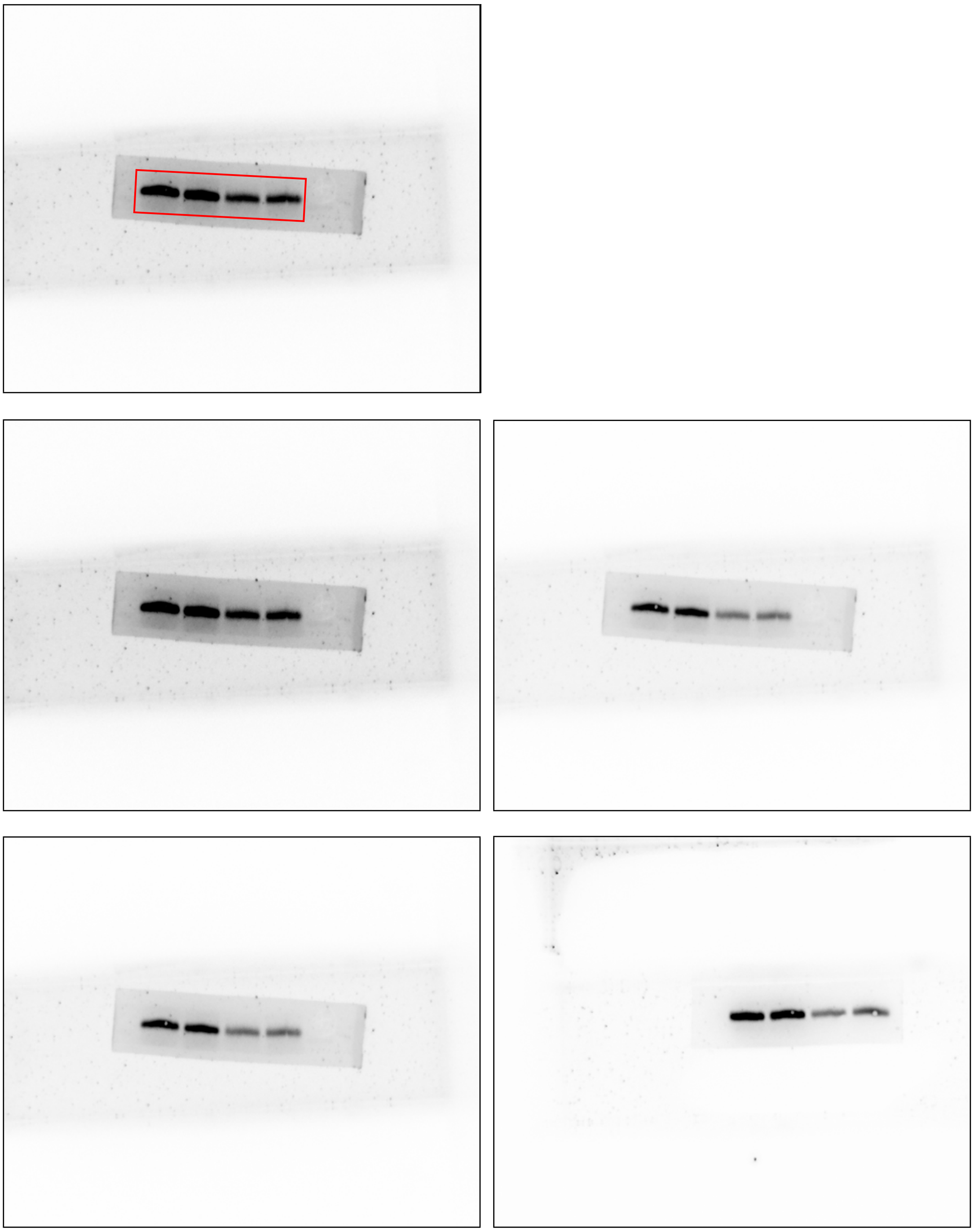

HNF1α

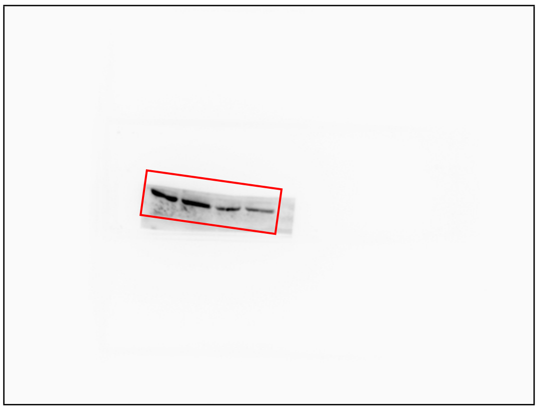

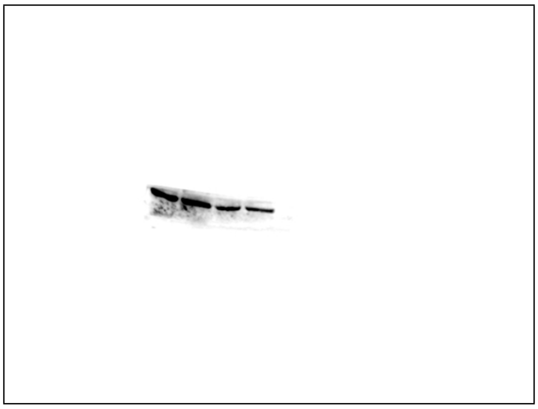

ATF4

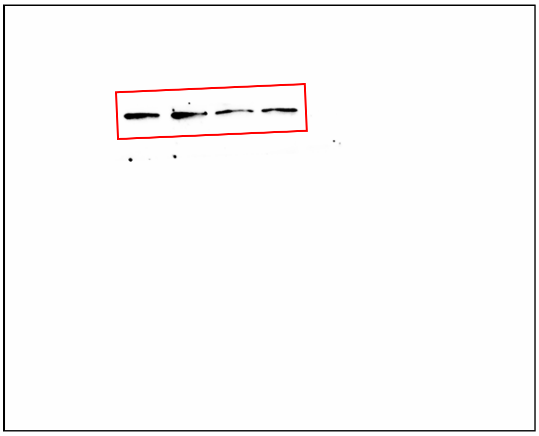

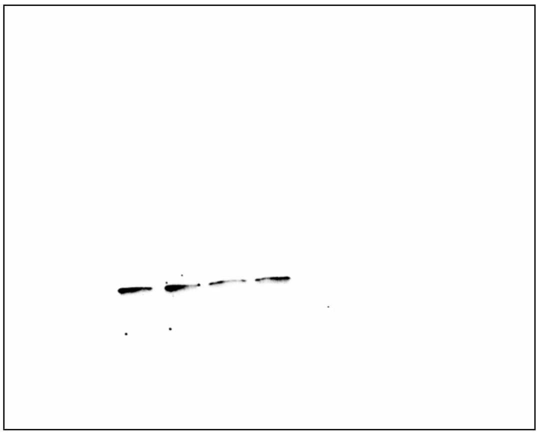

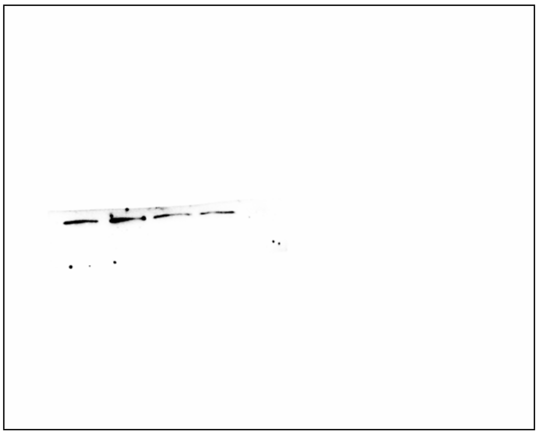

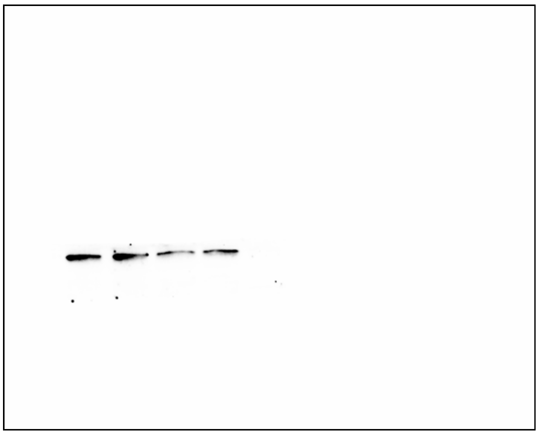

GRP78

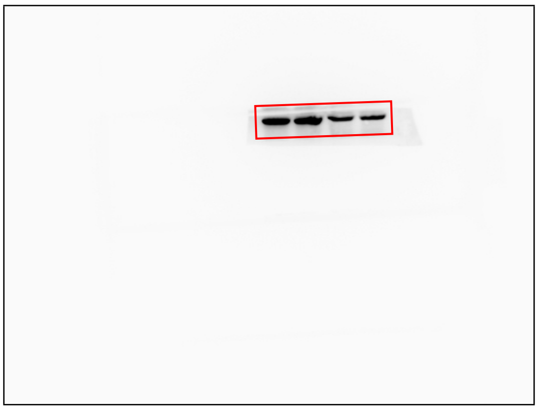

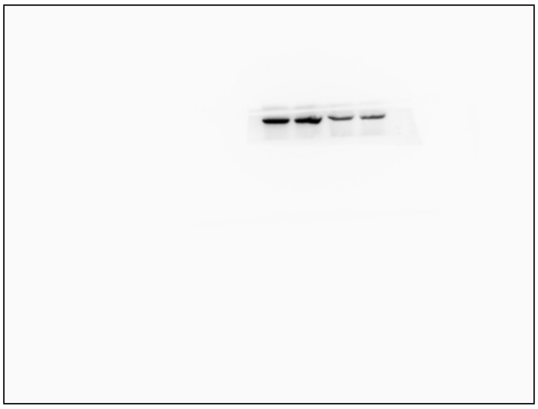

Cleaved caspase-3

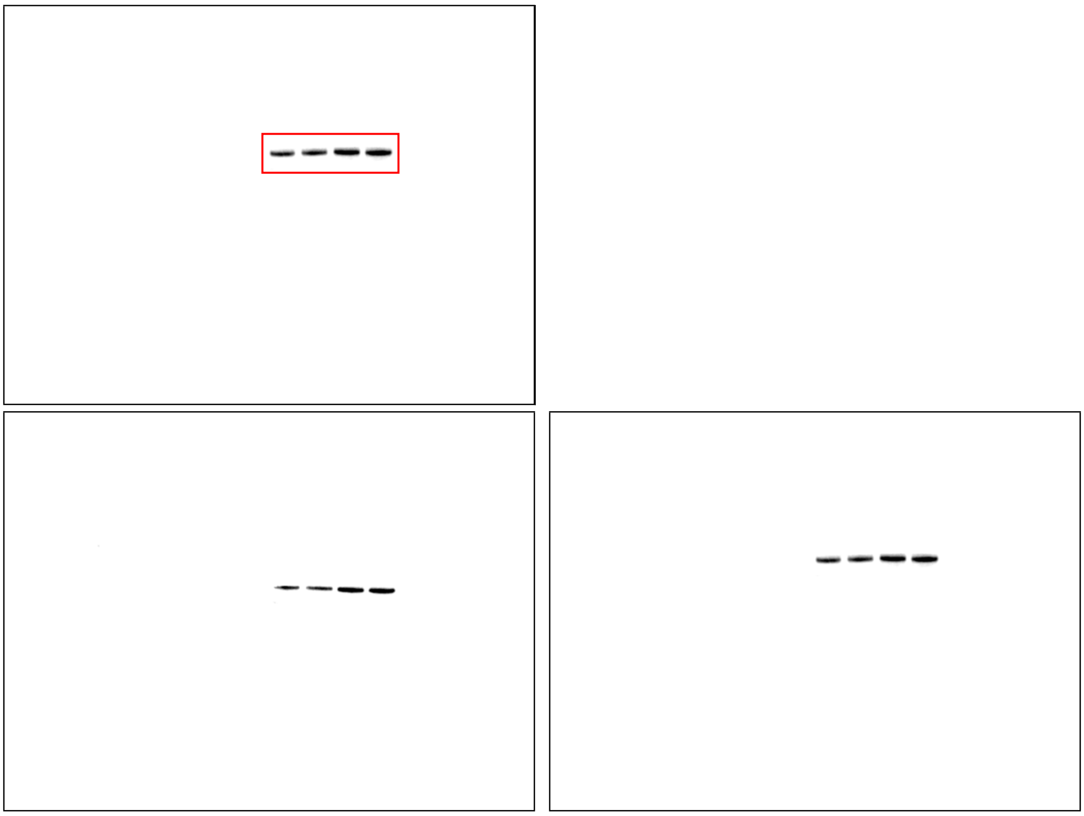

GAPDH

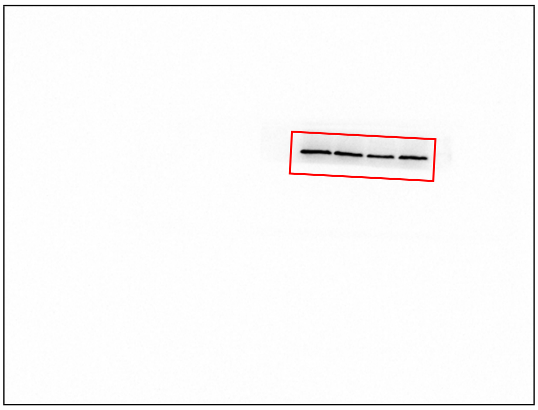

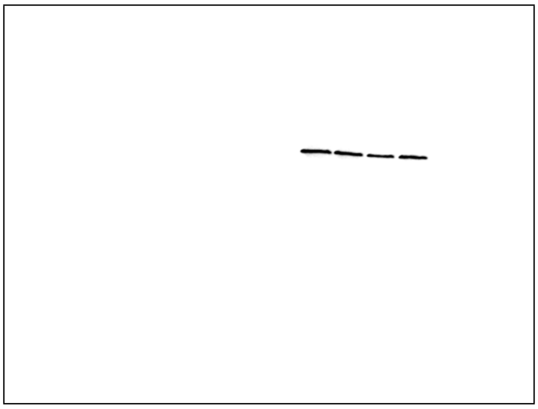

Figure 5D

HNF1α

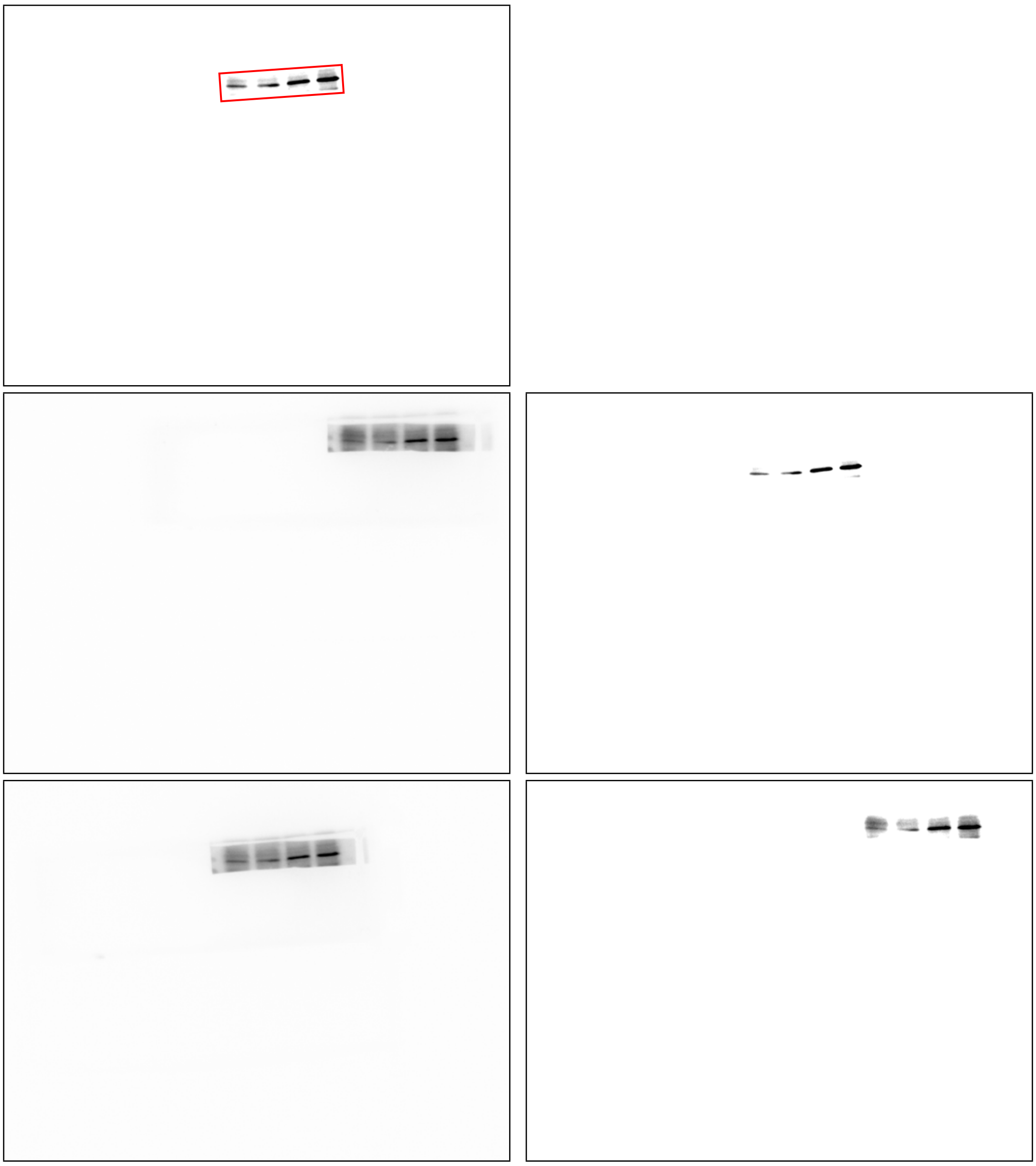

ATF4

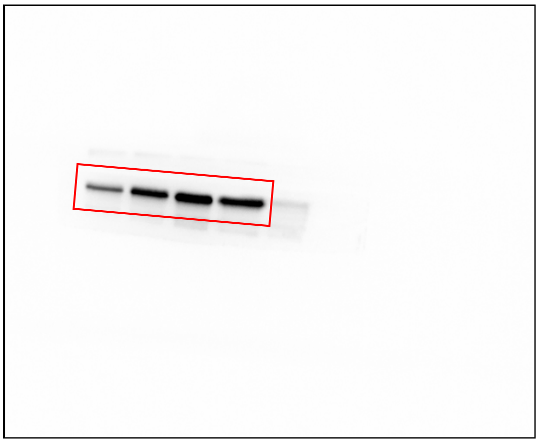

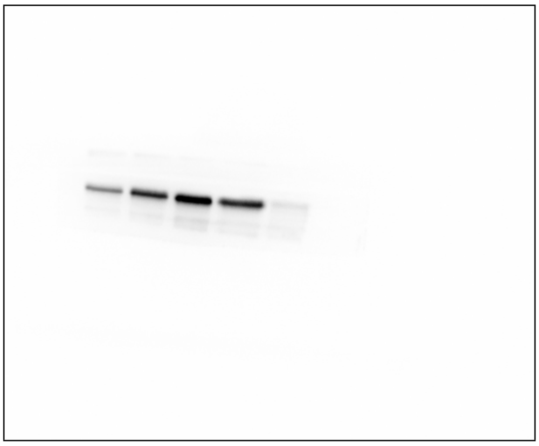

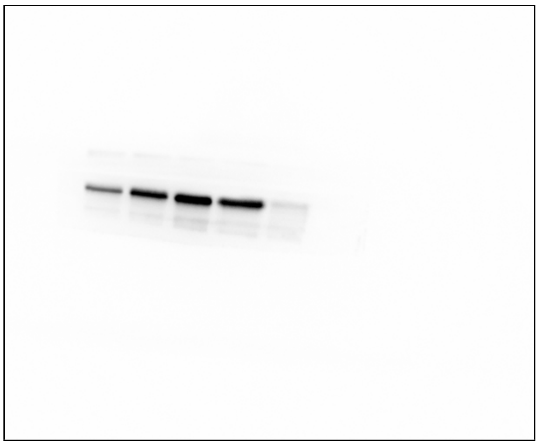

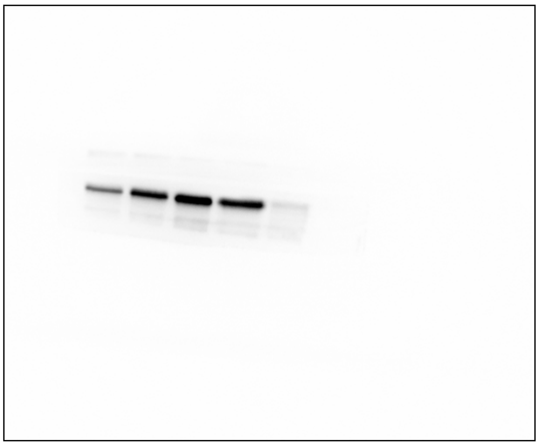

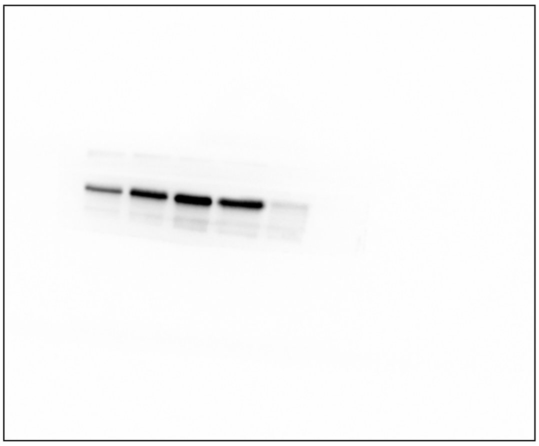

GRP78

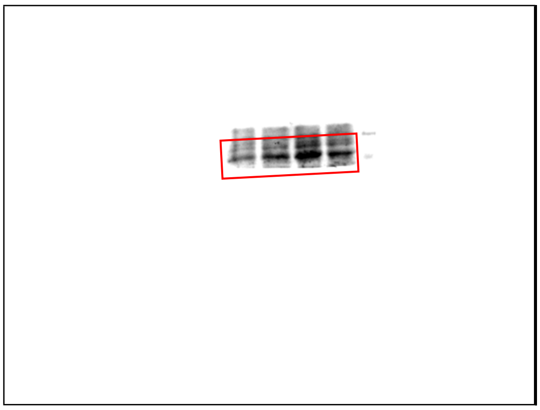

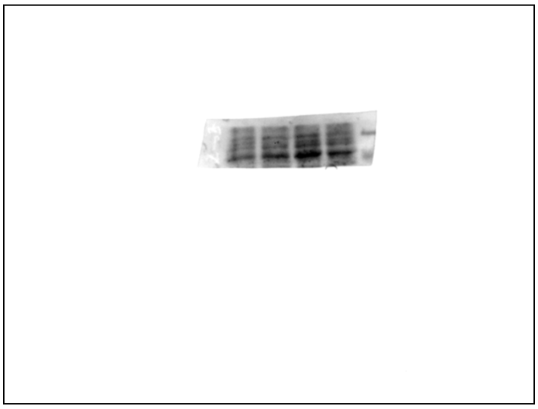

Caspase-12

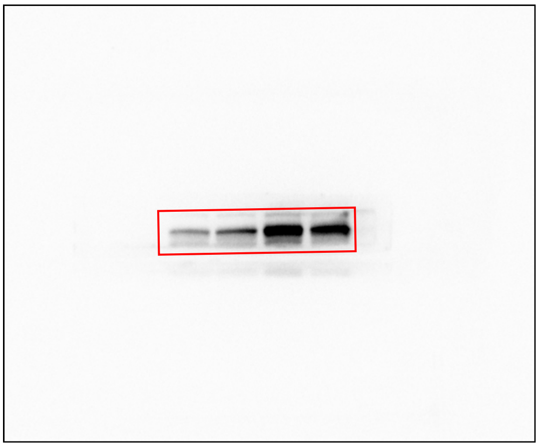

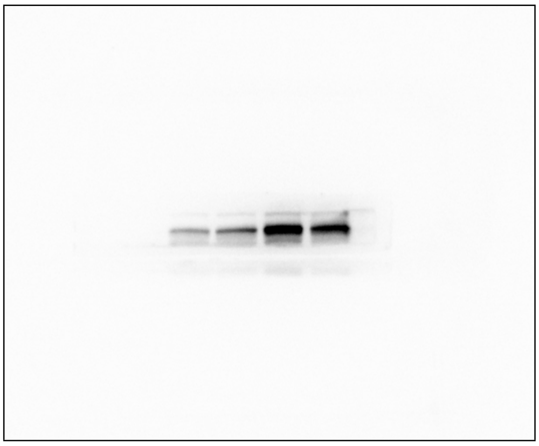

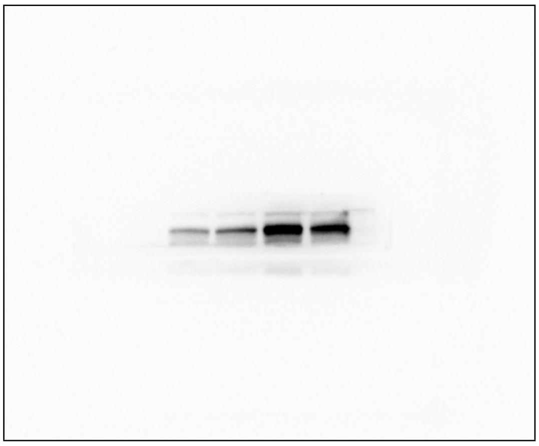

Cleaved caspase-3

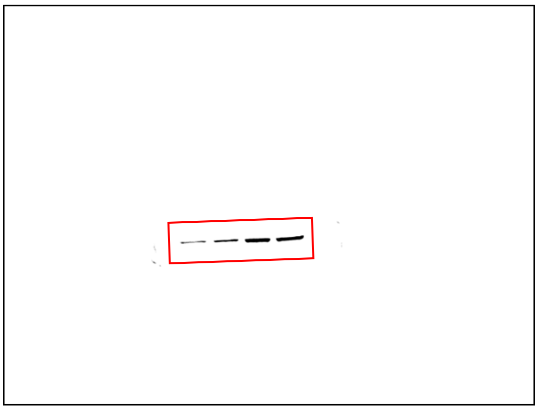

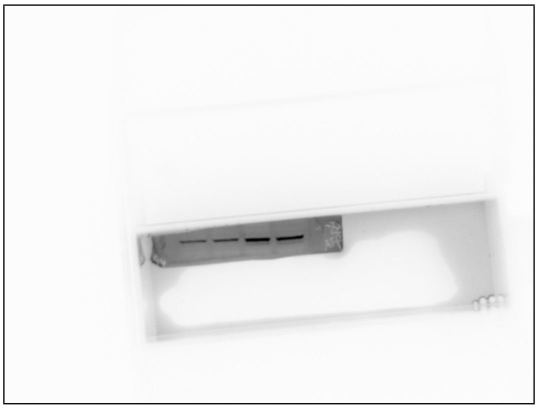

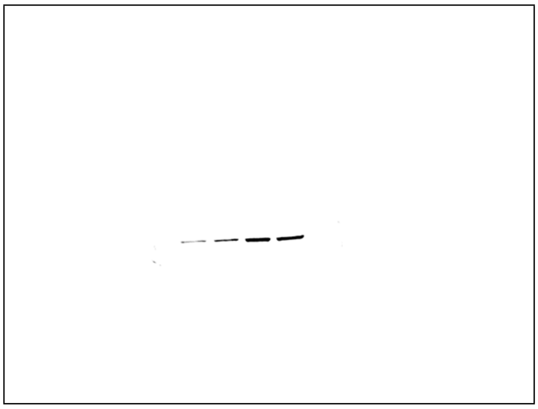

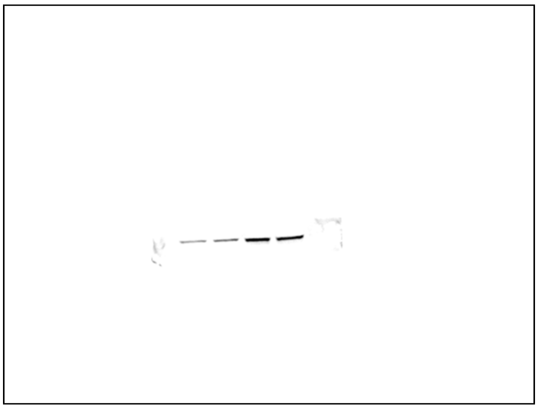

GAPDH

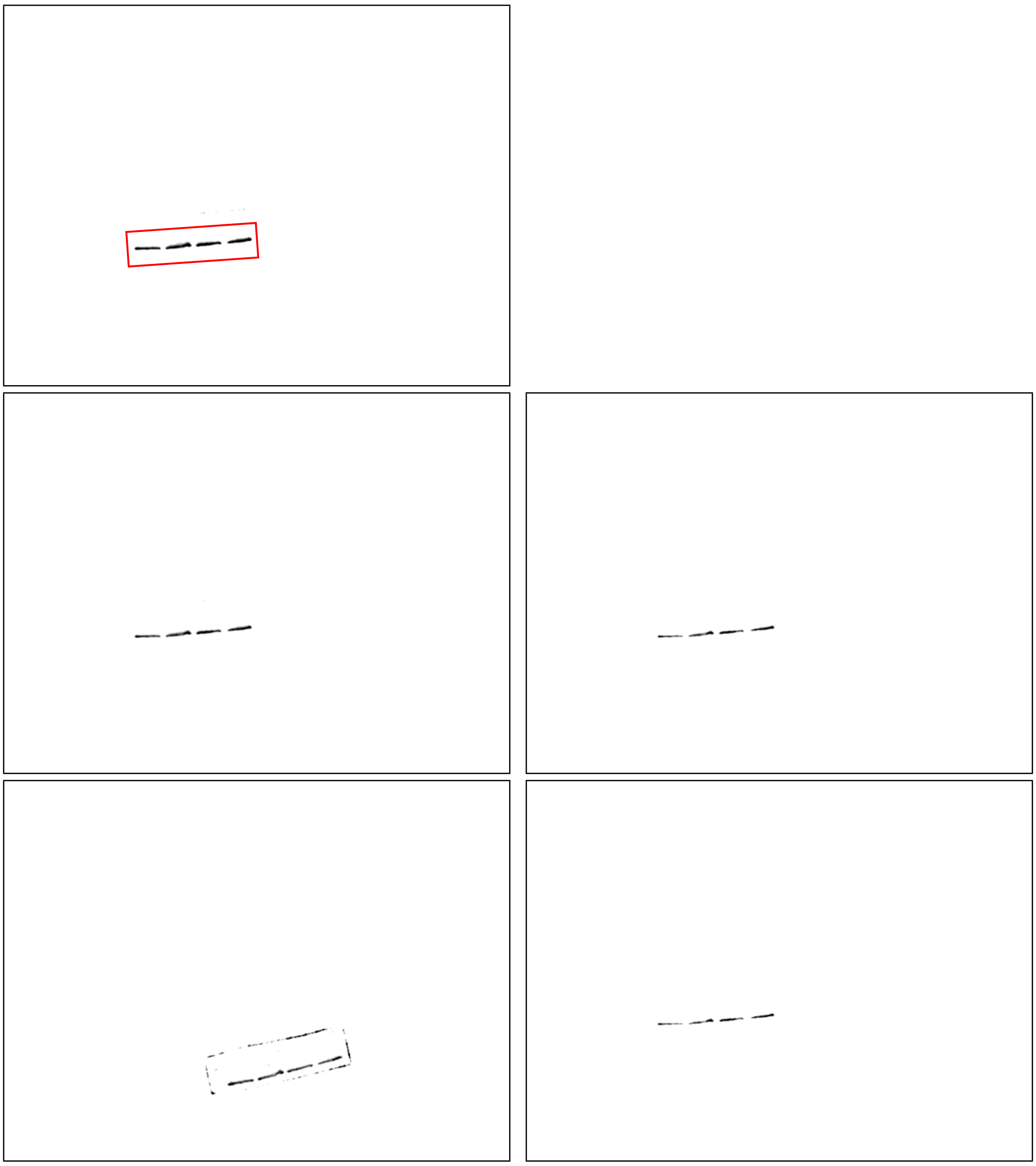

p-RelA

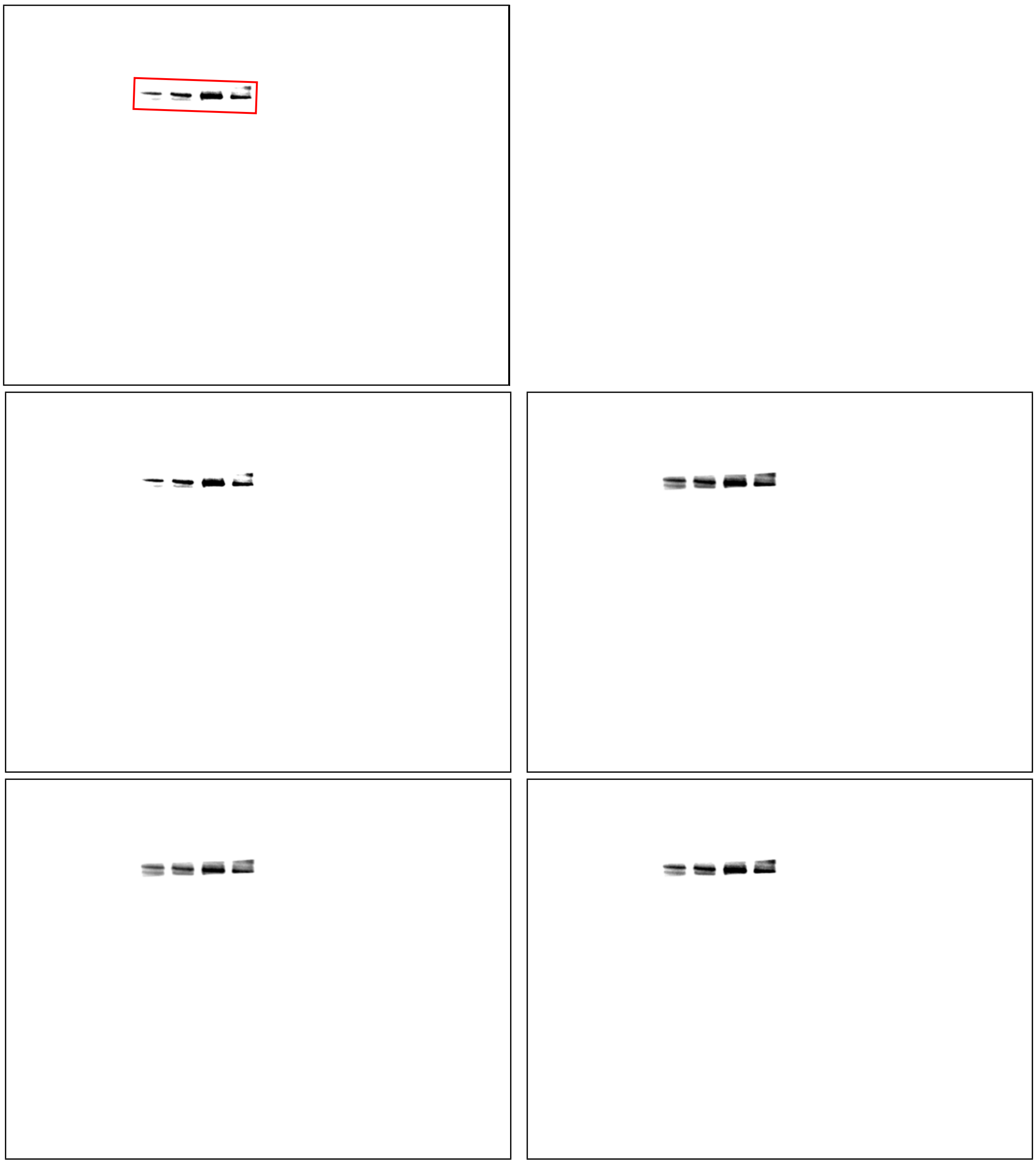

RelA

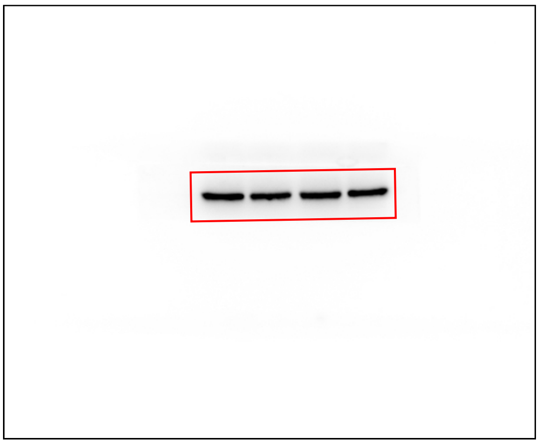

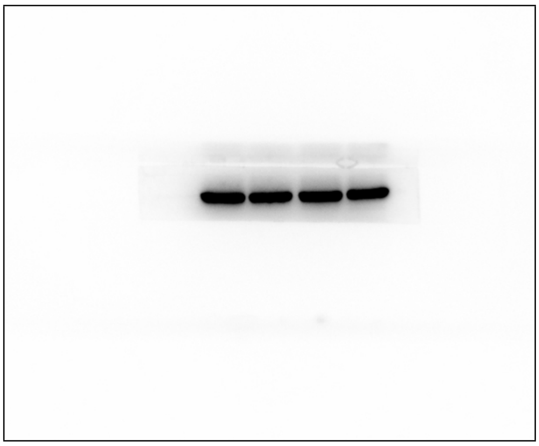

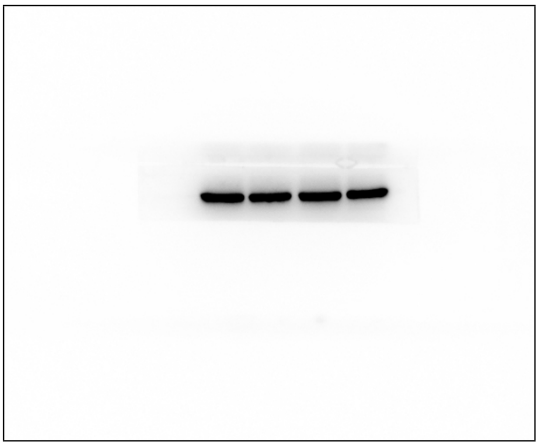

Figure 5I

HNF1α

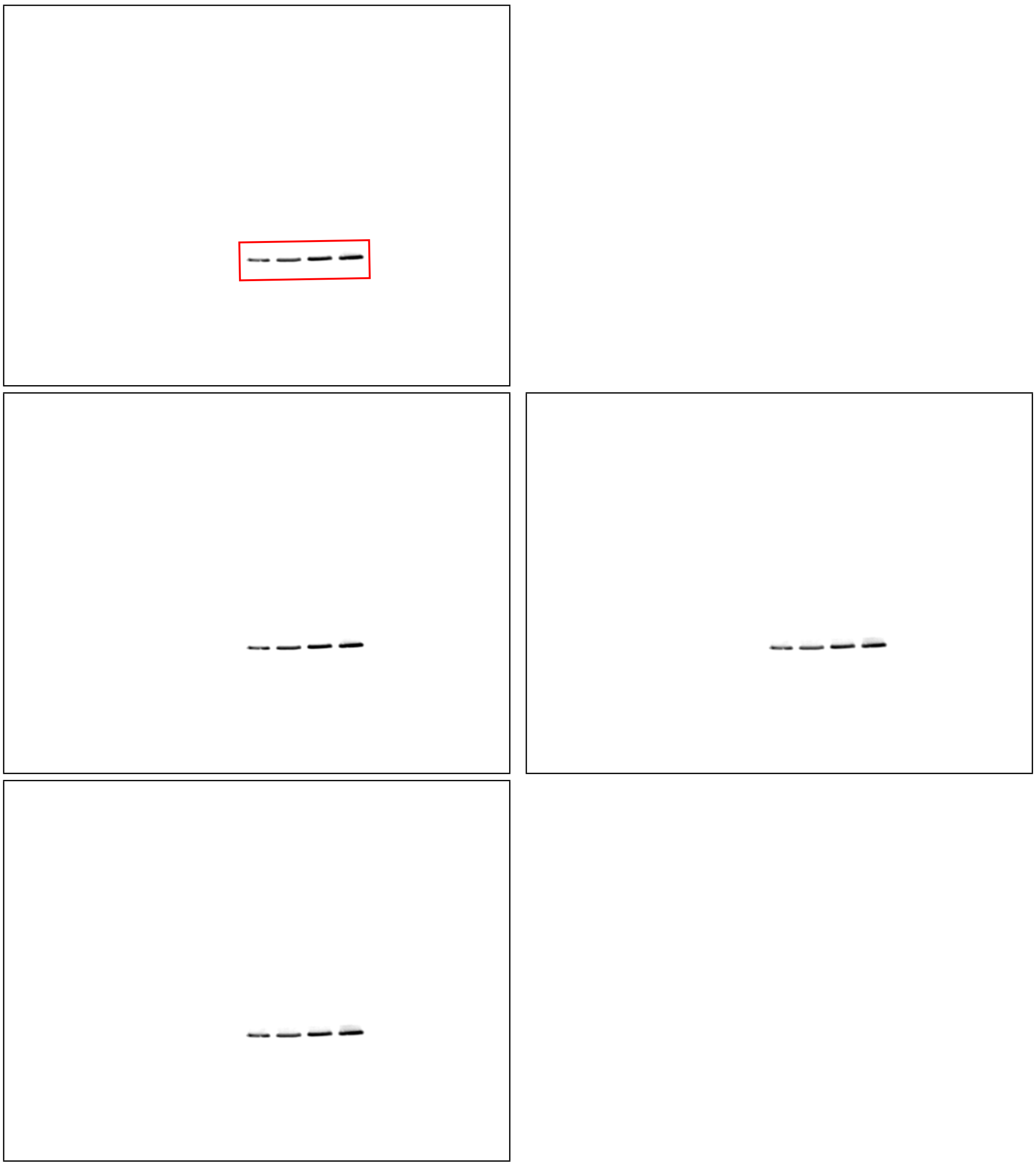

ATF4

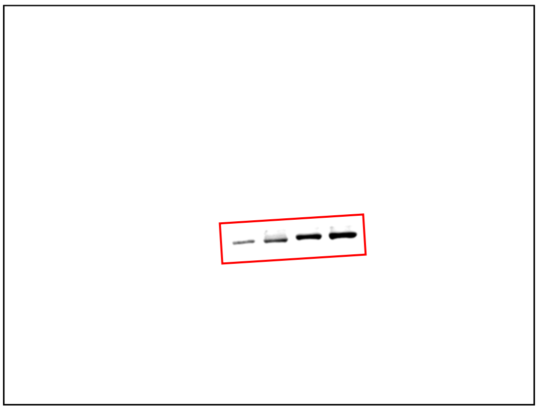

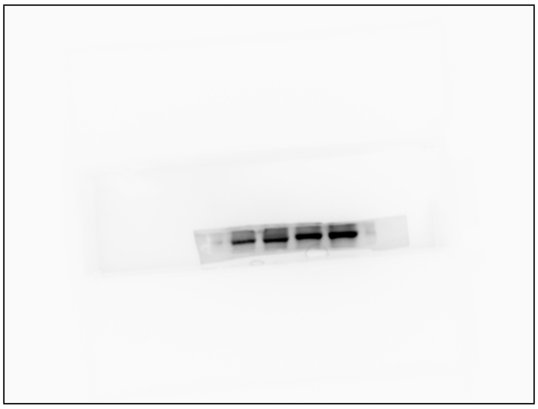

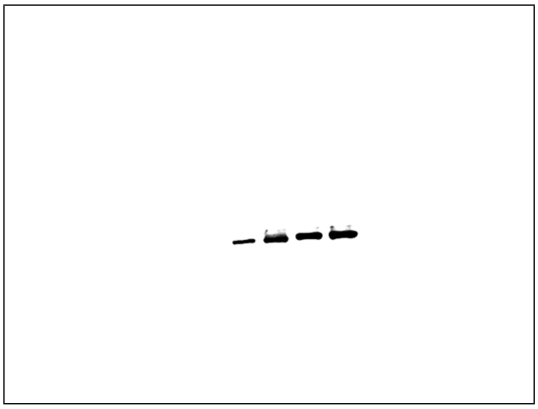

GRP78

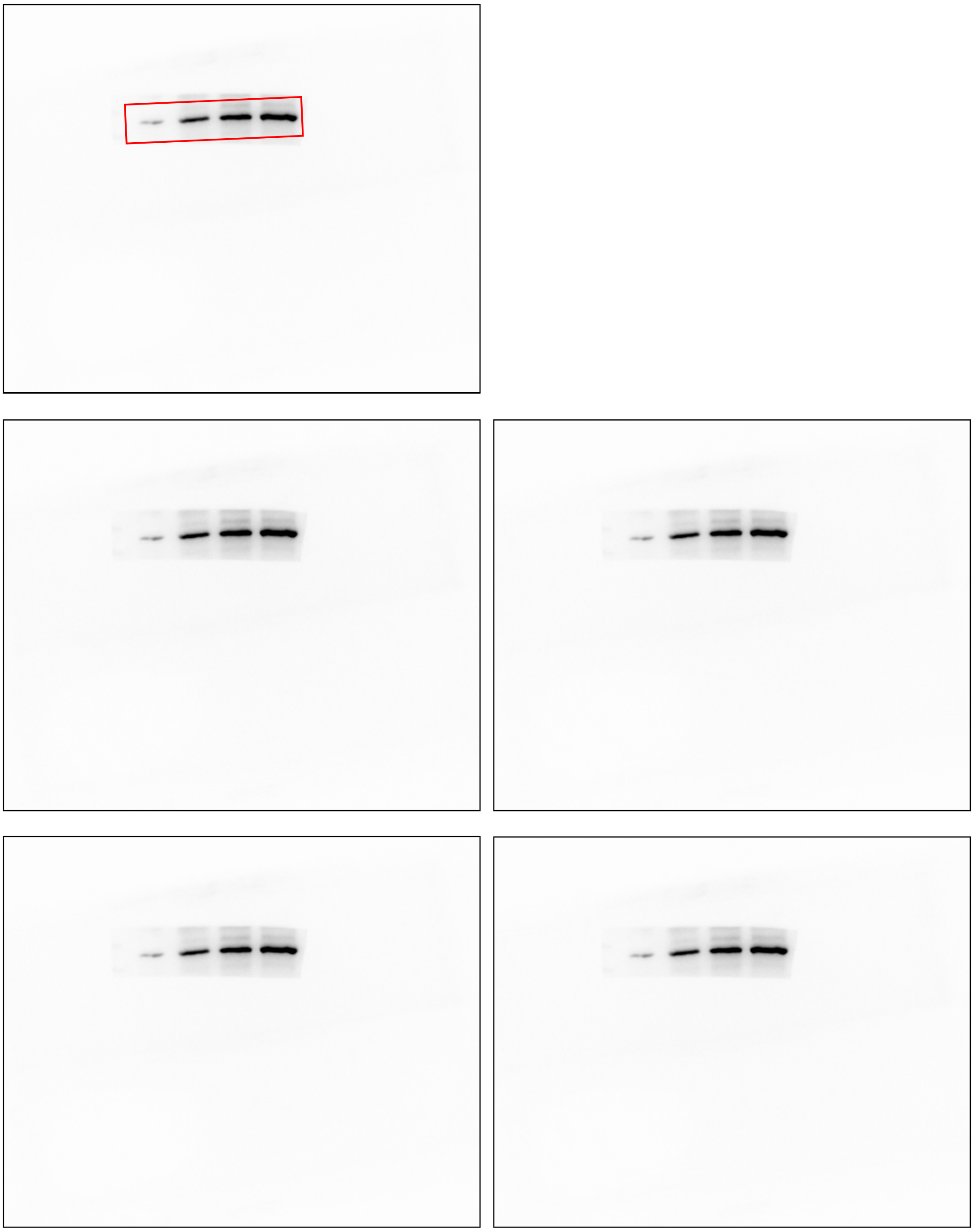

Caspase-12

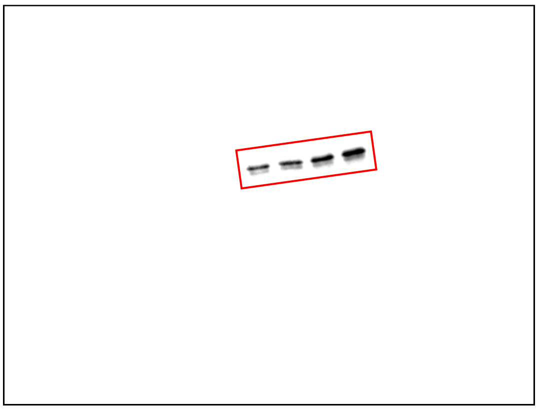

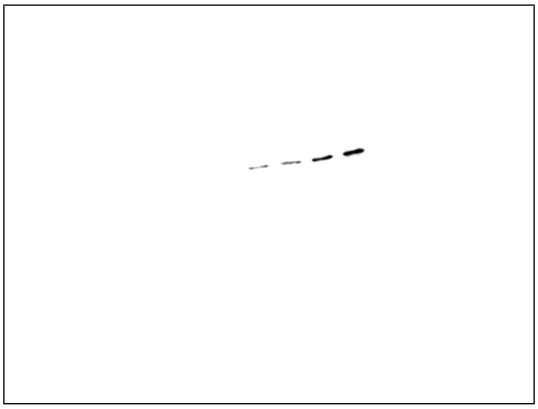

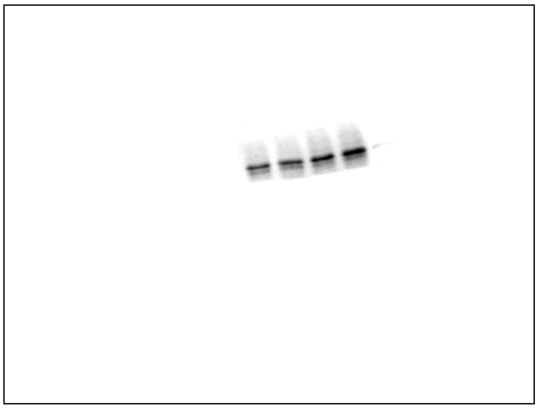

Cleaved caspase-3

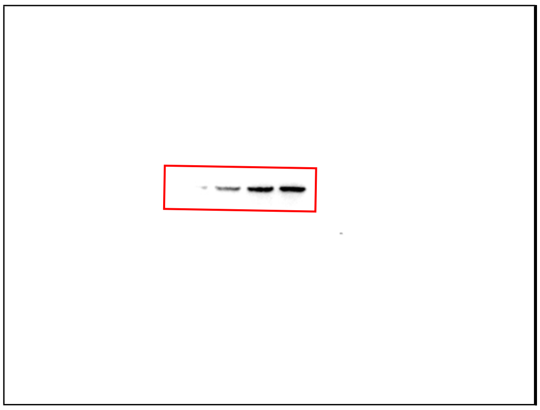

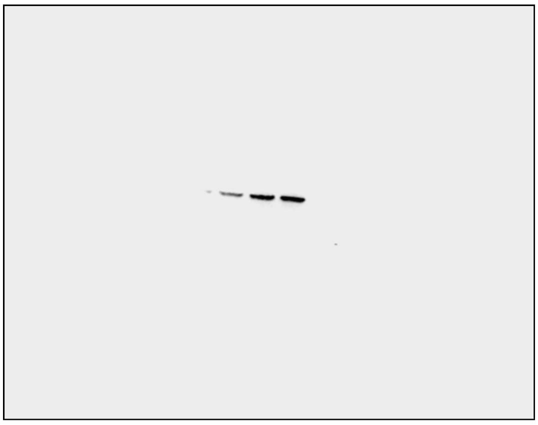

GAPDH

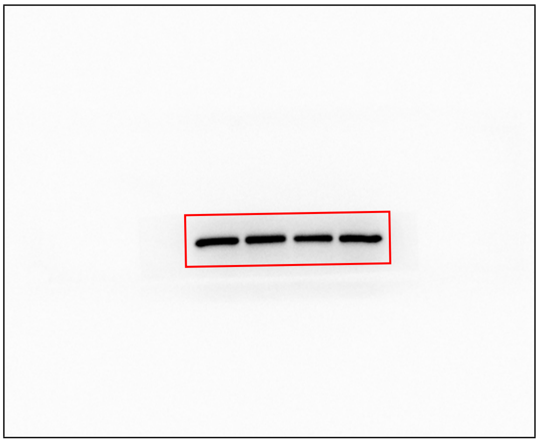

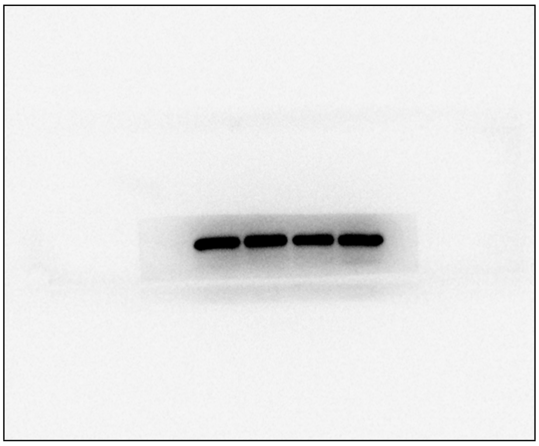

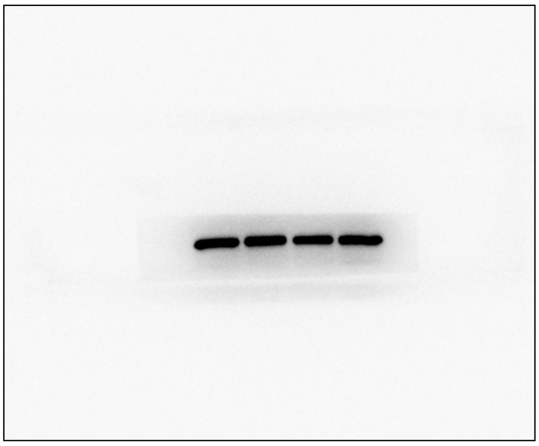

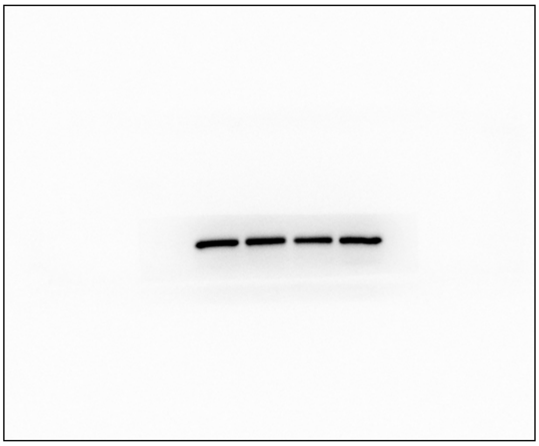

p-RelA

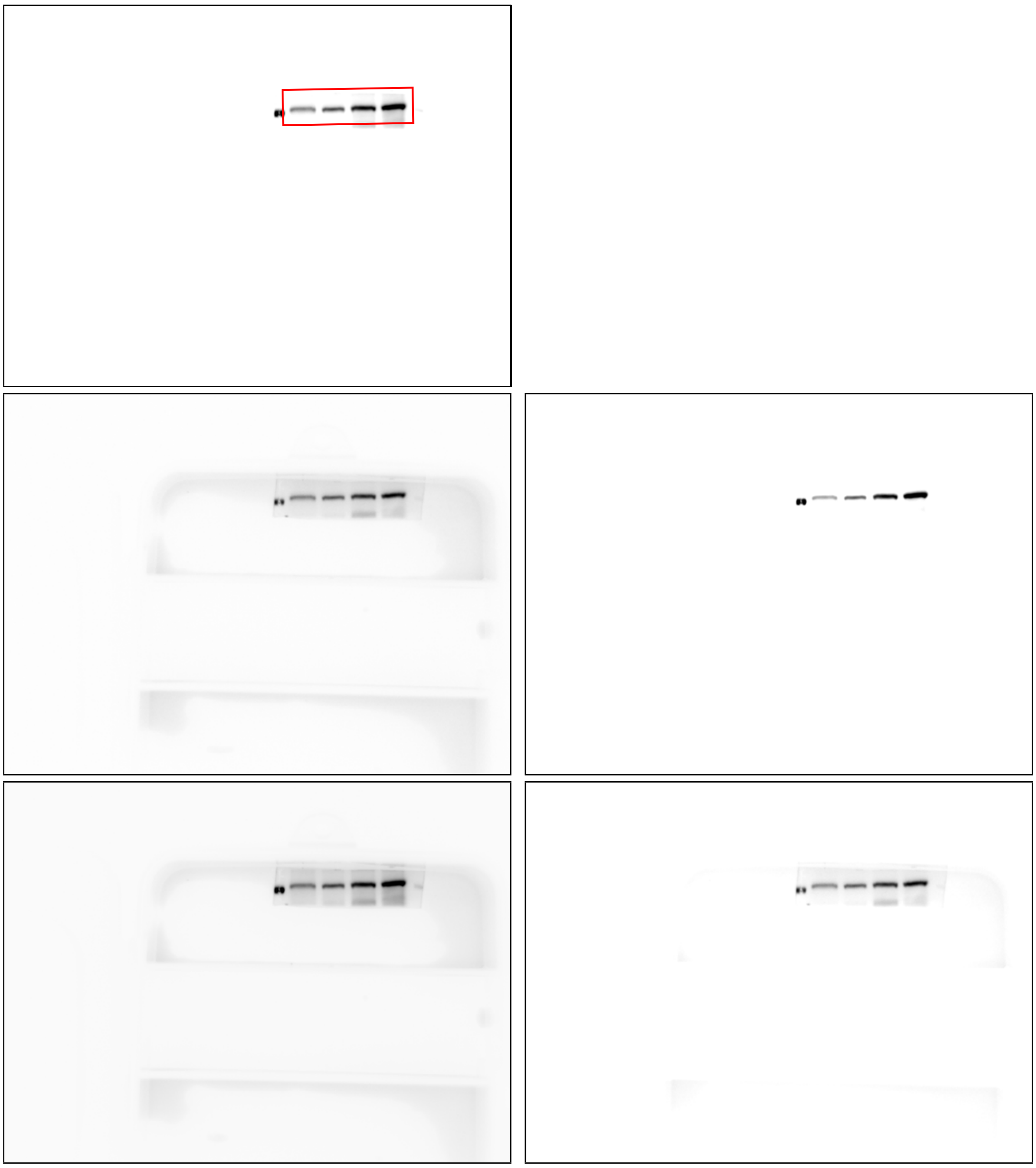

RelA

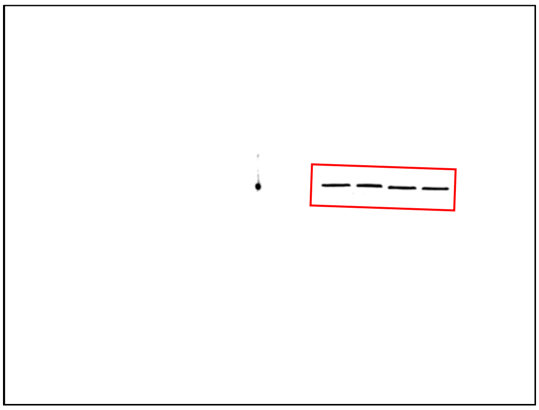

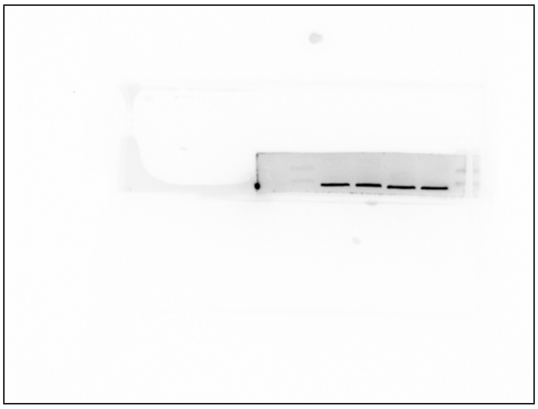

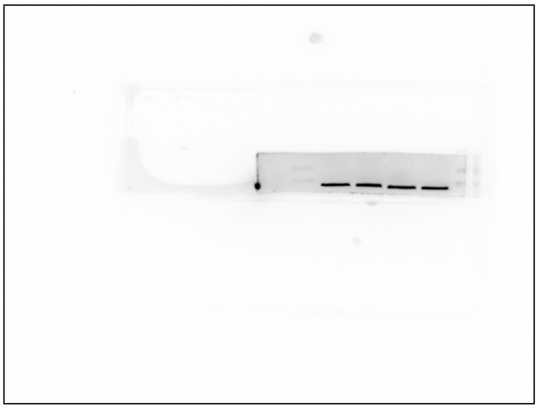

Figure 6A

HNF1α

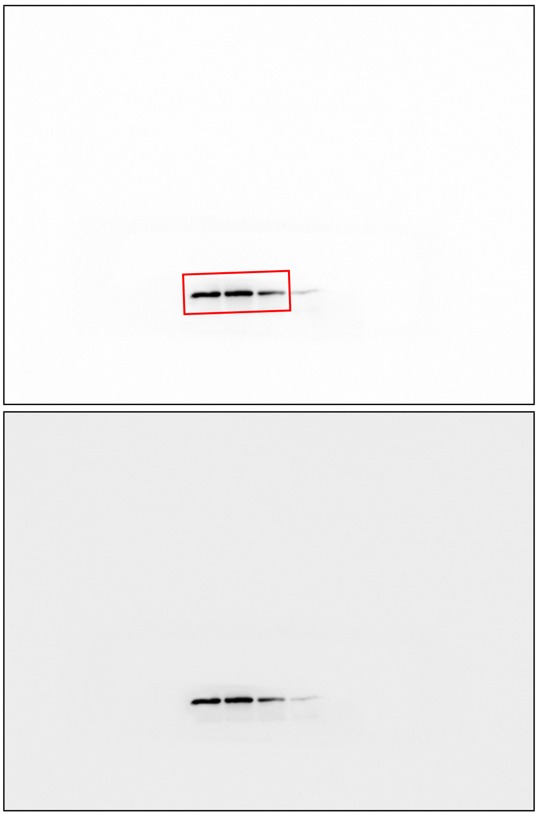

GAPDH

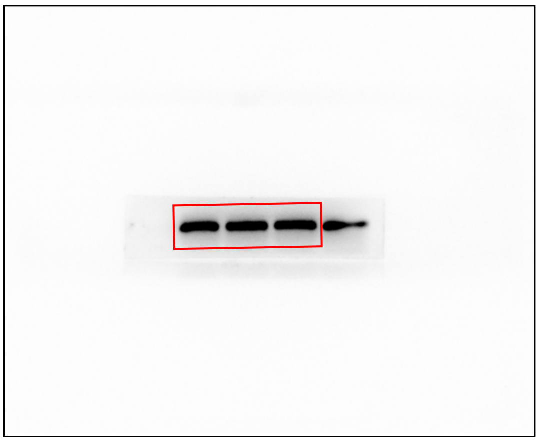

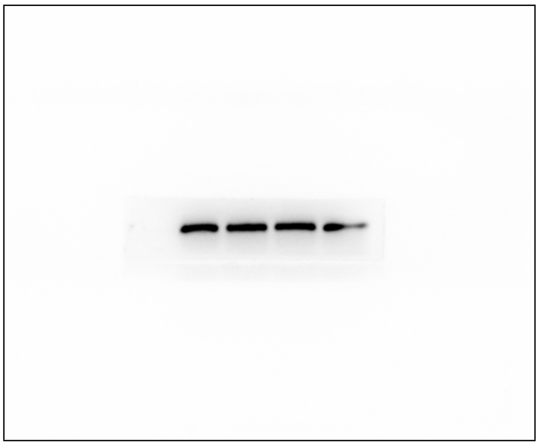

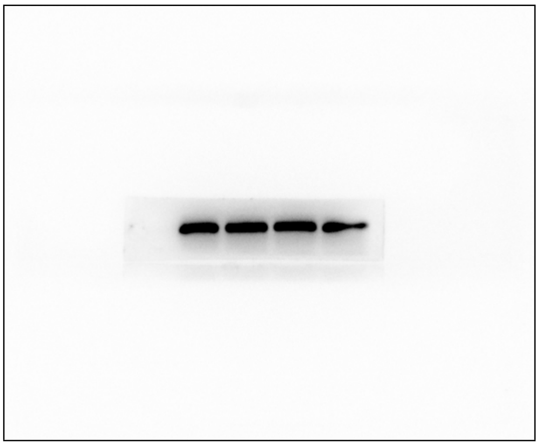

Fiure 6E

HNF1α

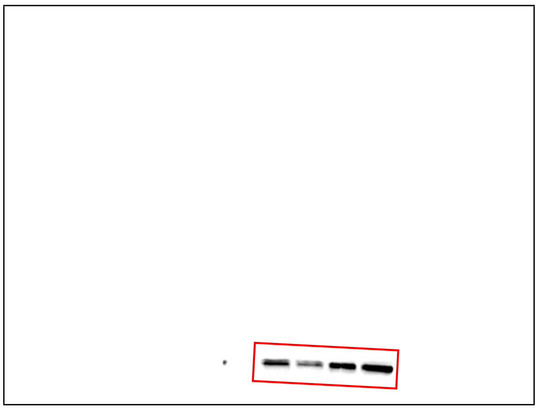

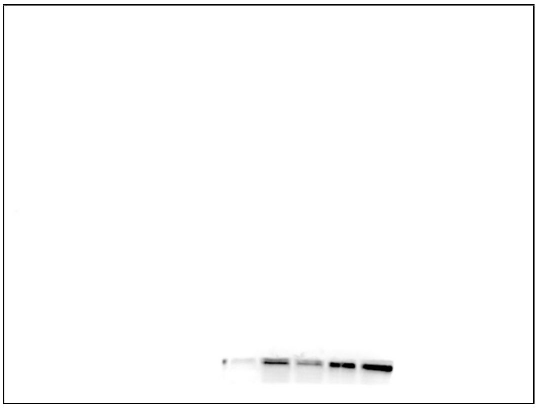

ATF4

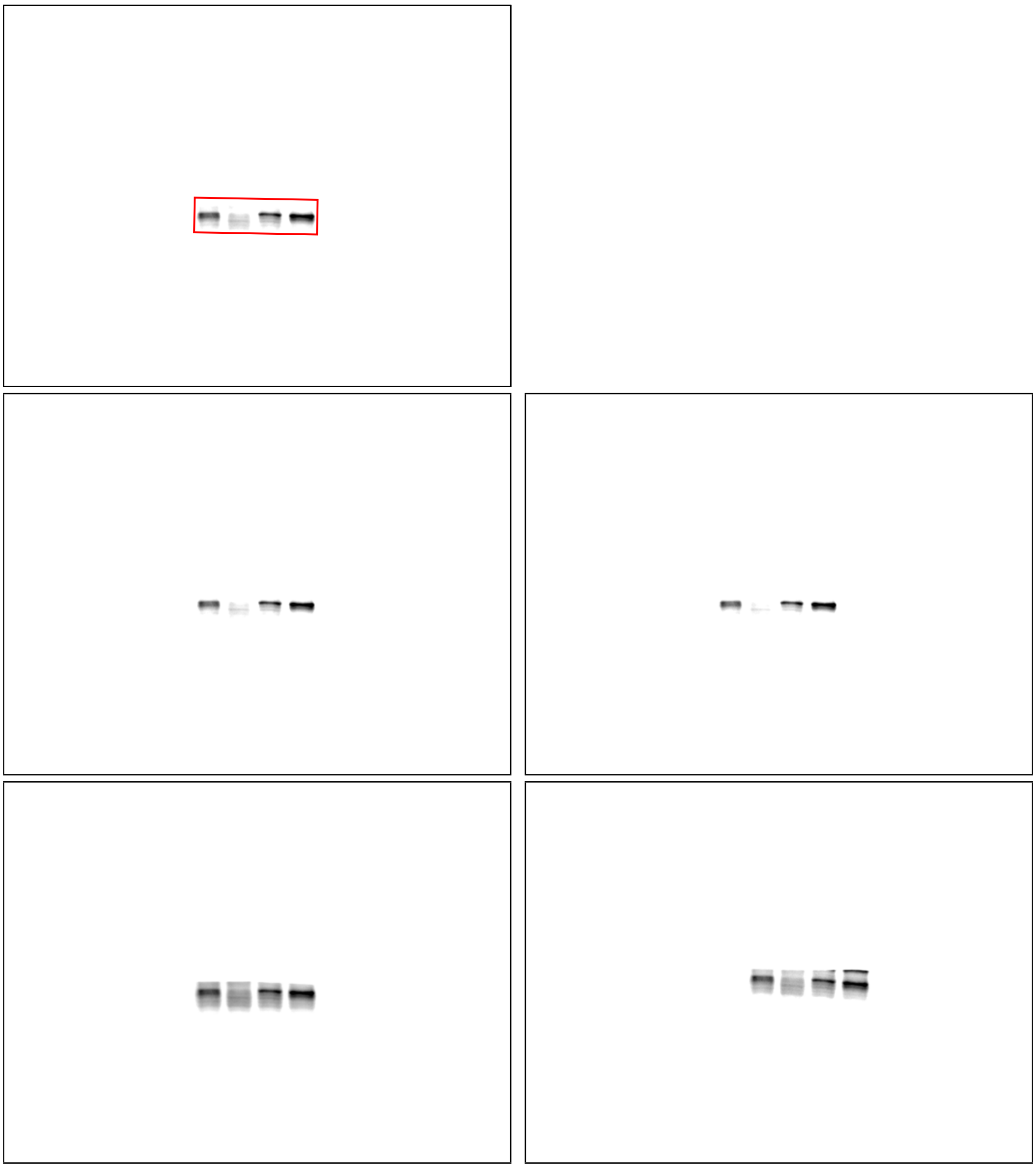

GRP78

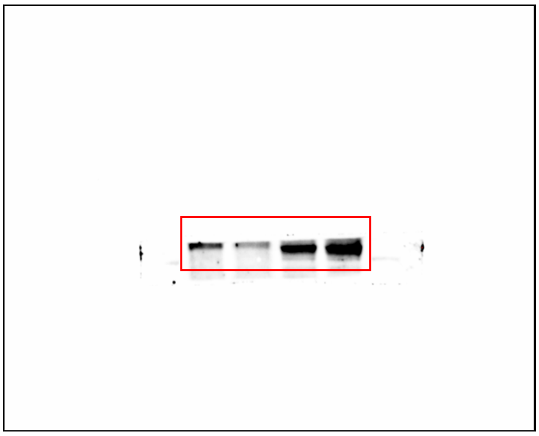

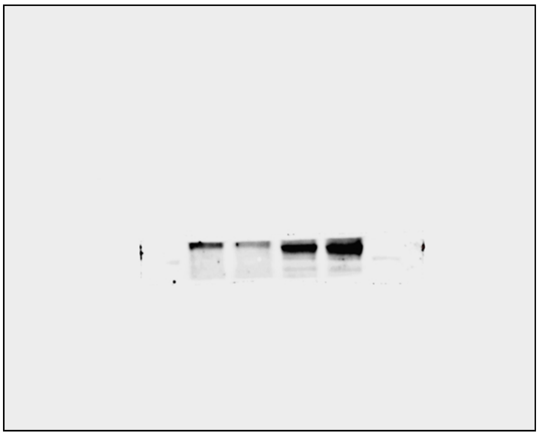

Caspase-12

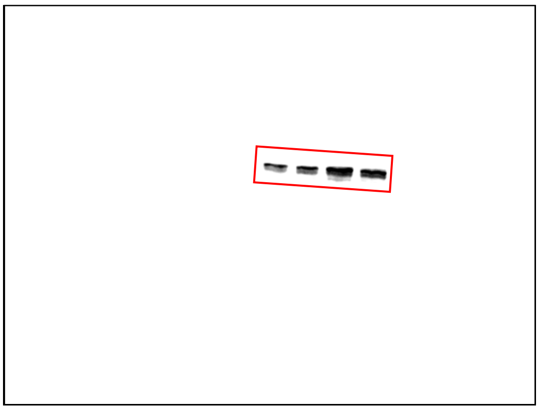

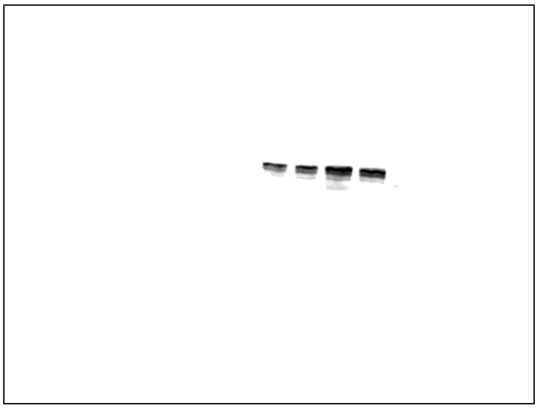

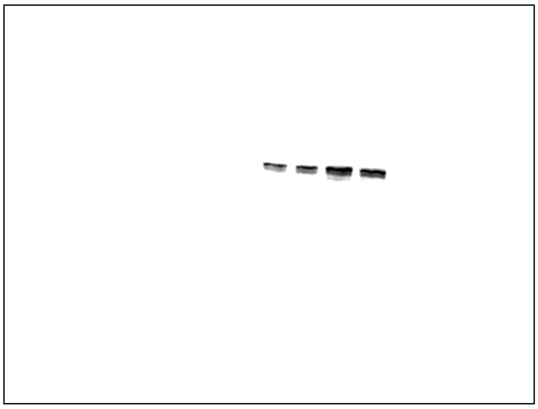

Cleaved caspase-3

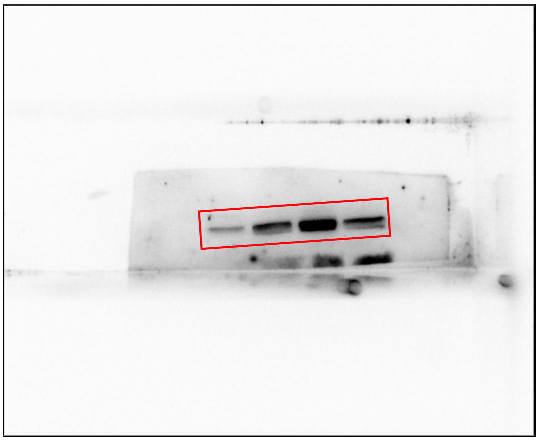

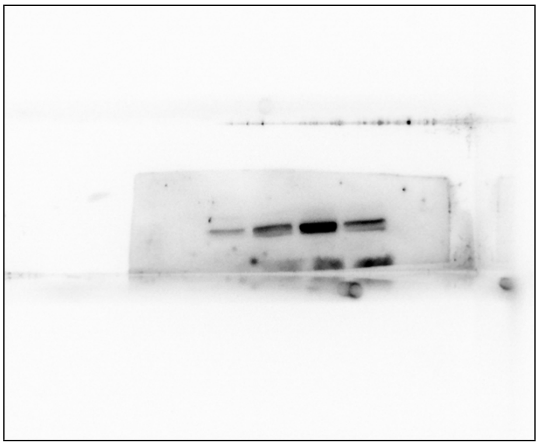

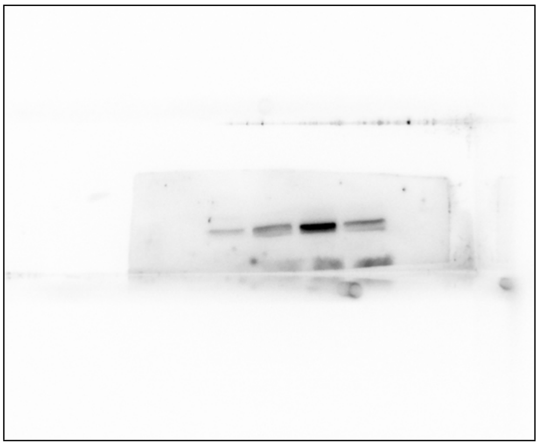

GAPDH

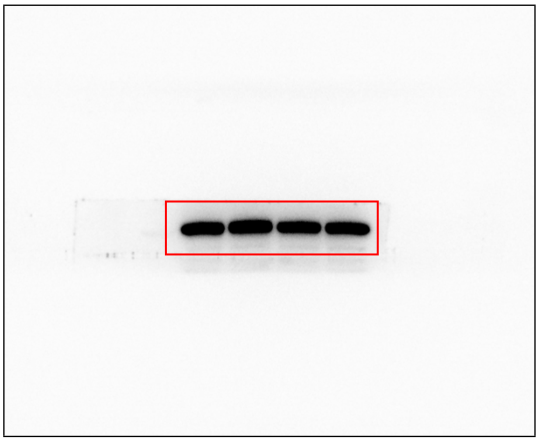

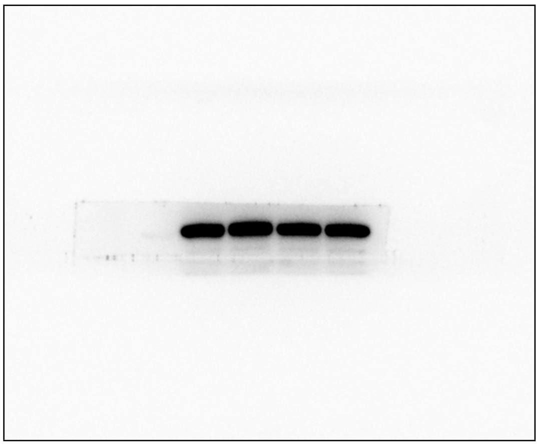

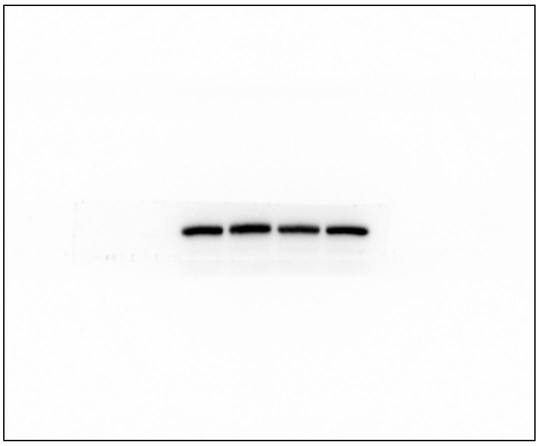

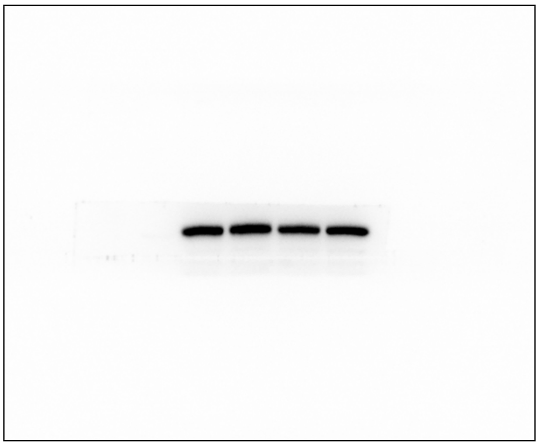

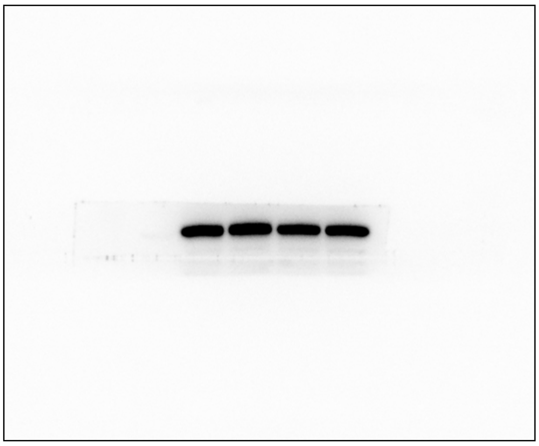

p-RelA

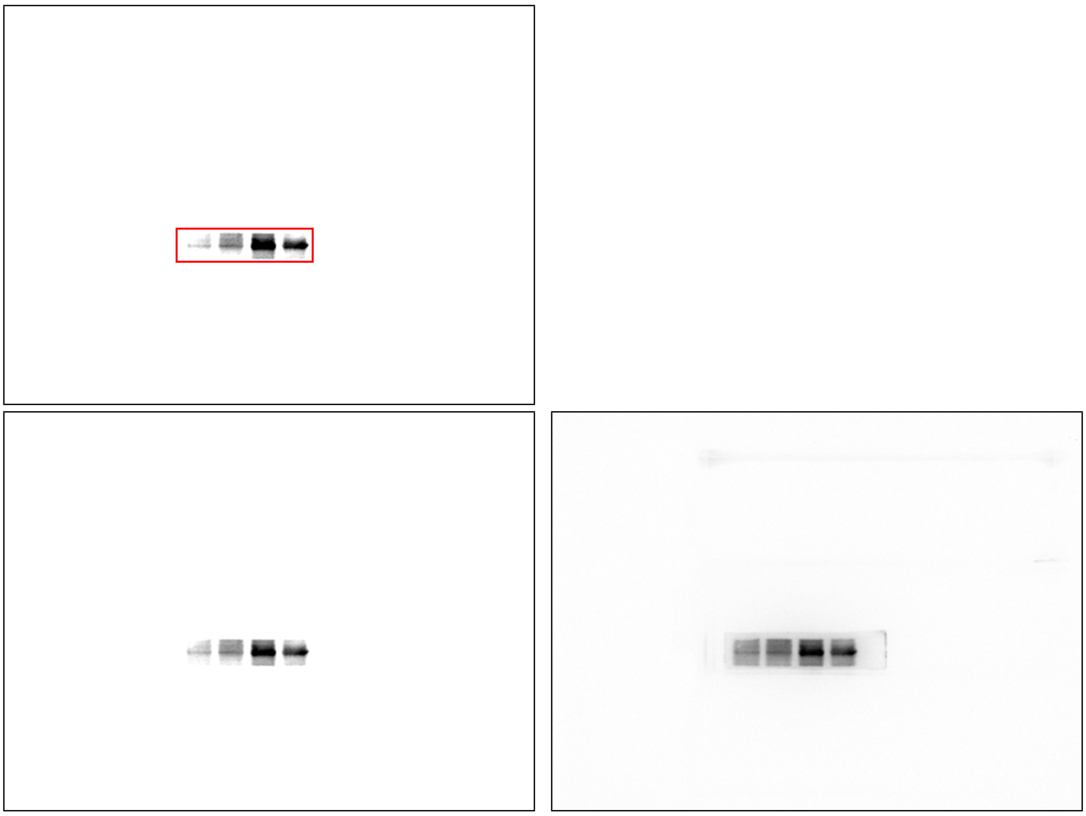

RelA

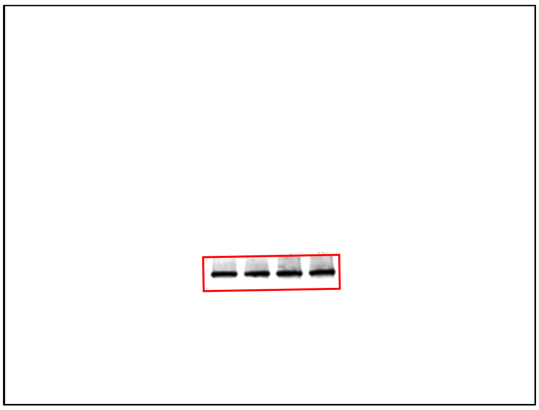

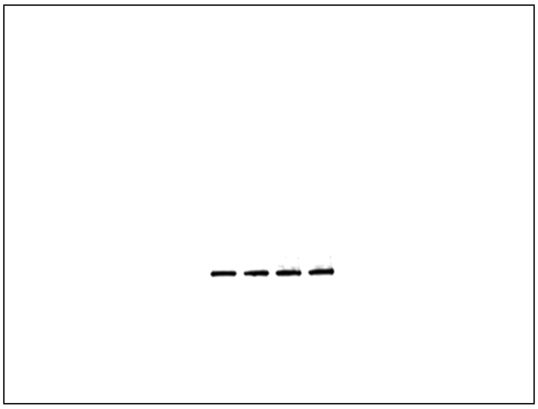

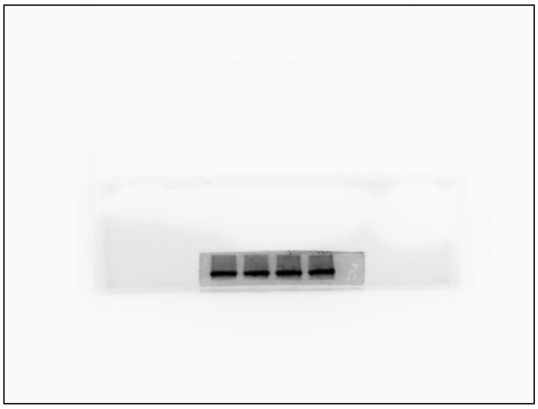

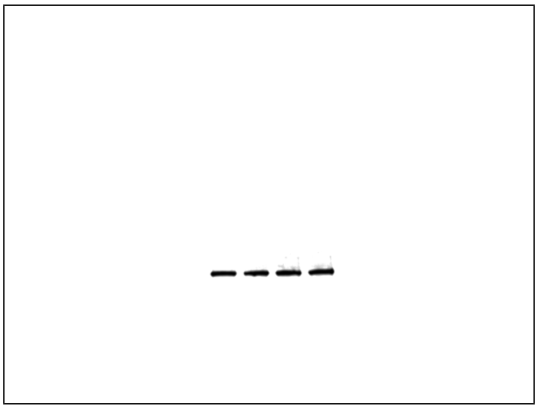

Figure 7D

HNF1α

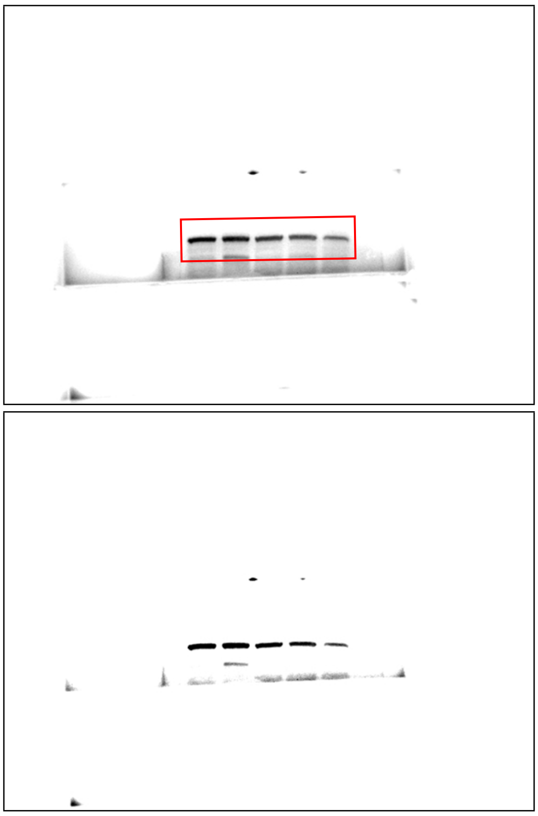

ATF4

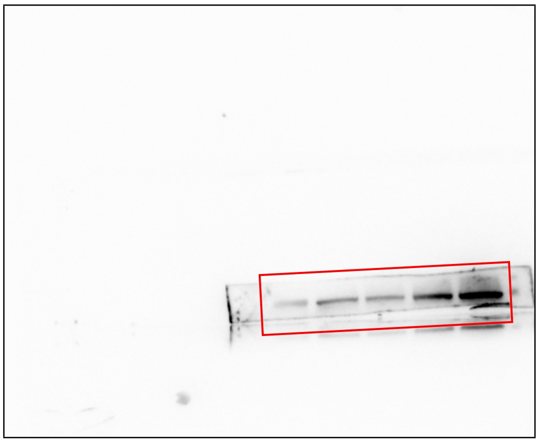

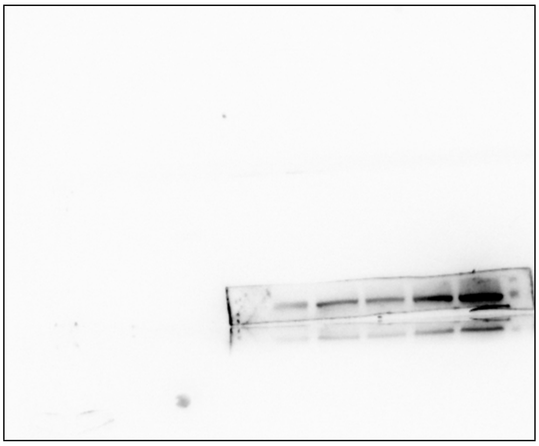

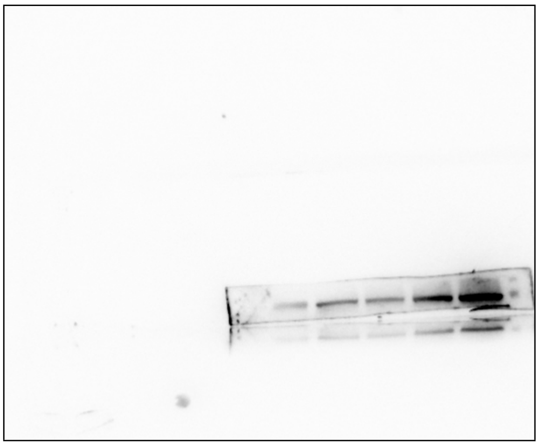

GRP78

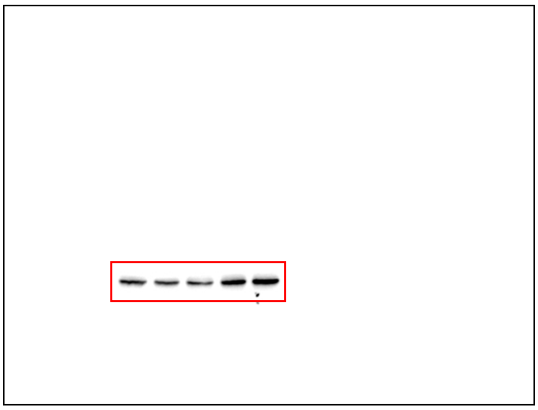

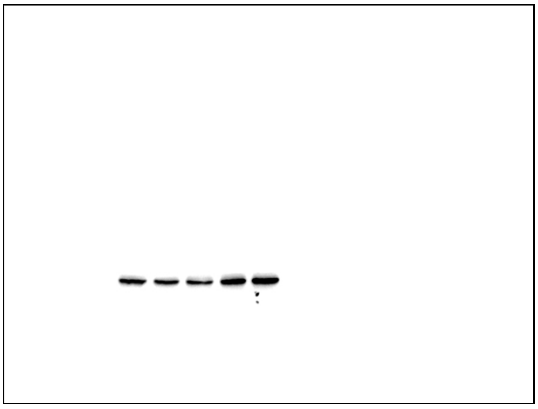

Caspase-12

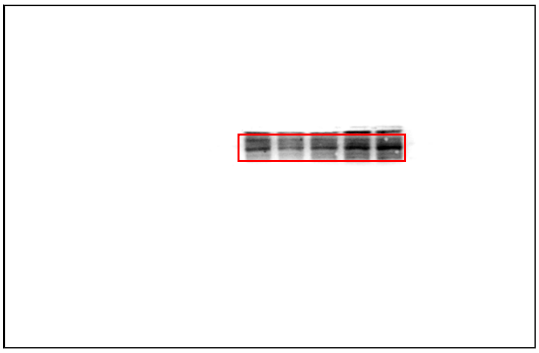

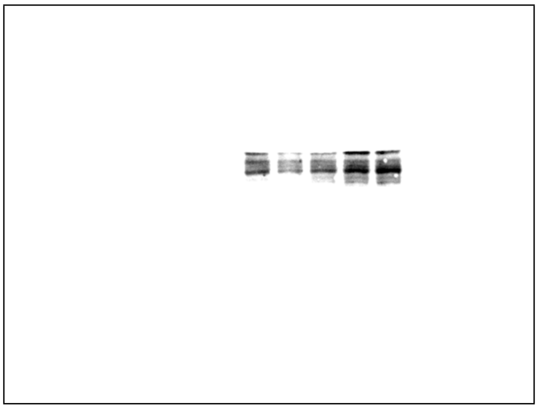

Cleaved caspase-3

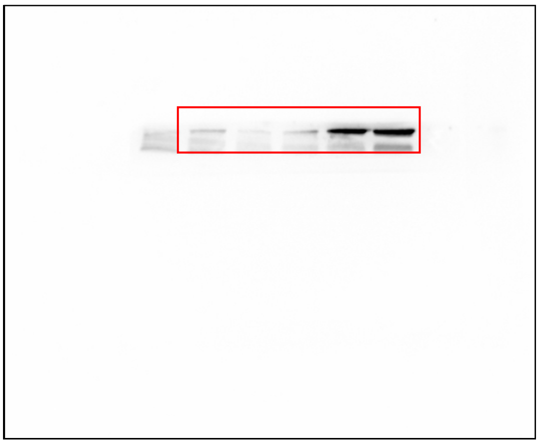

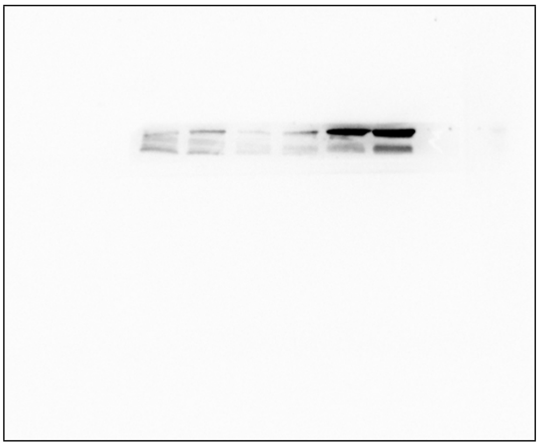

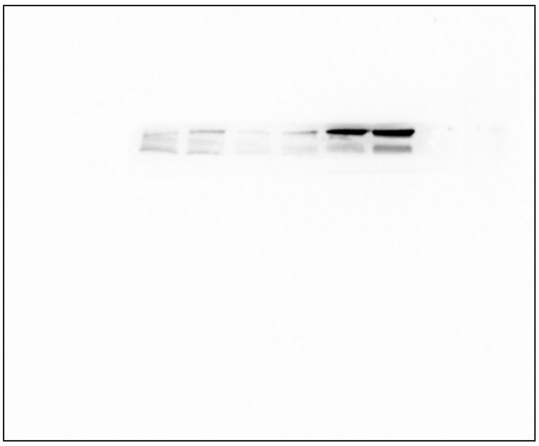

GAPDH

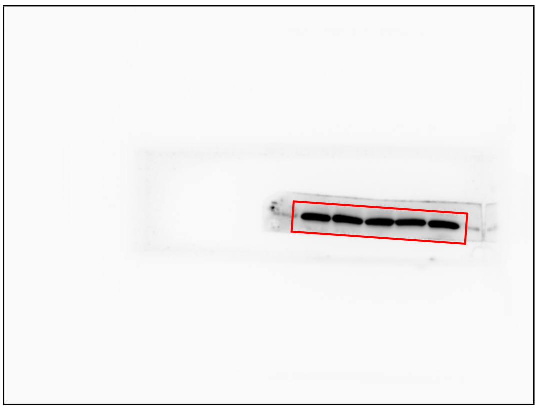

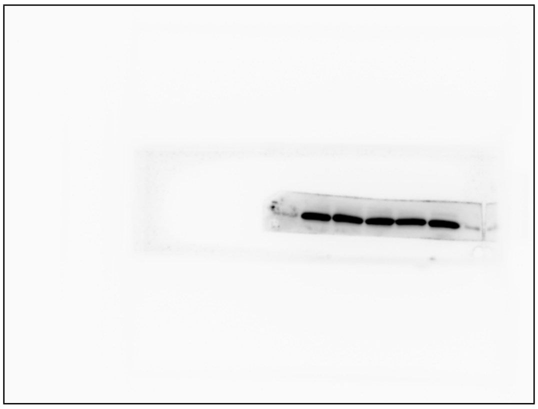

p-RelA

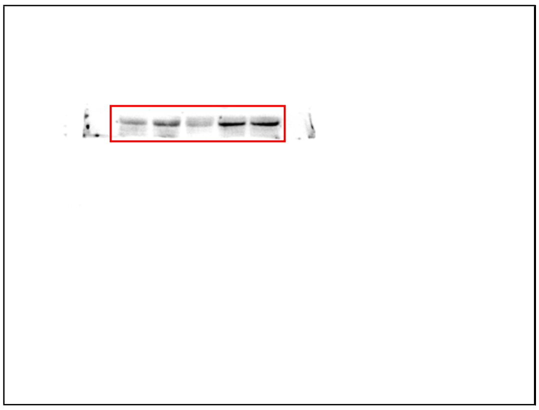

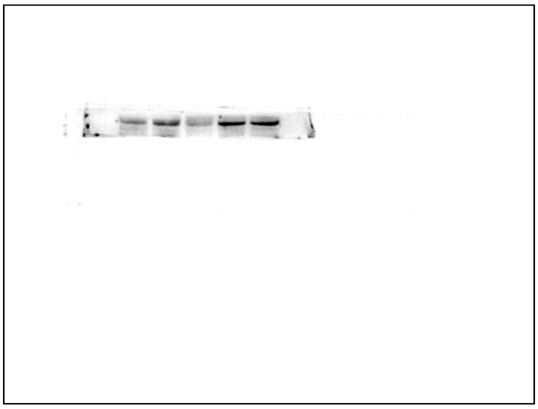

RelA

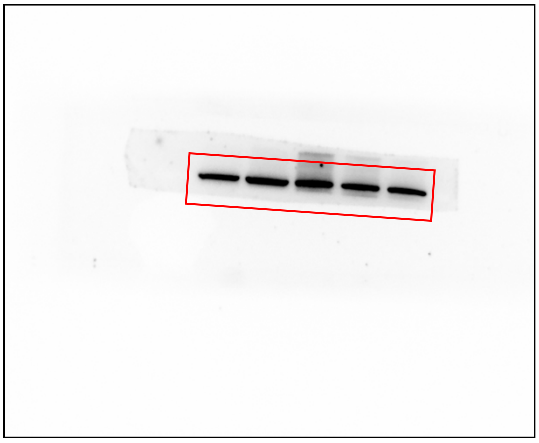

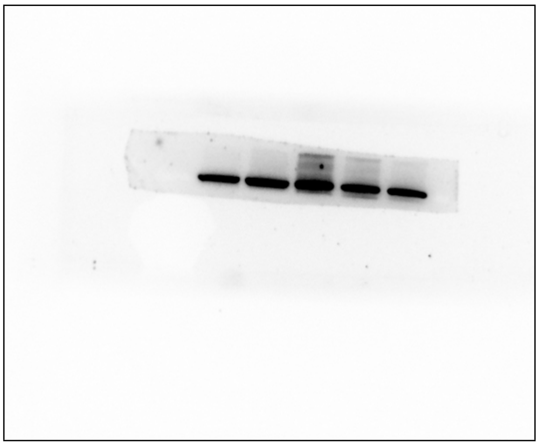

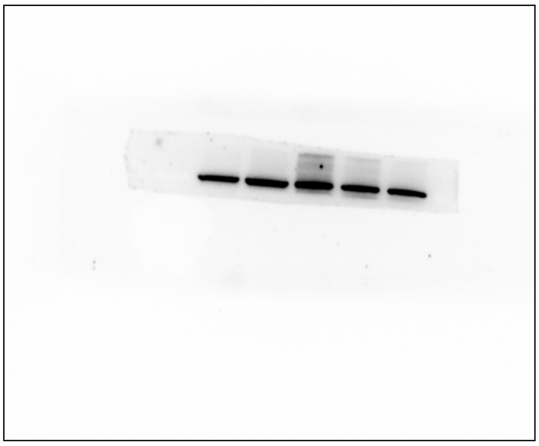

Figure 8D

HNF1α

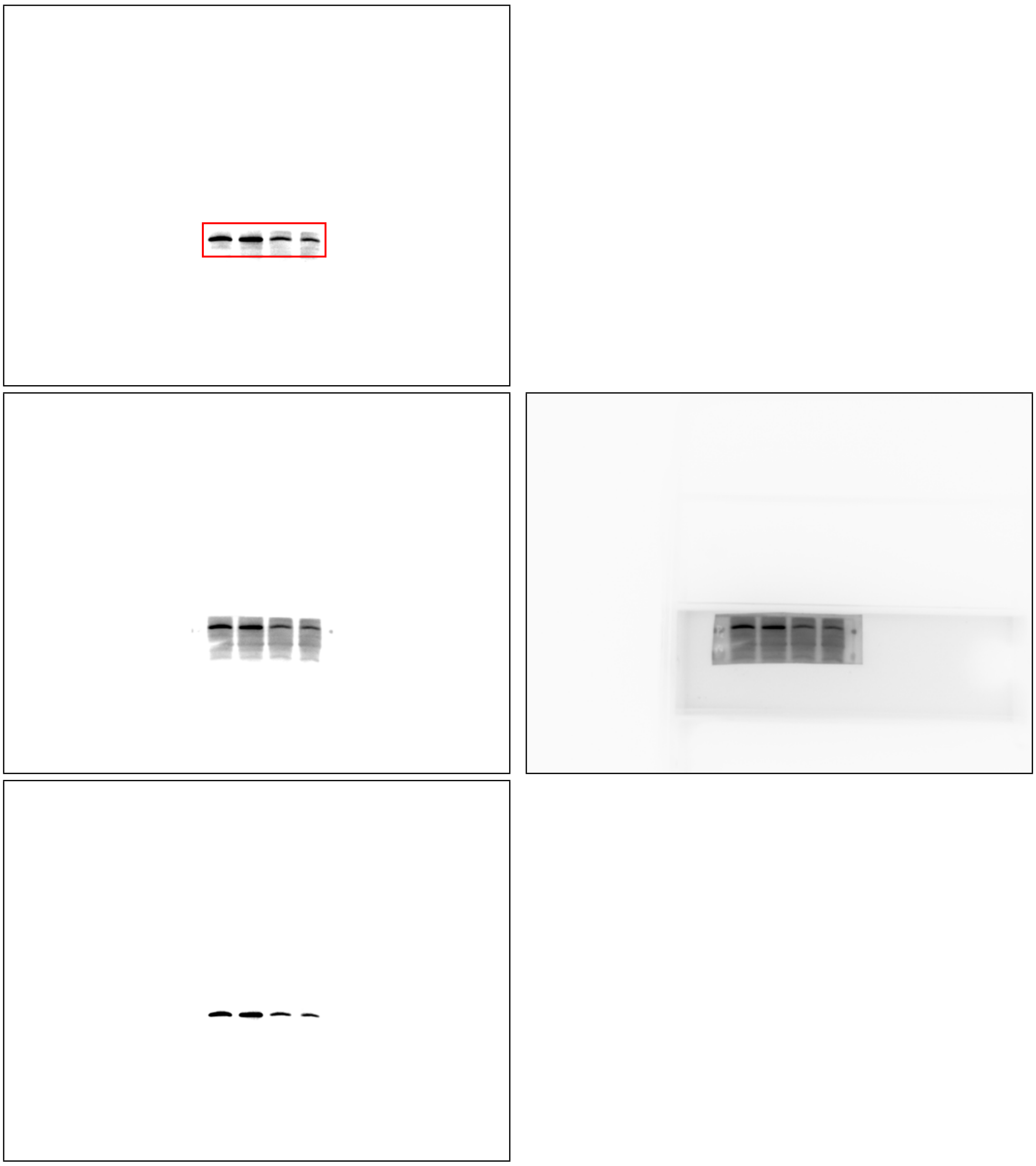

ATF4

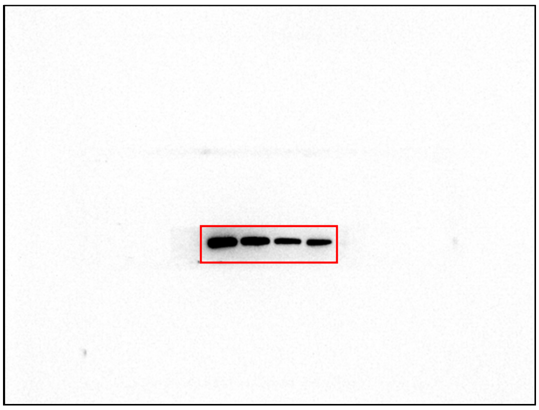

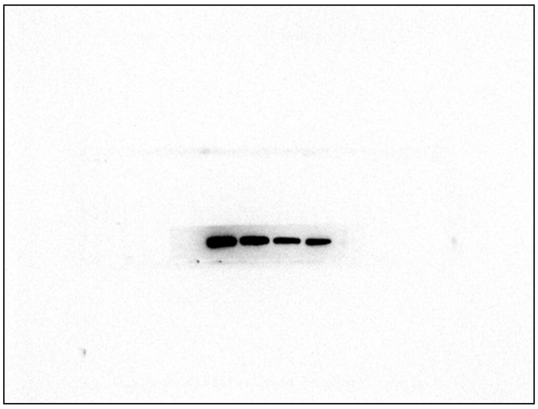

GRP78

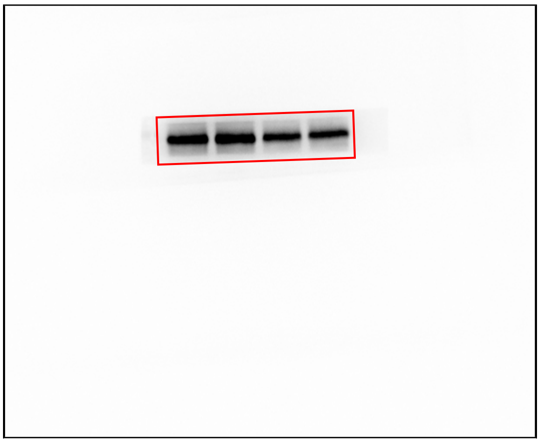

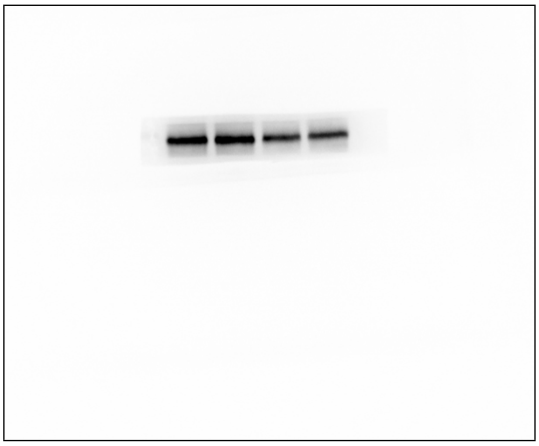

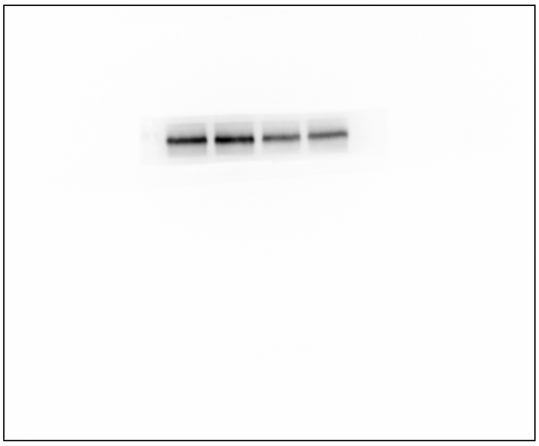

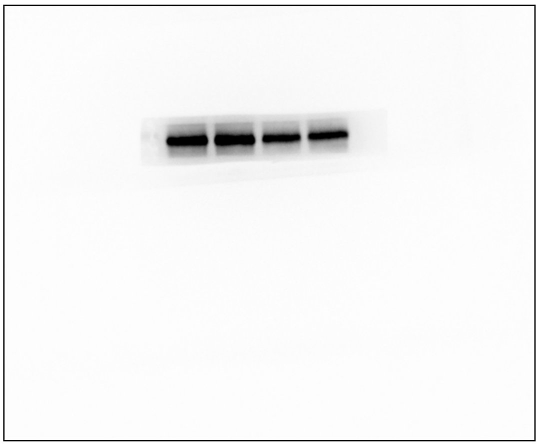

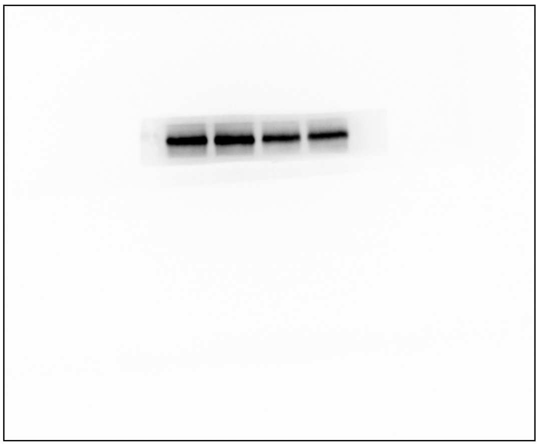

Caspase-12

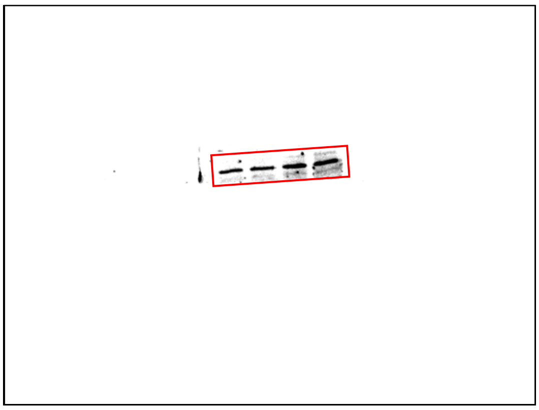

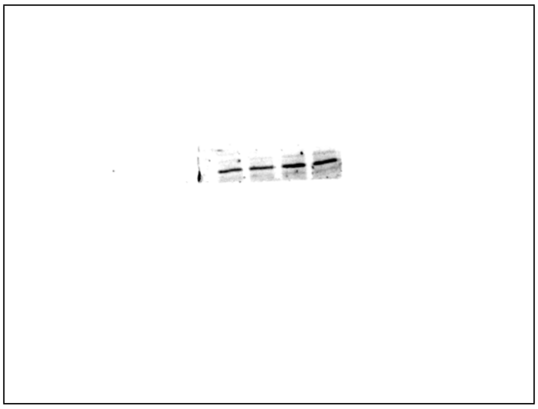

Cleaved caspase-3

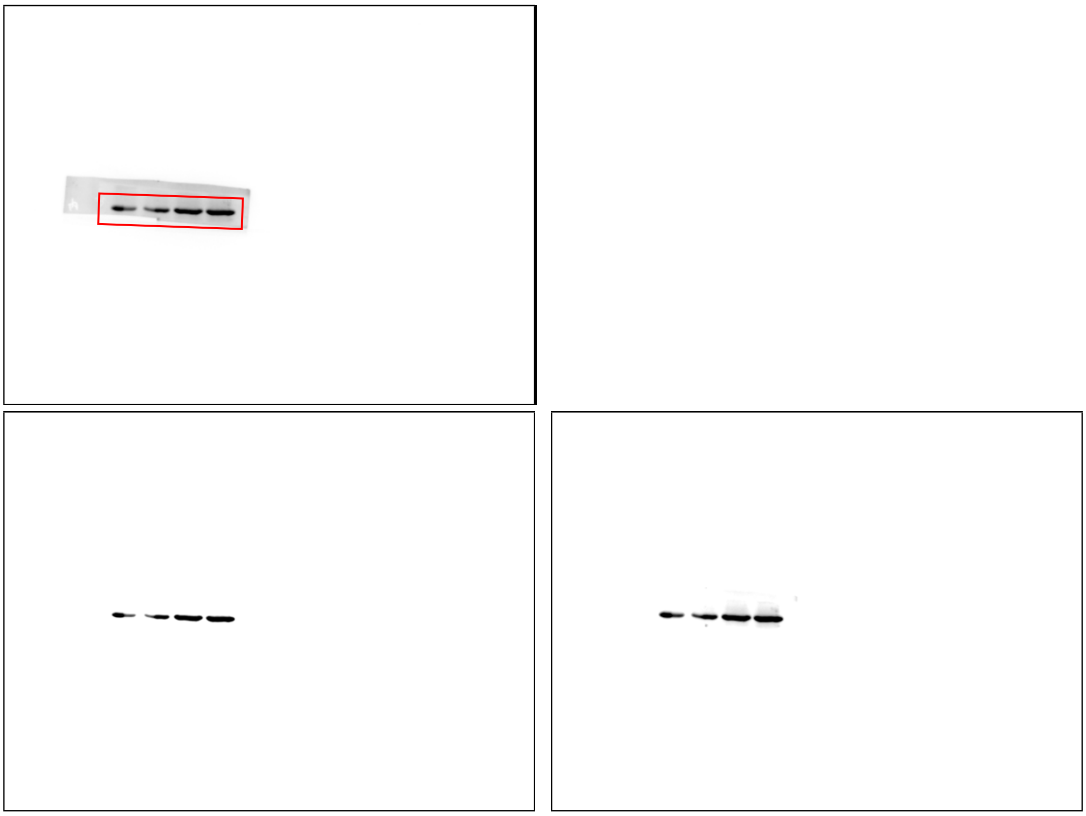

GAPDH

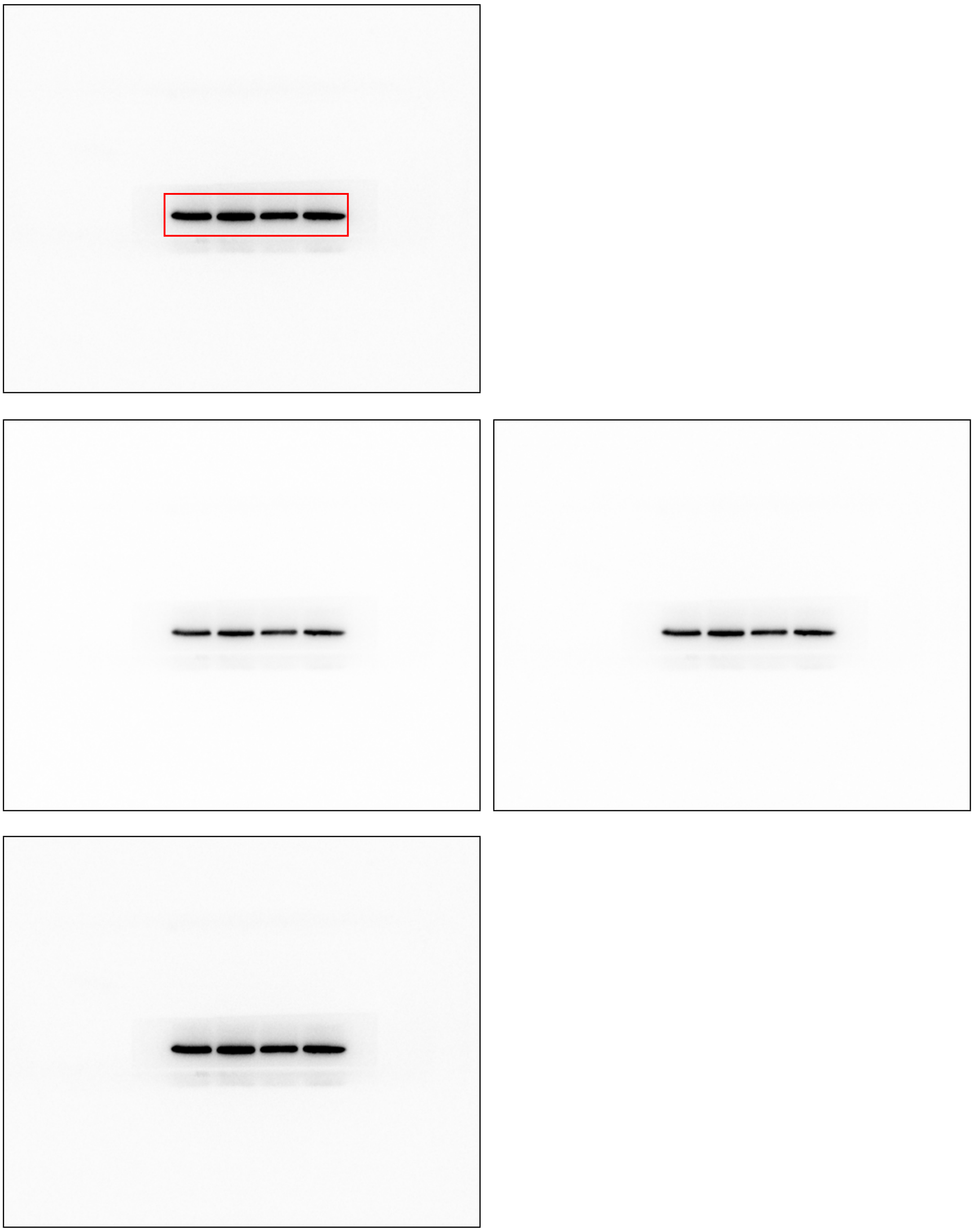

p-RelA

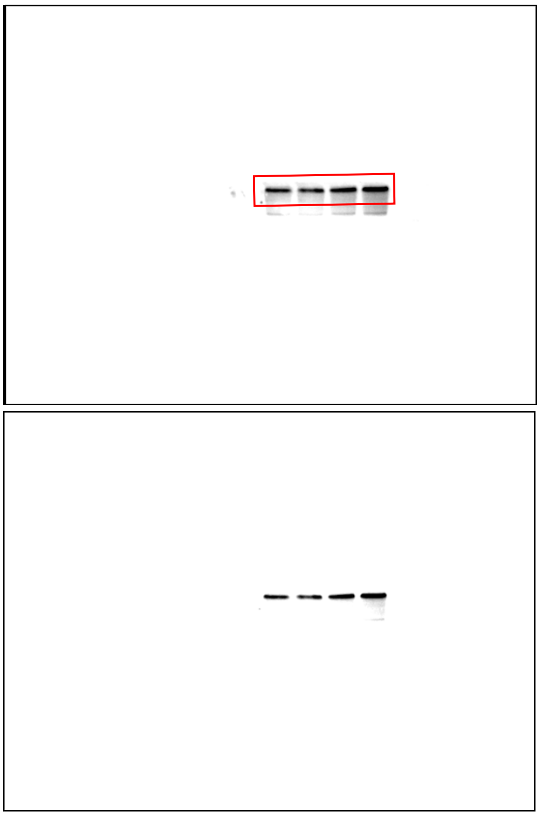

RelA

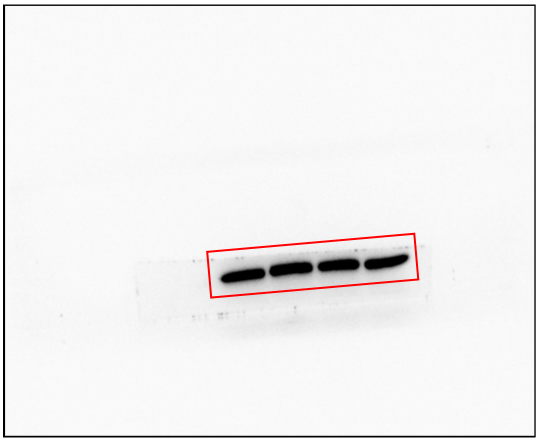

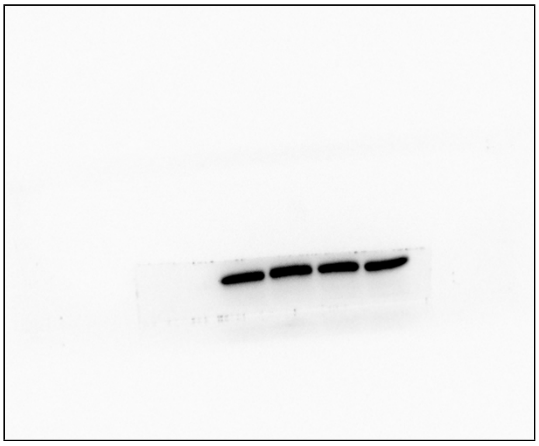

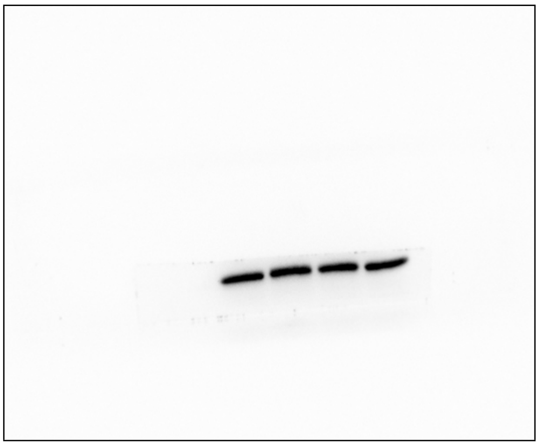

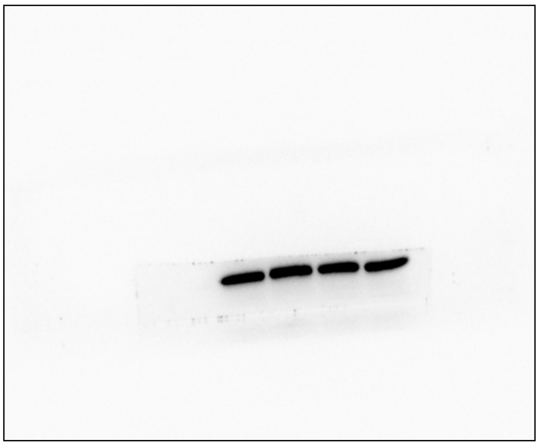

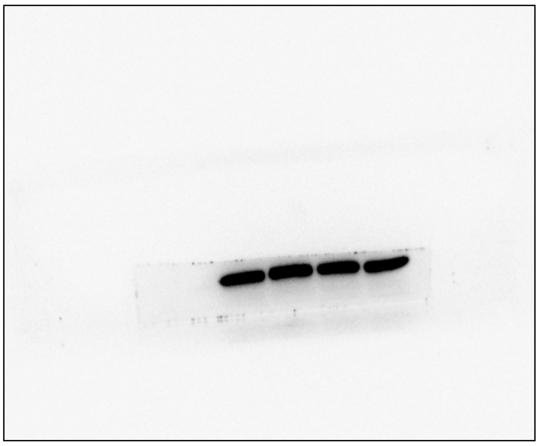

Supplement: Supplementary file 2 — Supplementary Information 2. [file 41598_2022_15846_MOESM2_ESM.pdf]
